# Efficacy and safety of adrenergic alpha-1 receptor antagonists in older adults: a systematic review and meta-analysis supporting the development of recommendations to reduce potentially inappropriate prescribing

**DOI:** 10.1186/s12877-022-03415-7

**Published:** 2022-09-28

**Authors:** Felix Mansbart, Gerda Kienberger, Andreas Sönnichsen, Eva Mann

**Affiliations:** 1 Department of General Practice and Family Medicine, Center for Public Health, Medical University of Vienna, Vienna, Austria; 2 Institute for Knowledge-Management in Medicine, Salzburg, Austria; 3 Institute of General Practice, Family Medicine and Preventive Medicine, Paracelsus Medical University, Salzburg, Austria

**Keywords:** Alpha-1 antagonists, LUTS, Hypertension, Benign prostatic hyperplasia, Inappropriate prescribing, Older people, Systematic review

## Abstract

**Background:**

Adrenergic alpha-1 receptor antagonists (alpha-1 antagonists) are frequently used medications in the management of lower urinary tract symptoms (LUTS) suggestive of benign prostatic hyperplasia (BPH) and in the management of therapy-resistant arterial hypertension, two conditions frequently found in older adults. This systematic review aims at presenting a complete overview of evidence over the benefits and risks of alpha-1 antagonist treatment in people ≥ 65 years, and at deriving recommendations for a safe application of alpha-1 antagonists in older adults from the evidence found.

**Methods:**

A comprehensive literature search was performed (last update March 25^th^ 2022) including multiple databases (Medline/Pubmed, Embase, the Cochrane Library) and using the PICOS framework to define search terms. The selection of the studies was done by two independent reviewers in a two-step approach, followed by a systematic data extraction. Quality appraisal was performed for each study included using standardised appraisal tools. The studies retrieved and additional literature were used for the development of recommendations, which were rated for strength and quality according to the GRADE methodology.

**Results:**

Eighteen studies were included: 3 meta-analyses, 6 randomised controlled trials and 9 observational trials. Doxazosin in the management of arterial hypertension was associated with a higher risk of cardiovascular disease, particularly heart failure, than chlorthalidone. Regarding treatment of LUTS suggestive of BPH, alpha-1 antagonists appeared to be effective in the relief of urinary symptoms and improvement of quality of life. They seemed to be less effective in preventing disease progression. Analyses of the risk profile indicated an increase in vasodilation related adverse events and sexual adverse events for some agents. The risk of falls and fractures as well as the effects of long-term treatment remained unclear. All meta-analyses and 5 out of 6 interventional studies were downgraded in the quality appraisal. 7 out of 9 observational studies were of good quality.

**Conclusions:**

It cannot be recommended to use doxazosin as first-line antihypertensive agent neither in older adults nor in younger patients. In the management of BPH alpha-1 antagonists promise to effectively relieve urinary symptoms with uncertainty regarding their efficacy in preventing long-term progression events.

**Supplementary Information:**

The online version contains supplementary material available at 10.1186/s12877-022-03415-7.

## Background

Alpha-1 antagonists are widely used agents predominantly in the treatment of arterial hypertension [1] and, since the late 1980s and early 1990s, LUTS suggestive of BPH [2–5]. Both conditions are very common worldwide [6, 7], especially prominent in the older population and expected to further increase in prevalence [8–10].

LUTS suggestive of BPH only concerns male patients, mostly older, and includes a number of symptoms such as frequency, nocturia, urgency, weak stream or interruption during micturition, high post-void residual volume in the urinary bladder or the difficulty initiating micturition [7]. Recent estimates indicate that almost 50% of men aged 50 years or older suffer from LUTS as a consequence of BPH and 80% of males over the age of 80 years [11]. In the US, prevalence numbers have developed substantially over the past years and it is expected that this trend will continue as the population ages [7].

A similar picture can be drawn for hypertension. According to Chow et al. [6] global prevalence figures for hypertension in adults are around 30% to 45% with this number being similar across the world and independent of the country’s income status. Age, however, does play an important role in the prevalence of hypertension as rates increase with progressing age. This results in shares of more than 60% in people with 60 years or more and about 75% in people over the age of 75 years [1]. It is currently forecasted that the number of people with hypertension will rise by 15% to 20% until 2025 resulting in approximately 1.5 billion affected people worldwide [8].

The use of alpha-1 antagonists in the therapy of hypertension is based on the modulation of vessel tone and systemic vascular resistance, which results in an increase in venous capacitance and lowering of blood pressure [12]. As alpha-1 adrenergic antagonists cause a relaxation of smooth muscle both in the vascular system and in the prostate [13], they are also effective in the therapy of LUTS suggestive of BPH reducing the symptoms by up to 50% [14]. Long-term studies showed no reduction in prostate size nor prevention of acute urinary retention events, though [15–17]. The most common adverse side effects include dizziness, postural hypotension, asthenia, headache, rhinitis and ejaculatory dysfunction occurring in about 5% to 9% of the patients [18].

The medical treatment of older adults comes with many challenges. On the one hand, it is known that pharmacodynamics as well as pharmacokinetics differ between younger patients and older patients [19] leading to an increased risk of developing ADEs among the elderly [20]. On the other hand, older adults are more frequently affected by multimorbidity, which may result in polypharmacy [21] and this again increases the risk of ADEs, adverse drug interactions, and possibly hospitalisation [22–24]. The versions of diverse national PIM (potentially inadequate medication for the elderly) lists are inconclusive on how to categorize alpha-1 antagonists. Doxazosin is included in three PIM lists [25–27], two of which also include terazosin [25, 27]. PIM lists of Austria, France and Canada do not include any of the alpha-1 antagonist [28–30].

In the light of the above-mentioned it seems overdue to summarize and synthesize the evidence available on the treatment of elderly with alpha-1 antagonists. An ageing population associated with an expected substantial increase of the prevalence of hypertension and LUTS suggestive of BPH in the near future will lead to an increased use of alpha-1 antagonists in patients older than 65 years. The aim of this systematic review is to explore the effectiveness and safety of alpha-1 antagonists in these patients and to develop recommendations on when to discontinue or reduce the dose of alpha-1 antagonists to prevent inappropriate prescribing.

To the best of our knowledge, so far, no systematic review has analysed the specific evidence on the use of alpha-1 antagonists in the aged population.

The aims of this SR are therefore to.

- systematically review the literature on the risks and benefits of the use of alpha-1 antagonists in older adults (≥ 65 years),
- critically assess the quality of evidence identified, and
- develop recommendations for or against the use of alpha-1 antagonists in older adults.

## Methods

This systematic review was developed and conducted with reference to the methodology as described in the Cochrane Handbook for Systematic Reviews of Interventions [31].

The study protocol was registered at the international prospective register of systematic reviews (CRD42020183345).

### Search method

A thorough review of existing research literature was carried out in a three-stage process. In the first stage, a highly sensitive search was performed in order not to miss out on any relevant studies. In steps two and three papers with irrelevant content according to the inclusion and exclusion criteria were removed.

#### Development of the search terms

For step one search terms were developed in accordance with the PICOS model for each of the following categories: population, intervention, comparison, outcomes and study design. The terms within each category were connected by “OR” in the search process while the terms of different categories were connected by “AND”. As Medline/Pubmed was used as a search engine, the official MeSH terms or its entry terms were applied as search terms (see Additional file 1 for full list of search terms).

#### Step 1 – literature search

The search was performed by a data research team at the University of Witten/Herdecke on the 19^th^ of June 2019, and updated on the 25^th^ of March 2022, in Medline/Pubmed, Embase and the Cochrane Database of Systematic Reviews using the OVID interface for each database. The result of the literature search was uploaded to Endnote X8.2 software for data management purposes. Duplicates were removed and step two (selection of studies) was performed with the help of Endnote.

Complementary to the literature search the citations of included studies were examined in order not to miss any important study.

#### Steps 2 & 3 – selection of studies

For the second step, two reviewers (FM and GK) worked through the list of research papers derived from step one by independently assessing the relevance of each study’s title and abstract. The assessment was based on the inclusion and exclusion criteria defined beforehand (for details, see below). If a paper seemingly met the inclusion criteria the study was included for step three. Research papers evidently not relevant for this systematic review were excluded. Upon conclusion of step two and before starting step three the reviewers compared their results. Studies selected by both were then automatically included in step three. If there were research papers selected by only one of the two, the reviewers had to re-evaluate and discuss to come to a mutual agreement. In case of an unresolvable disagreement a third reviewer (AS) was consulted and asked to decide.

The same procedure was applied to step three but now the assessment was based on the full manuscript of all studies selected in step two. The research papers selected in step three met all inclusion criteria and were therefore incorporated in this systematic review.

### Inclusion and exclusion criteria

#### Types of studies

The following types of studies were included: systematic reviews, meta-analyses, interventional studies and observational studies. Other study types were excluded, e.g. narrative reviews, expert opinions, case reports or letters.

#### Types of participants

Studies were only eligible if they reported results for older adults. The term older adults was defined as people with the age of 65 years or older. We included studies if the mean age minus 1.8 times the standard deviation was 65 years or older or if there was separate reporting for age subgroups equal to or greater than 65 years. In addition to the age criterion, we also defined patient conditions of interest for this systematic review (e.g. LUTS suggestive of benign prostatic hyperplasia and resistant arterial hypertension).

#### Types of interventions

Studies were only included if the intervention lasted for at least 4 weeks. Analyses on acute care and short-term treatment were thus excluded. In addition, the intervention had to include an alpha-1 antagonist (e.g. tamsulosin, doxazosin, alfuzosin) and a comparator. Such comparator could be no treatment, placebo, other drug (also different alpha-1 antagonists), phytotherapy or other non-pharmacological interventions. Studies without a comparator were excluded.

#### Types of outcomes

Studies were deemed eligible if they investigated direct patient relevant outcomes such as efficacy or effectiveness (e.g. change in the International Prostate Symptom Score [IPSS]) and/or ADEs (e.g. dizziness, asthenia, falls) and/or long-term drug safety (e.g. cognitive decline, cardiovascular events) as well as QoL, mortality, and/or hospitalisations. It was irrelevant for the inclusion of a study whether these outcomes were defined as primary or secondary outcomes. Studies only investigating surrogate parameters (e.g. asymptomatic changes in blood pressure as a proxy for orthostatic hypotension) were excluded.

#### Publication dates

No limit was set regarding publication dates.

#### Language

Studies were only included if they were written in English or German.

### Data extraction

All relevant data from included studies was extracted. Data was deemed relevant if it met all criteria to answer the research questions of this systematic review. It is therefore possible that only parts of the results of an entire study were extracted, e.g. subgroup analysis for study participants with the age of 65 years or older.

Standardised data collection forms were used for the data extraction. Results include tables for.

- the summary of characteristics of included studies,
- the summary of patient characteristics of included studies, and
- the summary of study findings of included studies.

Each of them is specific to the study designs included (i.e. meta-analyses, interventional studies, and observational studies). Data extraction was reviewed by a second researcher and checked for completeness, accuracy, and relevance.

#### Data synthesis/Meta-analyses

Due to high heterogeneity between the studies regarding interventions, comparators and outcomes meta-analyses were only performed with respect to six different outcomes: change in IPSS, change in QoL-score, the occurrence of ADEs, and incidence of dementia, falls and fractures. The meta-analyses on the incidence of dementia, falls and fractures are based on data on events per person (derived from events/1,000 person-years for two studies [32, 33]). The former two meta-analyses include data derived from three interventional studies [34–36]. While one of the studies provided values for mean and the 95% confidence interval (CI) for change figures [35], the other two studies reported mean scores and standard deviation (SD) for the baseline and final measurements but did not provide values for SD or CI of the changes [34, 36]. These figures were therefore imputed with reference to the suggestions described in the Cochrane Handbook for Systematic Reviews of Interventions [31]. For one study the p-value for change figures was used to obtain the t-values in order to calculate the standard error and finally the SD as the p-values were reported [36]. For the second study [34], the change-from-baseline SD from the study conducted by Gotoh et al. (2005) [35] was used as the study design for both studies was very similar and the p-value was not published. For the calculation of the meta-analyses the mean difference random effect model was used as all the studies used the same outcome scales (IPSS and QoL-score). Three interventional studies reported ADEs for tamsulosin and naftopidil [34–36], respectively, out of which one study conducted by Nishino et al. (2006) did not report any ADEs for neither of the interventions [36]. It was therefore decided to exclude this study from the meta-analysis as suggested in the Cochrane Handbook for Systematic Reviews of Interventions [31]. The Mantel–Haenszel method was used due to the low number of events. Review Manager, version 5.3, was used for computations and the creation of figures [37].

### Quality appraisal

A critical assessment of the methodological quality was performed for each included study in duplicate following the same logic as for the selection of studies. Established and validated appraisal tools were therefore used depending on the respective study type. Interventional studies were evaluated with the Revised Cochrane Risk-of-Bias Tool for Randomised Trials (RoB 2) [38]. Observational studies were assessed by using the checklists offered by Critical Appraisal Skills Programme (CASP) on the critical appraisal of case–control studies [39] and cohort studies [40]. Systematic reviews including meta-analyses were assessed with AMSTAR 2, an instrument developed for the measurement and assessment of systematic reviews [41].

### Development of recommendations

Recommendations on the use of alpha-1 antagonists in patients aged ≥ 65 years were developed based on the findings of this systematic review supplemented with additional references. A specific non-systematic literature search was therefore performed in the Cochrane Database of Systematic Reviews and Medline/Pubmed including papers found during steps two and three of the search for this systematic review and studies found by snowballing. In addition, the most recent evidence based guidelines for the treatment of hypertension by the European Society of Hypertension (ESH) and the European Society of Cardiology (ESC) [42] and for the treatment of non-neurogenic male LUTS by the European Association of Urology (EAU) [43] were consulted and its references screened. Each recommendation was rated with respect to strength (weak or strong) and quality (high, moderate, low, very low) [44–46] according to the Grading of Recommendations Assessment, Development and Evaluation (GRADE) methodology.

## Results

### Results of the search

**Fig. 1:**
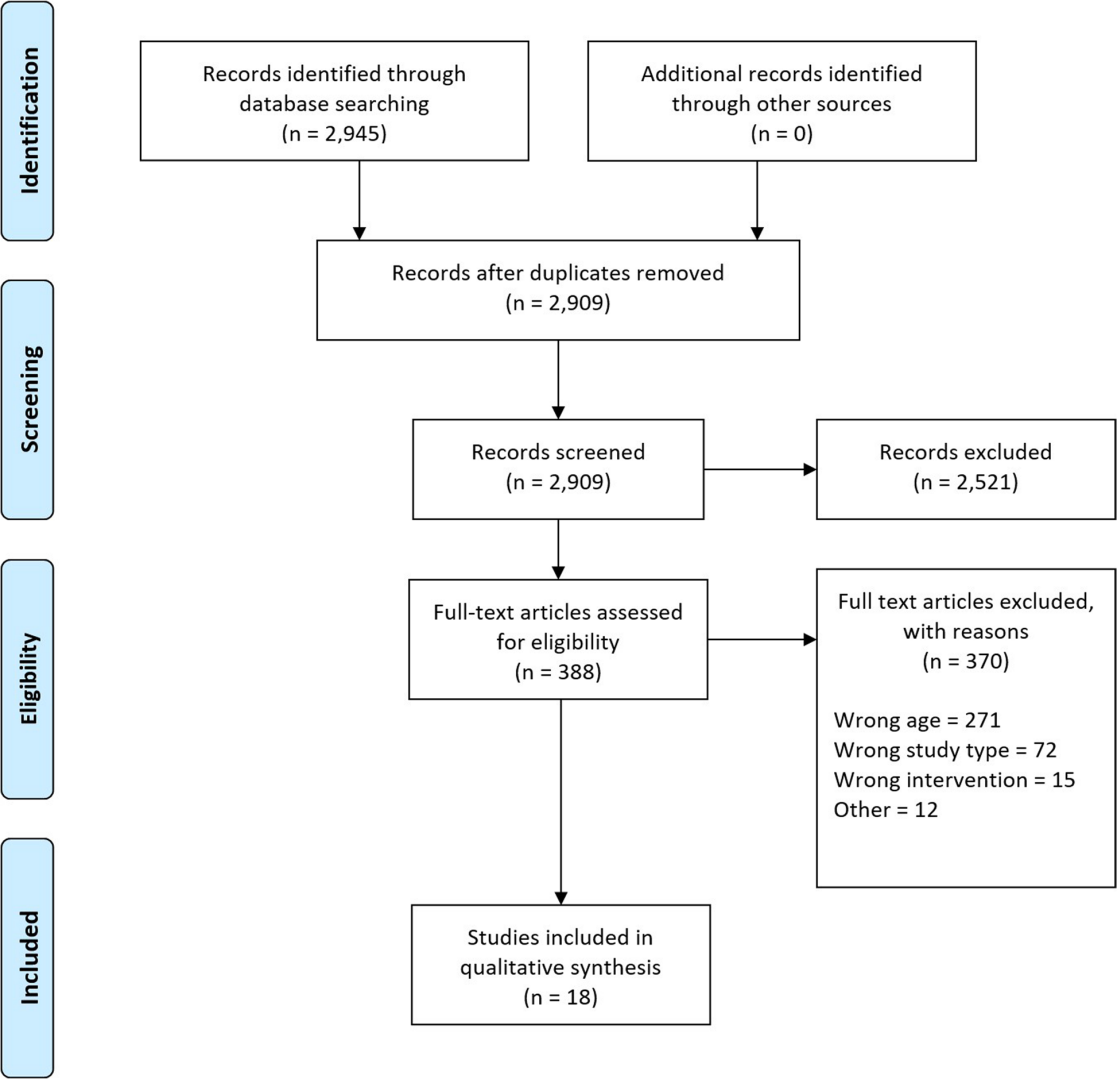
Preferred reporting items for systematic reviews and meta-analyses (PRISMA) flow diagram

Two thousand nine hundred forty-five records could be identified through the first step of the systematic review process, out of which 36 duplicates were immediately removed. No additional records were identified through other sources. 2909 research papers were screened during the second step, out of which 2521 could be excluded based on title and abstract. The remaining 388 records were assessed for eligibility based on full texts. 370 full texts were excluded in step three. We included 15 primary studies (6 RCTs [34–36, 47–49], 9 observational studies [32, 33, 50–56]) and three meta-analyses [57–59]. As opposed to the original studies [60–64], the meta-analyses reported the results for relevant age-related subgroups by using unpublished data. Their references were thus excluded throughout the search process. Additional file 2 lists all research papers excluded in the last step individually with the respective reason for exclusion. Figure 1 shows the PRISMA flow diagram [65].

### Characteristics of included studies

Eighteen studies were included in this systematic review, out of which six are interventional studies [34–36, 47–49], nine are observational studies, three cohort studies [32, 33, 50, 51, 54–56] and two case–control studies [52, 53], and three are meta-analyses, two including two [57, 59] and one including six [58] randomised controlled trials. The trials’ geographical focus was mainly on the United States of America (US), Canada, European countries and Japan. Follow-up was minimum 4 weeks [36, 57] and the longest lasting trial had a maximum follow-up period of 13 years [55]. For a detailed summary of the characteristics of included studies please refer to Table 1.

**Table 1:** Summary of characteristics of included studies

Authors (year) place	Study type	Intervention and Comparator	Sample size and age profile	Inclusion criteria	Follow-up period	Measured outcome	Sponsors
ALLHAT (2003) [47] US, CA, PR	Randomized double-blind controlled trial	Doxazosin 2, 4 or 8 mg/d vs. chlorthalidone 12.5 or 25 mg/d	Doxazosin: *n* = 9,061 ≥ 70 y: 3,092 Chlorthalidone: *n* = 15,255 ≥ 70 y: 5,410	Age ≥ 55 y RR_sys_ ≥ 140 mmHg or RR_dia_ ≥ 90 mmHg or antihypertensive medication ≥ 1 additional risk factor for cardiac heart disease (CHD)	Mean: 3.2 years	*Primary outcome:* combined incidence of fatal CHD or nonfatal myocardial infarction *Secondary outcome:* mortality, combined CHD, stroke, combined cardiovascular disease (CVD)	National Heart, Lung, and Blood Institute (US) Medications by Pfizer Inc, AstraZeneca, Bristol-Myers Squibb Financial support by Pfizer Inc
Buzelin et al. (1997) [57] FR, BE, DE, NL, DK	Meta-analysis based on 2 RCTs	SR alfuzosin 2 × 5 mg/d vs. placebo	SR alfzusosin: *n* = 292 ≥ 65 y: 149 Placebo: *n* = 296 ≥ 65 y: 153	LUTS due to BPH > 6 months Nocturia: ≥ 2 and/or Day-time frequency: ≥ 8 Q_max_: ≤ 15 ml/s Voiding volume: > 150 ml	1 month	All ADEs with a special focus on ADEs related to vasodilatory events	N.a.
Chapple et al. (1997) [59] BE, DE, DK, NL, NO, SE, UK	Meta-analysis based on 2 double-blind RCTs	Tamsulosin 0.4 mg/d vs. placebo	Tamsulosin: < 65 y: 190 ≥ 65 y: 191 Placebo: < 65 y: 93 ≥ 65 y: 100	Age ≥ 45 y LUTS due to BPH 4 ml/s ≤ Q_max_ ≤ 12 ml/s Voiding volume: ≥ 120 ml Boyarsky score > 6	12 weeks	ADEs	Yamanouchi Europe BV
Chrischilles et al. (2001) [50] US	Retrospective cohort study	Alpha-1 antagonist users (terazosin/ doxazosin/ prazosin) vs. non-users	Users: *n* = 1,564 Mean age: 73 y Prazosin = 15 Doxazosin = 782 Terazosin = 839 Non-Users: *n* = 8,641 Mean age: 72.5 y	Age ≥ 65 y Diagnosed BPH	4 months	Possibly hypotension related adverse effects (e.g. hypotension, syncope, dizziness, falls)	Grant from Boehringer Ingelheim
Duan et al. (2018) [33] US	Retrospective Cohort Study	Tamsulosin vs. each of the following: No BPH medication Doxazosin Terazosin Alfuzosin Dutasteride Finasteride	Tamsulosin: *n* = 253,136 No BPH-medication: *n* = 180,926 Doxazosin: *n* = 28,581 Terazosin: *n* = 23,858 Alfuzosin: *n* = 17,934 Dutasteride: *n* = 34,027 Finasteride: *n* = 38,767	Age ≥ 66 y Diagnosed BPH	Median follow-up: 19.8 months	Incidence of dementia	Connecticut Institute for Clinical and Translational Science Patient-Centred Outcome Research Trust Fund
Gotoh et al. (2005) [35] JP	Randomized controlled trial	Tamsulosin 0.2 mg/d vs. naftopidil 50 mg/d	Tamsulosin: *n* = 75 mean age: 68.5 y 95% CI: 67.0 – 70.1 y Naftopidil: *n* = 69 mean age: 68.0 y 95% CI: 66.4 – 69.8 y	Age ≥ 50 y Symptomatic BPH IPSS ≥ 8 Q_max_ < 15 ml/s Voiding volume ≥ 150 ml V_prostate_: ≥ 20 ml	12 weeks	*Primary outcomes:* changes in IPSS, Q_max_ and residual urine volume *Secondary outcomes:* average flow rate, changes in IPSS storage score and IPSS voiding score, quality-of-life score	N.a.
Hall and McMahon (2007) [52] UK	Case–control study	Fracture vs. no fracture	Cases (fracture): *n* = 6,540 Taking MR Doxazosin: 66 Taking MR Doxazosin and ≥ 75y: 32 Controls (no fracture): *n* = 26,495 Taking MR Doxazosin: 311 Taking MR Doxazosin and ≥ 75y: 173	Age ≥ 50 y Cases: Fracture of hip/femur, humerus, wrist Mean age: 74 y Controls: No fracture Mean age: 73 y Matched with Cases on primary care practice, year of birth and sex	Mean (SD) days of observation: Cases: 569 (344) Controls: 569 (344)	Incidence of fracture of hip, femur, humerus and/or wrist	Pfizer UK
Hiremath et al. (2019) [54] CA	Retrospective cohort study	Alpha-1 antagonist users (terazosin, prazosin, doxazosin) vs. other BP lowering medication users	Users: *n* = 14,106 Mean age: 75.7 y Non-Users: *n* = 14,106 Mean age: 75.7 y	Age ≥ 66 y Only women No previous alpha-1 antagonist use < 6 BP lowering medications	12 months	*Primary outcomes:* hypotension and related events (syncope, falls, fractures) *Secondary outcomes:* adverse cardiac events, all-cause mortality	Supported by the Institute for Clinical Evaluative Sciences (ICES) ICES is funded by Ontario Ministry of Health and Long-Term Care Data partly provided by CIHI
Hundemer et al. (2021) [32] CA	Retrospective cohort study	Alpha-1 antagonist users (terazosin, prazosin, doxazosin) vs. other BP lowering medication	Exposed: *n* = 16,088 Mean age: 75 y Not exposed: *n* = 16,088 Mean age: 75 y	Age ≥ 66 y Diagnosis of hypertension New prescription for alpha-1 antagonists/ other BP lowering drug	Max. 3 y	Adverse kidney events, cardiac events, all-cause mortality, ADEs (hypotension, syncope, falls, fractures)	Supported by the Institute for Clinical Evaluative Sciences (ICES) ICES is funded by Ontario Ministry of Health and Long-Term Care Data partly provided by CIHI
Lowe (1994) [58] US, EU	Meta-analysis based on 6 double-blind RCTs	Terazosin 1–20 mg/d vs. placebo	Terazosin: *n* = 636 ≥ 65 y: 285 Placebo: *n* = 360 ≥ 65 y: 162	Symptomatic BPH Q_max_: ≤ 12 ml/s Min. voiding volume: 100–150 ml	2–6 months	ADEs	Grant from Abbott Laboratories
Nishino et al. (2006) [36] JP	Randomized crossover trial	Tamsulosin 0.2 mg/d vs. naftopidil 50 mg/d	Tamsulosin/ naftopidil: *n* = 17 Naftopidil/tamsulosin: *n* = 17	Age ≥ 66 y Symptomatic BPH IPSS ≥ 8 Q_max_: ≤ 15 ml/s No prior treatment for BPH	9 weeks (two 4-week trials for each substance and a 1-week washout in between)	IPSS, QoL score, uroflowmetry, pressure flow study	Gifu University, JP
Oelke et al. (2014) [48] AU, AT, BE, FR, DE, GR, IT, MX, NL, PL	Randomized double-blind placebo-controlled trial	Tamsulosin 0.4 mg/d vs. tadalafil 5 mg/d vs. placebo	Tamsulosin: *n* = 168 ≥ 66 y: 72 Tadalafil: *n* = 171 ≥ 66 y: 75 Placebo: *n* = 172 ≥ 66 y: 77	Age ≥ 45y Symptomatic BPH > 6mo IPSS ≥ 13 Q_max_: 4–15 ml/s PVR < 300 ml No treatment with finasteride within 3mo No treatment with dutasteride within 6mo	12 weeks	Treatment Satisfaction Scale-BPH (TSS-BPH)	Eli Lilly and Company Personal fees from diverse pharma-ceutical companies for authors Three authors employed by Eli Lilly and Company during conduct of study
Roehrborn (2006) [49] North America, Australia, Middle East, South Africa, Europe	Randomized double-blind placebo-controlled trial	Alfuzosin 10 mg/d vs. placebo	Alfuzosin: *n* = 759 ≥ 65 y: 449 Placebo: *n* = 763 ≥ 65 y: 439	Age ≥ 55y Symptomatic BPH ≥ 6mo IPSS ≥ 13 Q_max_: 5–12 ml/s Voiding volume ≥ 150 ml PVR ≥ 350 ml V_prostate_: ≥ 30 g	24 months	*Primary outcome:* Occurrence of first period of acute urinary retention *Secondary outcomes:* BPH-related surgery, total IPSS, bother score, Q_max_	Sanofi-Aventis
Siemens et al. (2021) [55] CA	Retrospective cohort study	No medication vs. dutasteride or finasteride (5-ARI) use vs. silodosin or tamusosin (selective) or terazosin or alfuzosin or doxazosin (non-selective alpha-1 antagonist) use vs. 5-ARI + alpha-1 antagonist use	No medication: *n* = 69,988 Mean age: 74.05 y Alpha-1 antagonist: *n* = 55,383 Mean age: 74.26 y 5-ARI + alpha-1 antagonist: *n* = 41,491 Mean age: 74.11 y	Age ≥ 66 y Diagnosis of BPH No recent history of cardiac failure	Max. 13 y	Incidence of heart failure	Supported by the Institute for Clinical Evaluative Sciences (ICES) ICES is funded by Ontario Ministry of Health and Long-Term Care Data provided by CIHI
Tae et al. (2019) [56] KR	Retrospective cohort study	Analysis 1: Tamsulosin vs. each of the following: Doxazosin Terazosin Alfuzosin Analysis 2: No medication vs. each of the following: Tamsulosin Doxazosin Terazosin Alfuzosin	No medication: *n* = 3,336 Mean age: 77.03 y Tamsulosin: *n* = 33,568 Mean age: 76.47 y Doxazosin: *n* = 7,012 Mean age: 76.54 y Terazosin: *n* = 9,443 Mean age: 76.73 y Alfuzosin: *n* = 5,904 Mean age: 76.14 y	Age ≥ 70 y Diagnosis of BPH No factors associated with cognitive decline (e.g. chemotherapy, anticholinergic drug use or psychiatric disease)	Mean (SD) days of follow up: 1,580 (674)	Incidence of dementia	Grant from Korea University and the Korea Urologic Association
Testa et al. (2018) [53] IT	Case–control study	Syncopal fall vs. non-syncopal fall	Syncopal fall: *n* = 354 Mean age: 83.3 y Non-syncopal fall: *n* = 168 Mean age: 83.9 y	Age ≥ 65 y Dementia ≥ 1 transient loss of consciousness or unexplained fall within previous 3 months	3 months	Orthostatic hypotension related syncopal falls	Endorsement of Italian Society of Gerontology and Geriatrics
Welk et al. (2015) [51] CA	Retrospective cohort study	Tamsulosin, silodosin, alfuzosin vs. no alpha-blocker treatment	Alpha-blocker initiation: *n* = 147,084 No initiation: *n* = 147,084	Age ≥ 66 y Alpha-1 antagonist cohorts: Initiation of first treatment with tamsulosin, silodosin, or alfuzosin Non-alpha-1 antagonist cohort: Unexposed to alpha-blocker Matched on age, residential status, prior fractures, use of 5α-reductase inhibitors	90 days	*Primary outcome:* hospitalization for a fall or fracture within 90 days after initiation of alpha-blocker therapy *Secondary outcomes:* hypotension, head trauma	Institute for Clinical Evaluative Sciences (Ontario Ministry of Health and Long-Term Care)
Yokoyama et al. (2011) [34] JP	Randomized controlled trial	Tamsulosin 0.2 mg/d vs. silodosin 8 mg/d vs. naftopidil 50 mg/d	Tamsulosin: *n* = 45 mean age: 71.5 y Silodosin: *n* = 45 mean age: 70.2 y Naftopidil: *n* = 46 mean age: 69.1 y	Age ≥ 50 Symptomatic BPH PSS ≥ 8	12 weeks	IPSS, quality-of-life score, International Index of Erectile Function (IIEF-5), Q_max_, PVR	N.a.

Abbreviations: *5-ARI* 5-alpha reductase inhibitor, *ADE* adverse drug event, *BP* blood pressure, *BPH* benign prostatic hyperplasia, *CHD* cardiac heart disease, *CI* confidence interval, *d* day, *HR* hazard ratio, *IPSS* international prostate symptom score, *N.a.* not available, *OR* odds ratio, *PVR* post-void residual volume, *Q*_*max*_ maximum urinary flow rate, *RR* relative risk, *RR*_*sys*_ systolic blood pressure, *RR*_*dia*_ diastolic blood pressure, *SD* standard deviation, *y* years

### Characteristics of study participants

The age structure of participants varied as inclusion criteria were defined differently between studies. All three meta-analyses [57–59] and three interventional studies [47–49] had a broad age distribution but offered age related subgroup analyses. Two interventional studies [34, 35] set minimum age below 65 years but were included entirely as they met the age eligibility criteria as defined above and one interventional study [36] enrolled only patients aged 66 years or older. Eight observational trials only included older adults [32, 33, 50, 51, 53–56], one looked at younger patients also but presented relevant age-related content [52]. See Additional file 3 for a summary of the patient characteristics of included studies. Refer to Additional file 4 for further details on patient characteristics for each study used in the included meta-analyses.

### Interventions and outcomes

#### Doxazosin

Two studies focused particularly on doxazosin [47, 52]. The ALLHAT study (2003) compared the efficacy of doxazosin and chlorthalidone in reducing cardiovascular events (e.g. fatal coronary heart diseas or combined cardiovascular disease) in hypertensive patients [47]. Hall and McMahon (2007) performed a retrospective observational study and investigated the relation of exposure to doxazosin and the incidence of fractures (e.g. hip, femur) [52].

#### Tamsulosin, naftopidil and silodosin

Four interventional studies examined the efficacy of tamsulosin [34–36, 48], whereas two observational studies [33, 51] and one meta-analysis [59] explored particular side effects. Oelke et al. (2014) compared treatment satisfaction between tamsulosin and placebo or tadalafil in patients with urinary symptoms related to BPH [48]. Three further interventional studies assessed the efficacy of tamsulosin against naftopidil [34–36] and silodosin [34] with regard to the reduction of urinary symptoms according to the IPSS, the increase of QoL and, with the exception of Yokoyama et al. (2011) [34], the occurrence of ADEs. Two meta-analyses could be performed comparing pre to post drug administration data for tamsulosin with regard to change in IPSS and QoL [34–36]. One additional meta-analysis was performed to compare the occurrence of ADEs between tamsulosin and naftopidil [34, 35]. Chapple et al. (1997) compared the safety and tolerability of tamsulosin with placebo in older patients with LUTS suggestive of BPH [59]. Duan et al. (2018) and Tae et al. (2019) explored the association of tamsulosin use and the risk of dementia by comparing to no BPH medication and alternative treatment options [33, 56]. Welk et al. (2015) analysed the occurrence of falls in tamsulosin users [51].

#### Alfuzosin

Roehrborn (2006) explored the occurrence of progression events due to BPH (i.e. worsening of IPSS, BPH related surgery and acute urinary retention events) in patients taking alfuzosin 10 mg per day controlled against placebo over a period of 24 months [49]. A meta-analysis by Buzelin et al. (1997) analysed the incidence of ADEs from two randomised controlled trials [57].

#### Terazosin

Lowe et al. (1994) performed a meta-analysis and therefore amalgamated the data of six randomised controlled trials to assess the safety of terazosin use in the treatment of BPH related symptoms [58].

#### Any alpha-1 antagonist

Six observational studies included several alpha-1 antagonists in their analysis partly without differentiating between single agents. Chrischilles et al. (2001) and Hiremath et al. (2019) examined the effect of treatment initiation with terazosin, doxazosin or prazosin on hypotension related adverse events [50, 54]. Hundemer et al. (2021) examined the association of alpha-1 antagonist use and adverse kindey or cardiac events, mortality and safety-related outcomes [32]. Testa et al. (2018) analysed the role of antihypertensive drugs including alpha-1 antagonists in the occurrence of orthostatic hypotension related syncopes in people with dementia [53]. Welk et al. (2015) elaborated on the effect of treatment initiation with tamsulosin, alfuzosin or silodosin on the risk for fractures and falls [51], and Siemens et al. (2021) investigated the association of alpha-1 antagonist treatment and cardiac failure [55].

### Main findings

The results of the included studies are structured and summarised by outcome below. Please refer to Table 2 for a summary of study findings and to Additional file 5 for a detailed display of the study results.

**Table 2:** Summary of study findings of included studies

Authors (Year) Study type Critical quality appraisal rating	Drug vs. comparator	Outcome	Relative Risk (RR)/Odds Ratio (OR)/Hazard Ratio (HR)	Events (%) or mean score (SD) or mean difference (95% CI)	*p*-value
ALLHAT (2003) [47] RCT RoB rating: low risk	Doxazosin vs. chlorthalidone	Combined CVD < 65 y	RR: 1.15 (1.04–1.27)		< 0.05
	Doxazosin vs. chlorthalidone	Combined CVD ≥ 65 y	RR: 1.23 (1.14–1.32)		< 0.05
	Doxazosin vs. chlorthalidone	Heart failure < 65 y	RR: 1.76 (1.40–2.22)		< 0.05
	Doxazosin vs. chlorthalidone	Heart failure ≥ 65 y	RR: 1.89 (1.65–2.17)		< 0.05
Buzelin et al. (1997) [57] Meta-analysis based on 2 RCTs RoB rating: high risk	Alfuzosin vs. placebo	ADEs ≥ 65 y		12/149 (8.1%) vs 12/153 (7.8%)	> 0.05
	Alfuzosin vs. placebo	ADEs related to vasodilation ≥ 65 y		2/149 (1.3%) vs 2/153 (1.3%)	> 0.05
Chapple et al. (1997) [59] Meta-analysis based on 2 double-blind RCTs RoB rating: high risk	Tamsulosin vs. placebo in the age group ≥ 65 y	Any adverse event		70/191 (37%) vs 31/100 (31%)	0.330
	Tamsulosin vs. placebo in the age group ≥ 65 y	Drug related adverse event ^b^		23/191 (12%) vs 9/100 (9%)	0.459
	Tamsulosin vs. placebo in the age group ≥ 65 y	Adverse events possibly associated with vasodilation ^c^ and drug related ^b^		7/191 (3.7%) vs 3/100 (3%)	0.767
	Tamsulosin vs. placebo in the age group ≥ 65 y	Abnormal ejaculation		5/191 (2.6%) vs 1/100 (1.0%)	0.668
Chrischilles et al. (2001) [50] Retrospective cohort study Quality appraisal: low quality	α1-blocker treatment vs. no α1-blocker treatment	Compare no. of ADEs/10,000 person-days 4 months pre- to 4 months post initiation		2.82 to 4.64 vs 3.62 to 3.60	0.001
Duan et al. (2018) [33] Retrospective cohort study Quality appraisal: good quality	Tamsulosin vs. no BPH medication	Incidence of dementia/1,000 person-years	HR: 1.17 (1.14–1.21)	31.3 vs. 25.9	< 0.001
	Tamsulosin vs. no BPH medication	Incidence of dementia (number of cases/number of patients)	RR: 0.96 (0.93–0.99) ^a^	9.442/161.729 vs. 9.847/161.729	< 0.05
	Tamsulosin vs. doxazosin	Incidence of dementia/1,000 person-years	HR: 1.20 (1.12–1.28)	32.7 vs. 27.5	< 0.001
	Tamsulosin vs. terazosin	Incidence of dementia/1,000 person-years	HR: 1.11 (1.04–1.19)	37.1 vs. 32.7	0.002
	Tamsulosin vs. alfuzosin	Incidence of dementia/1,000 person-years	HR: 1.12 (1.03–1.22)	30.4 vs. 28.4	0.010
	Tamsulosin vs. dutasteride	Incidence of dementia/1,000 person-years	HR: 1.26 (1.19–1.34)	32.7 vs. 26.5	< 0.001
	Tamsulosin vs. finasteride	Incidence of dementia/1,000 person-years	HR: 1.13 (1.07–1.19)	36.9 vs. 32.8	< 0.001
Gotoh et al. (2005) [35] RCT RoB rating: high risk	Tamsulosin 0.2 mg vs. naftopidil 50 mg	Change in IPSS pre vs. post administration		-8.4 (-10, -6.8) vs -5.9 (-7.3, -4.5)	0.060
	Tamsulosin 0.2 mg vs. naftopidil 50 mg	Change in QoL-score pre vs. post administration		-1.4 (-1.7, -1.1) vs -1.3 (-1.7, -1.0)	0.801
	Tamsulosin 0.2 mg vs. naftopidil 50 mg	ADEs		9/95 (9.5%) vs 9/90 (10%)	0.94
Hall and McMahon (2007) [52] Case–control study Quality appraisal: good quality	Fractures vs. no fractures total	Current doxazosin use	OR: 0.82 (0.63–1.08) Adj. OR: 0.90 (0.68–1.19)	66 (1.01%) vs 311 (1.17%)	> 0.05
	Fractures vs. no fractures	Current exposure to alpha-1 antagonist other than doxazosin	OR: 0.89 (0.71–1.12) Adj. OR: 0.93 (0.73–1.18)	94 (1.44%) vs. 446 (1.68%)	> 0.05
Hiremath et al. (2019) [54] Retrospective cohort study Quality appraisal: good quality	α1-blocker vs. other BP-lowering drugs	Incidence of hypotension related events	HR: 1.10 (1.01–1.20)	1,214 vs. 1,025	
	α1-blocker vs. other BP-lowering drugs	Incidence of falls	HR: 1.02 (0.92 – 1.13)		> 0.05
	α1-blocker vs. other BP-lowering drugs	Incidence of fractures	HR: 0.94 (0.82 – 1.08)		> 0.05
	α1-blocker vs. other BP-lowering drugs	Adverse cardiac event	HR: 1.06 (0.99–1.13)	2,251 vs. 1,914	
	α1-blocker vs. other BP-lowering drugs	All-cause mortality	HR: 1.06 (0.95–1.20)	681 vs. 545	
Hundemer et al. (2021) [32] Retrospective cohort study Quality appraisal: good quality	α1-blocker vs. other BP-lowering drugs	≥ 30% eGFR decline/1,000 person-years	HR: 1.14 (1.08–1.21)	3,036 (12.1%) vs. 2,548 (10.7%)	< 0.001
	α1-blocker vs. other BP-lowering drugs	Dialysis or kidneyTx/1,000 person-years	HR: 1.26 (1.13–1.44)	642 (1.52%) vs. 475 (1.14%)	< 0.001
	α1-blocker vs. other BP-lowering drugs	Cardiac events/1,000 person-years	HR: 0.92 (0.89–0.95)	6,595 (20.7%) vs. 6,774 (22.4%)	< 0.001
	α1-blocker vs. other BP-lowering drugs	Deaths/1,000 person-years	HR: 0.89 (0.84–0.94)	2,610 (6.07%) vs. 2,854 (6,76%)	< 0.001
	α1-blocker vs. other BP-lowering drugs	Hypotension/1,000 person-years	HR: 1.08 (0.96–1.21)	647 (1.53%) vs. 593 (1.43%)	> 0.05
	α1-blocker vs. other BP-lowering drugs	Syncope/1,000 person-years	HR: 1.23 (1.11–1.37)	816 (1.95%) vs. 656 (1.59%)	< 0.001
	α1-blocker vs. other BP-lowering drugs	Falls/1,000 person-years	HR: 1.00 (0.94–1.06)	2,388 (6.00%) vs. 2,376 (6.07%)	> 0.05
	α1-blocker vs. other BP-lowering drugs	Fractures/1,000 person-years	HR: 1.03 (0.95–1.12)	1,156 (2.79%) vs. 1,111 (2.72%)	> 0.05
Lowe (1994) [58] Meta-analysis based on 6 double-blind RCTs RoB rating: high risk	Terazosin vs. placebo in the age group 65 y – 74 y	Postural symptoms		15/235 (6.0%) vs. 2/143 (1.4%)	< 0.05
	Terazosin vs. placebo in the age group 65 y – 74 y	Impotence		4/235 (1.7%) vs. 1/143 (0.7%)	> 0.05
	Terazosin vs. placebo in the age group 65 y – 74 y	Syncope		3/235 (1.3%) vs. 0/143 (0.0%)	> 0.05
	Terazosin vs. placebo in the age group > 74 y	Postural symptoms		2/50 (4.0%) vs. 0/19 (0.0%)	> 0.05
	Terazosin vs. placebo in the age group > 74 y	Impotence		0/50 (0.0%) vs. 0/19 (0.0%)	> 0.05
	Terazosin vs. placebo in the age group > 74 y	Syncope		0/50 (0.0%) vs. 0/19 (0.0%)	> 0.05
Nishino et al. (2006) [36] Randomized crossover trial RoB rating: some concerns	Tamsulosin 0.2 mg vs. naftopidil 50 mg	Change in IPSS pre- to post- administration between substances		20.4 (3.5) to 9.3 (3.0) vs. 20.4 (3.5) to 8.9 (3.2)	0.265
	Tamsulosin 0.2 mg vs. naftopidil 50 mg	Change in QoL-score pre- to post- administration between substances		4.9 (0.7) to 2.7 (1.1) vs.4.9 (0.7) to 2.6 (1.1)	0.201
	Naftopidil 50 mg vs. tamsulosin 0.2 mg	ADEs		0/34 (0%) vs. 0/34 (0%)	> 0.05
	Tamsulosin 0.2 mg vs. naftopidil 50 mg	Withdrawals		0/34 (0%) vs. 0/34 (0%)	> 0.05
Oelke et al. (2014) [48] Ranodmized double-blind placebo-controlled trial RoB rating: high risk	Tamsulosin 0.4 mg vs. placebo	TSS-BPH ≤ 65 y		28.8 (16.9) vs. 31.2 (17.3)	0.212
	Tamsulosin 0.4 mg vs. placebo	TSS-BPH > 65 y		32.4 (15.8) vs. 32.2 (17.9)	0.759
	Tadalafil 5 mg vs. placebo	TSS-BPH ≤ 65 y		25.2 (17.8) vs. 31.2 (17.3)	0.013
	Tadalafil 5 mg vs. placebo	TSS-BPH > 65 y		29.0 (17.6) vs. 32.2 (17.9)	0.184
Roehrborn (2006) [49] Randomized double-blind placebo-controlled trial RoB rating: high risk	Alfuzosin 10 mg vs. placebo	Worsening of IPSS by ≥ 4 within 2 years treatment ≥ 65 y	RR: 0.84 (0.62–1.15) ^a^	62/443 (14%) vs. 72/433 (16.6%)	> 0.05
	Alfuzosin 10 mg vs. placebo	Occurrence of AUR within 2 years treatment ≥ 65 y	RR: 0.98 (0.39–2.44) ^a^	9/443 (2%) vs. 9/433 (2,1%)	> 0.05
	Alfuzosin 10 mg vs. placebo	Need for BPH-related surgery within 2 years treatment ≥ 65 y	RR: 0.64 (0.36–1.12) ^a^	19/443 (4.3%) vs. 29/433 (6.7%)	> 0.05
Siemens et al. (2021) [55] Retrospective cohort study Quality appraisal: low quality	α1-blocker use vs. no medication	Incidence of new cardiac failure	HR: 1.22 (1.18–1.26)		< 0.001
	α1-blocker + 5-ARI combination vs. no medication	Incidence of new cardiac failure	HR: 1.16 (1.12–1.21)		< 0.001
	Non-selective α1-blocker vs. selective α1-blocker	Incidence of new cardiac failure	HR: 1.08 (1.00–1.17)		0.04
Tae et al. (2019) [56] Retrospective cohort study Quality appraisal: good quality	Tamsulosin vs. no medication	Incidence of dementia	HR: 0.705 (0.635–0.782)	681 (20.4%) vs. 754 (22.6%)	< 0.001
	Doxazosin vs. no medication	Incidence of dementia	HR: 0.710 (0.637–0.792)	624 (21.1%) vs. 689 (23.3%)	< 0.001
	Terazosin vs. no medication	Incidence of dementia	HR: 0.831 (0.749–0.921)	708 (22.5%) vs. 742 (23.6%)	0.001
	Alfuzosin vs. no medication	Incidence of dementia	HR: 0.682 (0.607–0.766)	529 (19.2%) vs. 623 (22.6%)	< 0.001
	Medium dose level doxazosin vs. tamsulosin	Incidence of dementia	HR: 1.010 (0.906–1.126)		0.859
	Medium dose level terazosin vs. tamsulosin	Incidence of dementia	HR: 1.085 (0.949–1.240)		0.233
	Medium dose level alfuzosin vs. tamsulosin	Incidence of dementia	HR: 1.122 (0.950–1.324)		0.176
Testa et al. (2018) [53] Case–control study Quality appraisal: good quality	Syncope due to orthostatic hypotension (OH) vs. non-OH syncope	α1-blocker use	RR: 1.67 (1.00–2.85) Adj. for age and sex: RR: 1.48 (0.84–2.60)	28/170 (16.5%) vs 18/184 (9.8%)	0.043
	Syncope due to orthostatic hypotension (OH) vs. non-OH syncope	α1-blocker + diuretic	RR: 1.70 (1.04–2.78) Adj. for age and sex: RR: 1.83 (0.85–3.96)	14/170 (8.2%) vs 6/184 (3.3%)	0.036
Welk et al. (2015) [51] Retrospective cohort study Quality appraisal: good quality	α1-blocker use vs. no use	Falls	OR: 1.14 (1.07–1.21)	2,129 (1.45%) vs. 1,881 (1.28%)	< 0.05
	α1-blocker use vs. no use	Fracture	OR: 1.16 (1.04–1.29)	699 (0.48%) vs 605 (0.41%)	< 0.05
	Tamsulosin use vs. no use	Falls	OR: 1.12 (1.04–1.19)	1,810 (1.47%) vs. 1,625 (1.32%)	< 0.05
	Alfuzosin use vs. no use	Falls	OR: 1.24 (1.04–1.48)	279 (1.34%) vs. 226 (1.09%)	< 0.05
	Silodosin use vs. no use	Falls	OR: 1.35 (0.83–2.18)	40 (1.47%) vs. 30 (1.10%)	> 0.05
Yokoyama et al. (2011) [34] RCT RoB raiting: high risk	Silodosin 8 mg vs. tamsulosin 0.2 mg vs. naftopidil 50 mg	Change in IPSS pre- vs. post- administration between substances		18.7 (0.7) to 13.8 (1.2) vs. 18.0 (1.1) to 10.7 (1.4) vs. 17.4 (0.8) to 11.3 (1.1)	> 0.05
	Silodosin 8 mg vs. tamsulosin 0.2 mg vs. naftopidil 50 mg	Change in QoL-score pre- to post- administration between substances		4.5 (0.1) to 3.4 (0.2) vs. 4.5 (0.1) to 2.7 (0.3) vs. 4.5 (0.1) to 3.1 (0.2)	> 0.05
	Silodosin 8 mg vs. tamsulosin 0.2 mg vs. naftopidil	Withdrawals due to ADEs		4/41 (9,8%) vs. 1/39 (2,6%) vs. 1/42 (2,4%)	
	Silodosin 8 mg vs. tamsulosin 0.2 mg vs. naftopidil	Abnormal ejaculation after 4-week treatment		10/11 (90,9%) vs. 1/12 (8,3%) vs. 1/15 (6,7%)	

^a^ Results calculated based on the figures provided in the original paper

^b^ Decision taken by the investigator: possibly or probably drug related

^c^ Includes dizziness, headache, tachycardia, palpitation, postural hypotension and syncope

Abbreviations: *5-ARI* 5-alpha reductase inhibitor, *ADE* adverse drug event, *AUR* acute urinary retention, *BPH* benign prostatic hyperplasia, *CVD* cardiovascular disease, *IPSS* international prostate symptom score, *QoL* quality of life, *RCT* randomized controlled trial, *RoB* risk of bias

#### Cardiovascular events

Results from the ALLHAT study (2003) showed an increased risk of doxazosin compared to chlorthalidone for combined cardiovascular events (i.e. death from coronary heart disease, nonfatal myocardial infarction, stroke, coronary revascularisation, hospitalised or treated angina, treated or hospitalised congestive heart failure, and peripheral artery disease and heart failure). The combined cardiovascular risk was elevated among patients < 65 years (relative risk RR 1.15, 95% confidence interval CI 1.04 – 1.27) and more pronounced for patients ≥ 65 years (RR 1.23, 95% CI 1.14 – 1.32). The results for heart failure for participants < 65 years of age (RR 1.76, 95% CI 1.40 – 2.22) and participants ≥ 65 years (RR 1.89, 95% CI 1.65 – 2.17) are even clearer [47]. Siemens et al. (2021) analysed the incidence of heart failure in patients with BPH and found an elevated risk in patients treated with alpha-1 antagonists vs. no medication (HR 1.22, 95% CI 1.18 – 1.26) with a slightly higher risk with non-selective vs. selective alpha-1 antagonists (HR 1.08, 95% CI 1.00 – 1.17) [55]. In contrast to above stated findings, Hundemer et al. (2021) showed a decrease in cardiac events (composite of MI, coronary revascularisation, congestive heart failure or atrial fibrillation) when comparing alpha-1 antagonist treatment with other BP lowering drug treatment regimes (HR 0.92, 95% CI 0.89 – 0.95) [32] whereas Hiremath et al. (2019) demonstrated a slight but insignificant increase focusing on women only (HR 1.06, 95% CI 0.99 – 1.13) [54].

#### Efficacy in reducing BPH related symptoms

Significant improvement of IPSS for tamsulosin in a pre-post comparison was demonstrated by Gotoh et al. (2005) (mean reduction -8.4, 95% CI -10 – (-6.8); *p* < 0.001) [35], Nishino et al. (2005) (mean score [standard deviation (SD)] 20.4 [3.5] vs. 9.3 [3.0]; *p* < 0.001) [36] and Yokoyama et al. (2011) (mean score [SD] 18.0 [1.1] vs. 10.7 [1.4]; *p* < 0.001) [34]. Similar results were reported for naftopidil in the same studies. Silodosin was only investigated in one study conducted by Yokoyama et al. (2011) with comparable results [34]. Significance in superiority of one agent over another could not be demonstrated with intergroup p-values at 0.060 [35], 0.265 [36] and > 0.05 [34].

**Fig. 2:**
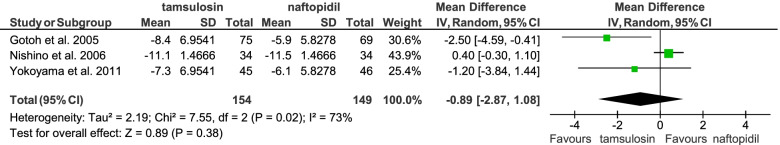
Meta-analysis on the change in IPSS tamsulosin 0.2 mg vs. naftopidil 50 mg pre to post administration

**Fig. 3:**
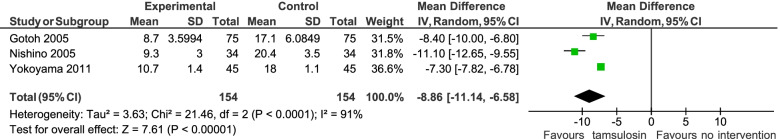
Meta-analysis of the change in IPSS: tamsulosin 0.2 mg pre to post administration

A meta-analysis was performed to combine the results of these three studies including a total of 303 study participants and assessing the efficacy of tamsulosin and naftopidil in reducing BPH related urinary symptoms expressed as change of mean IPSS pre to post intervention [34–36]. The result shows non-superiority of one drug over the other (mean difference -0.89, 95% CI -2.87 – 1.08; see Fig. 2). A second meta-analysis consolidated the results of the same three studies for tamsulosin 0.2 mg (total *n* = 154), comparing results pre vs. post administration. Tamsulosin was chosen due to its importance on the European market as compared with naftopidil. The result favours the intervention with tamsulosin 0.2 mg (post administration) over no intervention (pre administration) showing a significant reduction in urinary symptoms (mean difference -8.86, 95% CI -11.14 – -6.58; see Fig. 3).

#### Progression events from BPH associated LUTS

In a two-year follow up, the results of Roehrborn (2006) showed that alfuzosin has a non-significant positive effect on slowing down the progression of the IPSS (RR 0.84, 95% CI 0.62 – 1.15) and the need for BPH related surgery (RR 0.64, 95% CI 0.36 – 1.12) and no effect on the reduction of acute urinary retention events (RR 0.98, 95% CI 0.39 – 2.44) in patients over the age of 65 years [49].

#### Treatment satisfaction and QoL

The results of Oelke et al. (2014) showed no significantly better scores in treatment satisfaction of patients > 65 years with LUTS suggestive of BPH when treated with tamsulosin than with placebo (mean score [SD] 32.4 [15.8] vs. 32.2 [17.9]; *p*-value = 0.759) [48].

In a pre-post comparison, significant improvement in QoL was demonstrated for tamsulosin, naftopidil and silodosin in three trials [34–36]. Significant differences in the improvement of QoL-scores between tamsulosin, naftopidil and silodosin could not be demonstrated with intergroup p-values at 0.801 [35], 0.201 [36] and > 0.05 [34].

**Fig. 4:**
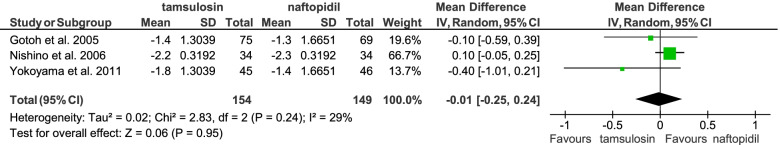
Meta-analysis on the change in QoL-score tamsulosin 0.2 mg vs. naftopidil 50 mg pre to post administration

**Fig. 5:**
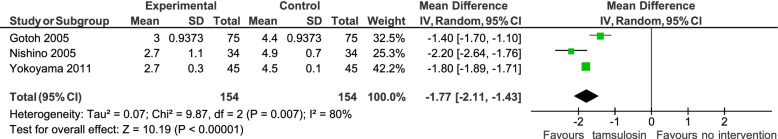
Meta-analysis of the change in QoL-Score tamsulosin 0.2 mg pre to post administration

A meta-analysis was performed to combine the results of three interventional studies including a total of 303 study participants assessing the efficacy of tamsulosin and naftopidil in improving QoL expressed as reduction of QoL-score pre to post administration [34–36]. The result shows non-superiority of one drug over the other (mean difference -0.01, 95% CI -0.25 – 0.24; see Fig. 4). A second meta-analysis consolidated the results of the same three studies for tamsulosin 0.2 mg only (total *n* = 154), comparing results pre vs. post administration. The result favours the intervention with tamsulosin 0.2 mg (post administration) over no intervention (pre administration) showing a significant improvement in QoL of patients with LUTS (mean difference -1.77, 95% CI -2.11 – -1.43; see Fig. 5).

#### ADEs

Chapple et al. (1997) conducted a safety analysis for tamsulosin compared with placebo in 291 older patients and found no significant difference in the occurrence of any adverse events between groups (tamsulosin: 70/191, 37%; placebo: 31/100, 31%; *p* = 0.330) or adverse events, which were considered drug-related (tamsulosin: 23/191, 12%; placebo: 9/100, 9%; *p* = 0.459) or adverse events possibly associated with vasodilation (tamsulosin: 8/191, 4.2%; placebo: 4/100, 4%; *p* = 0.523) [59]. Buzelin et al. (1997) reported an almost equal distribution of ADEs comparing alfuzosin with placebo (alfuzosin: 12/149, 8.1%; placebo: 12/153, 7.8%). Also, adverse events related to vasodilation (alfuzosin: 2/149, 1.3%; placebo: 2/153, 1.3%) occurred evenly between both groups [57]. Lowe et al. (1994) provided a more detailed analysis on different specific ADEs (see Additional file 5) [58].Three interventional studies reported on ADEs and found no significant differences between agents [34–36].

**Fig. 6:**
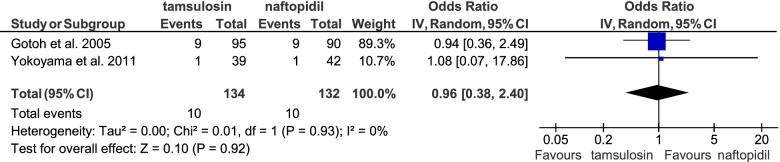
Meta-analysis on the occurrence of ADEs while treatment with tamsulosin 0.2 mg or naftopidil 50 mg

A meta-analysis was undertaken to combine the results of two interventional studies (total *n* = 266) assessing the occurrence of ADEs for treatment with tamsulosin or naftopidil [34, 35]. The result shows no significant differences between these two agents (odds ratio 0.96, 95% CI 0.38 – 2.40; see Fig. 6).

#### Syncope and hypotension

Testa et al. (2018) presented results indicative of the fact that alpha-1 antagonist use may play a role in orthostatic hypotension related syncopes in adults with dementia especially when taken concomitantly with diuretics (adjusted for age and sex RR 1.83, 95% CI 0.85 – 3.96) [53].

The effect of treatment initiation with alpha-1 antagonists on hypotension and hypotension related adverse events was examined by four observational studies. The numbers reported by Chrischilles et al. (2001) showed a significant difference between the alpha-1 antagonist cohort and no alpha-1 antagonist cohort when comparing incidence rates of hypotension related ADEs four months before treatment initiation with the four months after (*p*-value = 0.001) [50]. Welk et al. (2015) demonstrated an increased risk for hospitalisation or emergency room assessment due to hypotension (Odds ratio [OR] 1.80, 95% CI 1.59 – 2.03) within 90 days after treatment initiation with alpha-1 antagonists tamsulosin, alfuzosin or silodosin [51]. Hiremath et al. (2019) also showed an association of alpha-1 antagonist treatment and the incidence of hypotension related events when compared to other BP-lowering medication (HR 1.10, 95% CI 1.01 – 1.20) [54], whereas Hundemer et al. (2021) reported non-significant results in hypotension/1,000 person-years (HR 1.08, 95% CI 0.96 – 1.21) [32].

#### Falls and fractures

Welk et al. (2015) demonstrated a correlation of initiation of alpha-1 antagonist treatment and new fracture (OR 1.16, 95% CI 1.07 – 1.21), head trauma (OR 1.15, 95% CI 1.04 – 1.27) and falls (OR 1.14, 95% CI 1.07 – 1.21). Agent specific results for falls differed between tamsulosin, alfuzosin and silodosin [51]. In contrast, Hall and McMahon (2007) could not show an association between fractures commonly due to falls (i.e. hip/femur, wrist and humerus) and initiation of doxazosin use (OR 0.57, 95% CI 0.17 – 1.92), current doxazosin use (adjusted OR 0.90, 95% CI 0.68 – 1.19) or any previous doxazosin use (adjusted OR 0.92, 95% CI 0.69 – 1.23) [52]. Hiremath et al. (2019) also did not find a significant association with the occurrence of falls (HR 1.02, 95% CI 0.92 – 1.13) or fractures (HR 0.94, 95% CI 0.82 – 1.08) when comparing alpha-1 antagonist treatment vs. other BP-lowering drugs [54]. Similar results were presented by Hundemer et al. (2021): falls/1,000 person-years (HR 1.00, 95% CI 0.94 – 1.06) and fractures/1,000 person-years (HR 1.03, 95% CI 0.95 – 1.12).

**Fig. 7:**
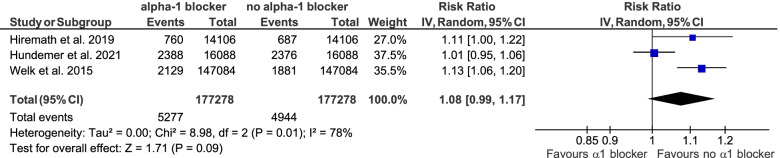
Meta-analysis on the association of initiation of alpha-1 antagonist treatment and the incidence of falls

**Fig. 8:**
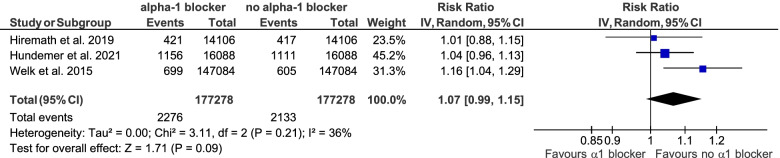
Meta-analysis on the association of initiation of alpha-1 antagonist treatment and the incidence of fractures

Meta-analyses were performed to synthesize the data presented above based on three retrospective cohort trials [32, 51, 54]. No significant association of initiation of alpha-1 antagonist treatment with falls (RR 1.08, 95% CI 0.99 – 1.17) or fractures (RR 1.07, 95% CI 0.99 – 1.15) could be demonstrated. The results of these meta-analyses are depicted in Figs. 7 and 8.

#### Ejaculation disorders

Participants receiving silodosin showed a high percentage of ejaculation disorders after 4 weeks of treatment (10/11, 90,9%) and after 12 weeks of treatment (8/10, 80%) whereas tamsulosin (4 weeks: 1/12, 8,3%; 12 weeks: 1/5, 20%) and naftopidil (4 weeks: 1/15, 6,7%; 12 weeks: 1/14, 7,1%) did not [34]. Chapple et al. (1997) reported on elevated numbers of abnormal ejaculations during treatment with tamsulosin compared to placebo (tamsulosin: 5/191, 2.6%; placebo: 1/100, 1%; *p* = 0.668) [59].

#### Dementia

Duan et al. (2018) found significant increases in the incidence (number of cases/1.000 person-years) of dementia when tamsulosin was compared to no BPH medication (hazard ratio (HR) 1.17, 95% CI 1.14 – 1.21), to doxazosin (HR 1.20, 95% CI 1.12 – 1.28), to terazosin (HR 1.11, 95% CI 1.04 – 1.19), and to alfuzosin (HR 1.12, 95% CI 1.03 – 1.22) as well as dutasteride (HR 1.26, 95% CI 1.19 – 1.34) and finasteride (HR 1.13, 95% CI 1.07 – 1.19) [33]. Tae et al. (2019) could not reproduce these findings and published results showing a decreased risk of dementia (number of cases/number of patients) when comparing tamsulosin to no BPH medication (HR 0.705, 95% CI 0.635 – 0.782). Similar results were presented for doxazosin (HR 0.710, 95% CI 0.637 – 0.792), terazosin (HR 0.831, 95% CI 0.749 – 0.921) and alfuzosin (HR 0.682, 95% CI 0.607 – 0.766). There was no significant difference between substances when comparing medium dose levels [56].

**Fig. 9:**
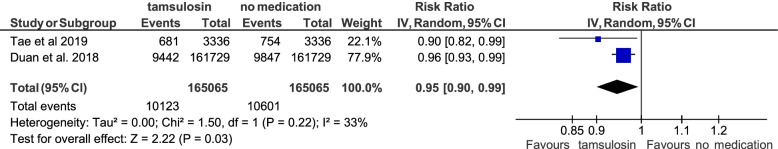
Meta-analysis on the association of tamsulosin treatment vs. no medication and the incidence of dementia

A meta-analysis was performed to synthesize the results (number of cases/number of patients) of these two studies showing a significantly lower incidence of dementia in patients treated with tamsulosin vs. no medication (RR 0.95, 95% CI 0.90 – 0.99). The results of this meta-analysis are shown in Fig. 9.

### Quality appraisal of included studies

#### Meta-analyses

None of the included meta-analyses [57–59] met any of the criteria defined in the AMSTAR 2 assessment tool [41] with the exception of statement of funding. Neither of the publications provide detailed information on the methodology used. All meta-analyses used unpublished information and presumably based their analysis on raw data. Due to a lack of transparency on the calculations performed the figures published cannot be reconstructed neither is it possible to assess homogeneity of the data or likelihood of publication bias of included studies. A comprehensible reproduction of the results is therefore not possible. See Table 3 for details on quality appraisal.

**Table 3:** Critical quality appraisal for included meta-analyses according to AMSTAR 2 [41]

**Authors (Year)**	PICO scheme used?	Review methods prior to conduct?	Explanation for inclusion of study designs?	Comprehensive literature search?	≥ 2 independent reviewers performed study selection?	≥ 2 independent reviewers performed data extraction?	List of excl. studies?	Detailed prescription of incl. studies?	RoB assessment?	Sources of funding of incl. studies mentioned?	Appropriate methods for meta-analysis?	Influence of RoB in meta-analysis?	RoB considered in interpretation/discussion of results?	Explanation for heterogeneity?	Publication bias considered?	Funding and conflict of interest stated?
Buzelin et al. (1997) [57]	N	N	N	N	N	N	N	N	N	N	U	N	N	N	N	N
Lowe (1994) [58]	N	N	N	N	N	N	N	N	N	N	U	N	N	N	N	Y
Chapple et al. (1997) [59]	N	N	N	N	N	N	N	N	N	N	U	N	N	N	N	Y

*Y* Yes, *N* No, *U* Unclear, *RoB* Risk of Bias

#### Interventional studies

The result of the assessment for risk of bias based on the appraisal of five different categories according to the RoB 2 tool [38] is shown for each study in Table 4.

**Table 4:** Critical quality appraisal for included interventional studies according to the Cochrane Collaboration Tool [38]

Authors (Year)	Study Type	Selection Bias	Performance Bias	Attrition Bias	Detection Bias	Reporting Bias	Overall risk-of-bias judgement
		**Randomization and concealment**	**Concealment of intervention/outcome**	**Missing outcome data**	**Measurement of outcome**	**Selective reporting**	
ALLHAT (2003) [47]	Randomised double-blind controlled trial	LR	LR	LR	LR	LR	LR
Gotoh et al. (2005) [35]	Randomised controlled trial	SC	LR	HR	SC	SC	HR
Nishino et al. (2006) [36]	Randomised crossover trial	LR	LR	LR	SC	SC	SC
Oelke et al. (2014) [48]	Randomised double-blind placebo-controlled trial	LR	LR	HR	LR	LR	HR
Roehrborn (2006) [49]	Randomised double-blind placebo-controlled trial	LR	LR	HR	LR	LR	HR
Yokoyama et al. (2011) [34]	Randomised controlled trial	LR	LR	HR	SC	SC	HR

*LR* low risk of bias, *HR* high risk of bias, *SC* some concerns

The overall risk-of-bias judgement was “low risk” for one study [47], one study was rated with “some concerns” [36] and four trials were graded “high risk” [34, 35, 48, 49]. Selection bias arising from randomization of the patients raised some concerns in the trial by Gotoh et al. (2005) as baseline characteristics in the categories total IPSS (*p*-value = 0.088) and prostate volume (*p*-value = 0,06) were imbalanced between the two interventional groups [35]. Four studies were classified as “high risk” for attrition bias due to a high share of dropouts ranging from 11,2% [48] to 33,7% [49], either in large part attributable to the intervention [34, 48, 49] or without delivering comprehensible data [35]. Some concerns were raised for three trials [34–36] due to missing information on blinding of participants and potential influence on the self-assessment in the IPSS. The same studies were also downgraded to some concerns for reporting bias as none of them provided a study protocol. It must be considered that the doxazosin component of the ALLHAT study was stopped prematurely due to higher rates of heart failure and combined cardiovascular events [47].

#### Observational studies

Nine observational studies were included and their risk of bias assessed in accordance to their study type, three retrospective cohort studies [32, 33, 50, 51, 54–56] and two case–control studies [52, 53]. Except for two [50, 55] all studies were well rated on the majority of CASP items and can therefore be considered good quality. For details, refer to Tables 5 and 6.

**Table 5:** Critical quality appraisal for included cohort studies according to the Critical Appraisal Skills Programme (CASP) [40]

**Authors (Year)**	**Cohort studies**	Focused issue?	Acceptable recruitment of cohorts?	Accurate exposure measurement?	Accurate outcome measurement?	Relevant confounders identified?	Confounders considered in design/analysis?	Follow-ups complete?	Follow-up period long enough?	(Precision of) Results of study? ^*^	Believe in results?	Results applicable?	Results fit evidence?
Chrischilles et al. (2001) [50]	Retrospective cohort study	Y	Y	U	N	N	N	Y	Y		N	N	U
Duan et al. (2018) [33]	Retrospective cohort study	Y	Y	Y	Y	Y	Y	Y	U		Y	Y	U
Hiremath et al. (2019) [54]	Retrospective cohort study	Y	Y	Y	Y	Y	Y	Y	Y		Y	Y	U
Hundemar et al. (2021) [32]	Retrospective cohort study	Y	Y	Y	Y	Y	Y	Y	Y		Y	Y	U
Siemens et al. (2021) [55]	Retrospective cohort study	Y	Y	Y	Y	N	N	Y	Y		U	U	U
Tae et al. (2019) [56]	Retrospective cohort study	Y	Y	Y	Y	Y	Y	Y	Y		Y	Y	U
Welk et al. (2015) [51]	Retrospective cohort study	Y	Y	Y	Y	Y	Y	Y	Y		Y	Y	U

*Y* Yes, *N* No, *U* Unclear

^*^ Results are to be found in Table 2

**Table 6:** Critical quality appraisal for included case–control studies according to the Critical Appraisal Skills Programme (CASP) [39]

Authors (Year)	Case–control studies	Focused issue?	Appropriate method?	Acceptable recruitment of cases?	Acceptable controls?	Accurate exposure measurement?	Groups treated equally?	Confounders considered in design/analysis?	Results of study? ^*^	Believe in results?	Results applicable?	Results fit evidence?
Hall and McMahon (2007) [52]	Case–control study	Y	Y	Y	Y	Y	Y	Y		Y	Y	U
Testa et al. (2018) [53]	Case–control study	Y	Y	Y	Y	Y	Y	Y		Y	U	U

*Y* Yes, *N* No, *U* Unclear

^*^ Results are to be found in Table 2

### Sponsoring and conflict of interest of included studies

Fifteen of eighteen included studies reported about conflict of interest and study sponsors [32, 33, 36, 47–56, 58, 59], seven of which reported direct or indirect funding by pharmaceutical companies [47–50, 52, 58, 59]. Six studies reported support from national institutes [32, 33, 47, 51, 54, 55], two were university funded [36, 56] and one study was endorsed by a local society [53]. Three studies did not give any information on potential sources of conflict of interest [34, 35, 57].

### Additional references of interest for the development of recommendations

Two evidence based guidelines [42, 43], two Cochrane reviews [66, 67], one network meta-analysis [68] and four systematic reviews [69–72], three of them including a meta-analysis, were identified and incorporated into the recommendations in addition to four randomised controlled trials included in this systematic review [34–36, 47]. All the additional literature was not included in the systematic review due to their missing focus on the age subgroup of people ≥ 65 years but was deemed relevant as additional information was retrieved concerning efficacy and risk profile of alpha-1 antagonists.

Nickel et al. (2008) reported about significant improvements in IPSS for alfuzosin, terazosin, doxazosin and tamsulosin with no statistically significant differences between substances [71]. Similar findings were reported by Djavan et al. (2004) [72] and Yuan et al. (2015) [68]. Fusco performed two meta-analyses in 2016 and 2018 indirectly confirming these results as significant improvements in the Bladder Outlet Obstruction Index (BOOI) could be shown for alfuzosin, terazosin, doxazosin, tamsulosin, naftopidil and silodosin [69, 70]. Jung et al. (2017) conducted a Cochrane review on silodosin and reported significant improvement in IPSS and QoL versus placebo but no substantial differences when compared to tamsulosin or alfuzosin [66]. Hwang et al. (2018) focused on naftopidil in their Cochrane review with no significant differences in IPSS and QoL when compared with tamsulosin or silodosin [67]. Regarding drug safety, two main areas of interest were covered by the additional references being vasodilation related ADEs (e.g. dizziness, hypotension, syncope) and sexual ADEs. A significant increase in vasodilation related ADEs was reported by Nickel et al. (2008) for alfuzosin, terazosin and doxazosin, a clear tendency but not significant (p = 0.053) for tamsulosin [71]. Similar results have been mentioned by Djavan et al. (2004) [72]. Yuan et al. (2015) demonstrated significantly increased total ADEs for doxazosin, terazosin and silodosin and a tendency, but insignificant, towards increased severe adverse events for doxazosin and terazosin [68]. Silodosin significantly increased rates of sexual adverse events in all comparisons [66]. Treatment with naftopidil compared with tamsulosin and silodosin showed no difference in cardiovascular risk profile or withdrawal rates but significantly less sexual adverse events than for silodosin [67].

### Recommendations

Two recommendations were developed based on the findings of this systematic review and additional references of interest as stated above (see Table 7 for recommendations). One recommendation is related to the management of bothersome LUTS suggestive of BPH. Alpha-1 antagonists prove to be effective in the reduction of the IPSS, an internationally used and validated score to measure urinary symptoms, while having an acceptable risk profile. The ADEs differ between agents and therefore have to be considered on a patient-oriented basis. The recommendation was based on three randomised controlled trials included in this systematic review [34–36], the most recent version of the guideline for the management of non-neurogenic male LUTS by the EAU [43], two Cochrane reviews [66, 67], and four systematic reviews including meta-analysis [69–72]. The recommendation was given the following ratings: strong recommendation and low in quality. The quality was downgraded from high to low due to study limitations in the randomised controlled trials and indirectness of additional references. Based on the findings of the ALLHAT study [47] and the 2018 ESC/ESH guidelines for the management of arterial hypertension [42] the second recommendation is to replace doxazosin with another antihypertensive drug in the treatment of hypertension. The recommendation was rated as strong recommendation and of high quality.

**Table 7:** Recommendations for the use of alpha-1 antagonists in older people with LUTS suggestive of BPH or arterial hypertension

Recommendation	Strength of recommendation	Quality of evidence	Type of evidence
Alpha-1 antagonists prove to be effective in the reduction of urinary symptoms (IPSS) in the treatment of bothersome LUTS suggestive of BPH irrespective of the patient’s age. Particularities of different agents’ risk profiles especially regarding hypotension related and sexual adverse events are to be considered on a patient-oriented basis	Strong (Benefits outweigh the undesirable effects and good results on the improvement of QoL)	Low (Downgraded for study limitations in the RCTs and indirectness as only the three RCTs focused on patients ≥ 65 years and two SRs did not define the IPSS as outcome)	• 1 guideline by European Association of Urology [43] • 2 Cochrane reviews [66, 67] 4 systematic reviews (SRs) incl. three meta-analyses [69–72] 1 network meta-analysis [68] • 3 randomised controlled trials (RCTs) [34–36]
It is recommended to replace doxazosin for the treatment of arterial hypertension as it is likely to be less effective than other antihypertensive drugs in reducing combined CVD, and heart failure in particular, unless there is no other suitable option (e.g. resistant hypertension if intolerant to spironolactone)	Strong (High quality evidence on clinically highly relevant outcomes)	High (Low risk of bias)	• 1 guideline by European Society of Cardiology and European Society of Hypertension [42] • 1 randomised controlled trial [47]

Combined CVD = fatal coronary heart disease, nonfatal myocardial infarction, stroke, coronary revascularization procedures, hospitalised or treated angina, treated or hospitalised congestive heart failure and peripheral artery disease

## Discussion

This systematic review was performed to summarize the current body of knowledge about the efficacy/effectiveness as well as the safety profile of alpha-1 antagonists in the management of arterial hypertension and LUTS suggestive of BPH in patients ≥ 65 years and to derive recommendations on the use of alpha-1 antagonists in the subgroup of older adults.

### Summary of main results

Eighteen studies were included in this systematic review: three meta-analyses, six randomised controlled trials, seven cohort studies and two case–control studies. The studies varied in terms of geographical focus, length of follow-up (shortest 4 weeks; longest 13 years), characteristics of participants, interventions, and outcomes. One study analysed the efficacy of doxazosin to reduce cardiovascular events in the management of arterial hypertension, two more looked at potential side-effects. The other trials studied alpha-1 antagonists in the management of LUTS suggestive of BPH. The included studies reported on cardiovascular outcomes, change in IPSS, QoL, treatment satisfaction, disease progression and typical ADEs such as vasodilatory adverse events (e.g. dizziness, syncope, falls, fractures) and sexual adverse events as well as dementia. Three studies reported on mortality. Based on the results of included studies it was possible to perform meta-analyses on six different outcomes: occurrence of ADEs, change in IPSS, change in QoL-score, and incidence of dementia, falls and fractures.

Doxazosin, used as an antihypertensive medication in older patients, seems to be inferior to chlorthalidone in reducing cardiovascular risk and especially heart failure. Alpha-1 antagonists seem to be effective in patients ≥ 65 years in reducing lower urinary tract symptoms due to BPH reflected by a substantial decrease of the IPSS and an increase in QoL. The study results point out certain ADEs but are inconclusive in defining a reliable risk profile for any of the alpha-1 antagonists. Alpha-1 antagonists may produce certain ADEs due to vasodilation and ejaculatory disorders with differences between substances. There is inconclusive data on the effect of alpha-1 antagonists on fractures, falls and the association of tamsulosin use and occurrence of dementia.

Two recommendations could be derived from the results for older adults. One concerning the role of doxazosin in the management of arterial hypertension and the other regarding the role of alpha-1 antagonists in the treatment of LUTS suggestive of BPH.

### Agreements and disagreements with other studies or reviews

To the best of our knowledge, the findings of the ALLHAT study have never been reproduced since. The Anglo-Scandinavian Cardiac Outcomes Trial (ASCOT) trial [73] examined the effect of doxazosin as third-line antihypertensive therapy in resistant hypertension and found no excess of heart failure over a median follow-up of 12 months. Wolak et al. (2014) found a significant increase in the composite of cardiac death and acute myocardial infarction in patients being treated with doxazosin for hypertension (as opposed to treatment for LUTS) with a moderate-to-severe ischemia on myocardial perfusion imaging. There was no focus on heart failure [74]. Neither of the studies published subgroup analyses for patients ≥ 65 years. Siemens et al. (2021) found an increased incidence of heart failure in patients treated with alpha-1 antagonists for LUTS due to BPH in their retrospective cohort study.

Three randomised controlled trials [34–36] approved of the efficacy of tamsulosin and other alpha-1 antagonists in the reduction of urinary symptoms in older adults and improvement of QoL, which had previously been shown in several trials without focus on the specific age subgroup [66–72]. As regards the agents’ risk profiles comparable results were reported for tamsulosin, silodosin and naftopidil with a higher occurrence of sexual adverse events for silodosin [34–36, 66, 67].

Nickel et al. (2008) show significantly increased vasodilation related adverse events (i.e. dizziness, hypotension, syncope) for alfuzosin, terazosin, doxazosin and almost significant for tamsulosin [71]. Similar results were calculated by Yuan et al. (2015) in their network meta-analysis [68]. This corresponds to the findings on a rise in hypotension related adverse events [50] and fractures and falls [51] upon treatment initiation with doxazosin/terazosin/prazosin and tamsulosin/silodosin/alfuzosin, respectively. These results, however, could not be reproduced by Hall and McMahon (2007), Hiremath et al. (2019) and Hundemer et al. (2021) and our meta-analyses [32, 52, 54]. As opposed to the results of most of the literature reporting significant improvement in symptom scores and QoL scores, Oelke et al. (2014) could not show considerably better treatment satisfaction scores in older adults for tamsulosin than for placebo [48].

### Applicability of results

Most findings of this systematic review confirm the important standing of alpha-1 antagnonists in the management of patients with LUTS suggestive of BPH and the minor role of doxazosin in the management of arterial hypertension. Nevertheless, some issues must be addressed concerning the applicability of the results.

The short follow-up time limits the the ability to appraise the effects in long-term treatment with alpha-1 antagonists in LUTS suggestive of BPH [34–36]. The applicability of the results is additionally impaired as the dose regime used for tamsulosin was 0.2 mg once daily in all three trials, which is lower than the recommended daily dose of tamsulosin 0.4 mg in Western countries. It also has to be considered that the trials were not placebo controlled. Whether alpha-1 antagonists can be recommended for long-term treatment remains doubtful as the results by Roehrborn et al. (2006) demonstrate similar progression event rates for treatment with alfuzosin as for placebo [49] and results about a possible relationship between tamsulosin and the incidence of dementia remain doubtful [33, 56]. Other classes of drugs such as 5alpha-reductase inhibitors (e.g. dutasteride, finasteride) or phosphodiesterase type 5 inhibitors (e.g. tadalafil) are currently being used in the management of LUTS suggestive of BPH alone or in combination with alpha-1 antagonists [43]. Only one study compared treatment satisfaction between management with tamsulosin 0.4 mg, tadalafil 5 mg and placebo but did not offer separate reporting for older adults [48]. Therefore, no valuable additive information regarding the comparison or combination of treatments in older adults could be delivered by this systematic review.

### Limitations and potential biases

A thorough search process was carried out including the application of the PICOS scheme and a two-step approach in the selection of eligible studies thereafter. Nevertheless, it is possible that relevant publications might have been missed as the detection of studies was limited to the databases used.

The results of the meta-analyses must be interpreted with caution. Only a minor fraction of studies included in this SR is represented in the meta-analyses. The results of only three studies in the case of effect on change of IPSS, QoL-score, and incidence of falls and fractures and two in the case of ADEs and incidence of dementia could be included. This is due to high heterogeneity between the studies regarding interventions, comparators and outcomes. The low number of studies, study participants and imputation of SD values due to non-reporting in the original studies reduce the validity of results considerably.

Several sources not focusing particularly on people ≥ 65 years were included for the formulation of the recommendations. Although the additional sources demonstrated similar effects for all age classes the information base may be regarded as diluted. Publication bias has to be regarded as a potential source of bias and could not be assessed due to methodological inconsistencies and the heterogeneity of outcomes. The quality of evidence was evaluated using established and validated quality appraisal tools. Except for one interventional trial [47] all randomised controlled trials were downgraded mostly due to unclear dealing with missing outcome data (attrition bias) and missing study protocol (reporting bias). The authors with missing study protocols were contacted via e-mail but none responded to the request. The quality of included meta-analyses was labelled low quality and most observational studies were rated good quality.

## Conclusion

### Implications for practice

The use of doxazosin should not be considered as first-line medication for the management of arterial hypertension. The use of alpha-1 antagonists in the management of LUTS suggestive of BPH, however, appears to be promising in reducing urinary symptoms. Thereby, the safety profile of different agents has to be carefully assessed in a patient-oriented manner. Long-term safety and efficacy remain questionable and an assessment of efficacy and safety profile in comparison with other classes of drugs could not be performed.

### Implications for research

Even though many older adults suffer from hypertension and the majority of older men experience LUTS from BPH, only eighteen eligible studies could be identified, primarily due to the age restriction, only two of which are placebo controlled randomized trials. This highlights the lack of evidence for older adults although the largest part of medical interventions is performed in this age class. Additionally, randomised controlled trials with extended follow-up periods are needed to assess the benefits and risks of alpha-1 antagonist treatment in long-term use, providing an enhanced understanding of the real-world use of these medications. To complete the picture of management of LUTS suggestive of BPH in people ≥ 65 years it would be also desirable if future research would focus on comparisons and combinations of different classes of drugs.

Given that most included studies revealed considerable methodological limitations a stronger emphasis should be laid on the application of appropriate methodology. This would produce higher quality results yielding more reliable evidence helping us all to provide the best possible patient care.

## Supplementary Information

**Additional file 1.** Search terms used in the literature database search.

**Additional file 2.** Reasons for exclusion in full text analysis.

**Additional file 3.** Summary of characteristics of included studies.

**Additional file 4.** Summary of patient characteristics of included studies.

**Additional file 5.** Additional information on patient characteristics for each study used in the included meta-analyses. 

## Data Availability

All data generated or analysed during this study are included in this published article [and its supplementary information files].

## Abbreviations

5-ARI: 5-Alpha reductase inhibitor
ACE-inhibitor: Angiotensin converting enzyme inhibitor
ADEs: Adverse drug events
ALLHAT: Antihypertensive and Lipid-Lowering Treatment to Prevent Heart Attack Trial
Alpha-1 antagonists: Adrenergic alpha-1 receptor antagonists
ASCOT: Anglo-Scandinavian Cardiac Outcomes Trial
AUASS: American Urological Association Symptom Score
AUR: Acute urinary retention
BOOI: Bladder Outlet Obstruction Index
BP: Blood pressure
BPH: Benign prostatic hyperplasia
CAD: Coronary artery disease
CASP: Critical Appraisal Skills Programme
CeVD: Cerebrovascular disease
CHD: Coronary heart disease
CHF: Chronic heart failure
CI: Confidence interval
CKD: Chronic kidney disease
COPD: Chronic obstructive pulmonary disease
CV: Cardiovascular
CVD: Cardiovascular disease
DDD: Defined daily dose
EAU: European Association of Urology
ESC: European Society of Cardiology
ESH: European Society of Hypertension
ED: Erectile dysfunction
FORTA: Fit fOR The Aged
GRADE: Grading of Recommendations Assessment, Development and Evaluation
HRQL: Health related quality of life
IIEF-5: International Index of Erectile Function
IPSS: International Prostate Symptom Score
LUTS: Lower urinary tract symptoms
MI: Myocardial infarction
mo: Months
N.a.: Not available
NSAID: Non-steroidal anti-inflammatory drug
OR: Odds ratio
PIM: Potentially inappropriate medication
PPI: Proton pump inhibitor
PVD: Peripheral vascular disease
PVR: Post void residual volume
Qmax: Maximum urinary flow rate
QoL: Quality of Life
RCTs: Randomized controlled trials
RR: Relative Risk
RRdia: Diastolic blood pressure
RRsys: Systolic blood pressure
SD: Standard deviation
SR: Systematic review
SSRI: Selective serotonin reuptake inhibitor
TIA: Transient ischemic attack
TSS-BPH: Treatment Satisfaction Scale for BPH
Vprostate: Prostatic volume
y: Years
