# Supplementary material for: Efficacy and safety of adrenergic alpha-1 receptor antagonists in older adults: a systematic review and meta-analysis supporting the development of recommendations to reduce potentially inappropriate prescribing
Source: BMC Geriatr. 2022 Sep 28;22:771. doi: 10.1186/s12877-022-03415-7 (PMC9516834; doi:10.1186/s12877-022-03415-7)
Supplement: Supplementary file 1 — Additional file 1. Search terms used in the literature database search. [file 12877_2022_3415_MOESM1_ESM.pdf]

Additional file 1 Search terms used in the literature database search

**P(1)**	**Patients**
1	geriatrics.mp. or exp geriatrics/
2	geriatric patient.mp.
3	geriatric*.mp.
4	(elder$ or geriatric$).ab,ti.
5	elder*.mp.
6	frail elderly.mp. or exp frail elderly/
7	aged.mp. or exp Aged/
8	old*.mp.
9	old* adult*.mp.
10	old* people*.mp.
11	>65.mp.
12	over 65.mp.
13	or/1-12
**P(2)**	**Condition**
14	Benign prostatic hyperplasia.mp.
15	Lower urinary tract symptoms.mp.
16	Urination dysorders.mp.

17	Blood Pressure, high.mp.
18	Cardiac Failure.mp.
19	Raynaud's Disease.mp.
20	or/14-19
**I**	**Drugs**
21	Alpha Blockers.mp.
22	Tamsulosin.mp.
23	Doxazosin.mp.
24	Terazosin.mp.
25	Alfuzosin.mp.
26	Or/21-25
**O**	**Outcomes**
27	Mortality.mp.
28	Life expectancy.mp.
29	Quality-adjusted Life Years.mp
30	Quality of Life.mp.
31	QOL.mp.
32	Hospitalization.mp.
33	Adverse effects.mp.

34	Toxicity, Drug.mp.
35	Allergic Reaction.mp.
36	Rash.mp.
37	Safety.mp.
38	Falls.mp.
39	Cognitive Function.mp.
40	Cognitive Decline.mp.
41	Delirium.mp.
42	Lightheadedness.mp OR Light-Headedness.mp
43	Vertigo.mp.
44	Somnolence.mp.
45	Lassitude.mp.
46	Asthenia.mp.
47	Lethargy.mp.
48	Head Pain.mp.
49	Nausea.mp.
50	Emesis.mp.
51	Appetite Loss.mp.

52	Constipation.mp.
53	Diarrhea.mp.
54	Cardiovascular.mp.
55	Cerebrovascular.mp.
56	Low Blood Pressure.mp.
57	Hypotension, postural.mp.
58	Tachycardia.mp.
59	Chest pain.mp.
60	Anasarca.mp. OR Oedema.mp.
61	Dry mouth.mp.
62	Rhinitis.mp.
63	Accommodation.mp.
64	Incontinence.mp.
65	Ejaculation.mp.
66	or/27-65
**S**	**Studies**
67	Cohort studies.mp
68	Case-control studies.mp

69	Randomized controlled trial.mp
70	Non-randomized controlled trial.mp
71	Systematic review.mp
72	Meta-analysis.mp or network meta-analysis.mp
73	or/67-72
**74**	**13 and 20 and 26 and 66 and 73**
