# Supplementary material for: Efficacy and safety of adrenergic alpha-1 receptor antagonists in older adults: a systematic review and meta-analysis supporting the development of recommendations to reduce potentially inappropriate prescribing
Source: BMC Geriatr. 2022 Sep 28;22:771. doi: 10.1186/s12877-022-03415-7 (PMC9516834; doi:10.1186/s12877-022-03415-7)
Supplement: Supplementary file 2 — Additional file 2. Reasons for exclusion in full text analysis. [file 12877_2022_3415_MOESM2_ESM.pdf]

Additional file 2 Reasons for exclusion in full text analysis

**Reference**	**Reason for exclusion**
(2002). "Tamsulosin offers advantages over other α1-adrenoceptor antagonists in the treatment of LUTS." Drugs and Therapy Perspectives 18(10): 1-4.	Wrong study type
(2007). "α-adrenoceptor antagonists best choice for initial treatment of lower urinary tract symptoms related to benign prostatic hyperplasia." Drugs and Therapy Perspectives 23(6): 9-11.	Wrong study type
Abrahams, P., et al. (1995). "Tamsulosin, a selective α(1c)-adrenoceptor antagonist: A randomized, controlled trial in patients with benign prostatic 'obstruction' (symptomatic BPH)." British Journal of Urology 76(3): 325-336.	Wrong age
Abrams, P., et al. (1997). "A dose-ranging study of the efficacy and safety of tamsulosin, the first prostate-selective α(1A)-adrenoceptor antagonist, in patients with benign prostatic obstruction (symptomatic benign prostatic hyperplasia)." British Journal of Urology 80(4): 587-596.	Wrong age
Agrawal, M. S., et al. (2009). "A prospective randomized study comparing alfuzosin and tamsulosin in the management of patients suffering from acute urinary retention caused by benign prostatic hyperplasia." Indian Journal of Urology 25(4): 474-478.	Wrong age
Aikawa, K., et al. (2015). "Elucidation of the Pattern of the Onset of Male Lower Urinary Tract Symptoms Using Cluster Analysis: Efficacy of Tamsulosin in Each Symptom Group." Urology 86(2): 349-353.	Wrong age
Albisinni, S., et al. (2017). "New treatment strategies for benign prostatic hyperplasia in the frail elderly population: A systematic review." Minerva Urologica e Nefrologica 69(2): 119-132.	Wrong study type

Alcaraz, A., et al. (2016). "Quality of life in patients with lower urinary tract symptoms associated with BPH: change over time in real-life practice according to treatment—the QUALIPROST study." International Urology and Nephrology 48(5): 645-656.	Wrong age
Alhawassi, T. M., et al. (2018). "Antihypertensive-related adverse drug reactions among older hospitalized adults." International Journal of Clinical Pharmacy 40(2): 428-435.	Wrong intervention
Ames, R. P., et al. (1989). "Effectiveness of doxazosin in systemic hypertension." American Journal of Cardiology 64(3): 203-208.	Wrong age
Andersen, M., et al. (2000). "Double-blind trial of the efficacy and tolerability of doxazosin in the gastrointestinal therapeutic system, doxazosin standard, and placebo in patients with benign prostatic hyperplasia." European Urology 38(4): 400-409.	Wrong age
Andersson, K. E. (2002). "Alpha-adrenoceptors and benign prostatic hyperplasia: basic principles for treatment with alpha-adrenoceptor antagonists." World Journal of Urology 19(6): 390-396.	Wrong study type
Andersson, P. E., et al. (1994). "Effects of selective alfa1 and beta1-adreno-receptor blockade on lipoprotein and carbohydrate metabolism in hypertensive subjects, with special emphasis on insulin sensitivity." Journal of Human Hypertension 8(3): 219-226.	Wrong age
Anwarul Islam, A. K. M., et al. (2005). "Efficacy of terazosin and finasteride in symptomatic benign prostatic hyperplasia: A comparative study." Bangladesh Medical Research Council Bulletin 31(2): 54-61.	Wrong age

Araki, T., et al. (2013). "Comparison of 7 α1-adrenoceptor antagonists in patients with lower urinary tract symptoms associated with benign prostatic hyperplasia: A short-term crossover study." Acta Medica Okayama 67(4): 245-251.	Wrong age
Arnold, E. P. (2001). "Tamsulosin in men with confirmed bladder outlet obstruction: A clinical and urodynamic analysis from a single centre in New Zealand." BJU International 87(1): 24-31.	Wrong age
Aronow, W. S., et al. (2007). "Cardiovascular drug therapy in the elderly." Cardiology in Review 15(4): 195-215.	Wrong study type
Asplund, R. (2007). "Pharmacotherapy for nocturia in the elderly patient." Drugs and Aging 24(4): 325-343.	Wrong study type
Barbui, P., et al. (2000). "Safety, efficacy and impact on patients' quality of life of a long-term treatment with the α1-blocker alfuzosin in symptomatic patients with BPH." European Urology 37(6): 680-686.	Wrong age
Barkin, J., et al. (2003). "Alpha-blocker therapy can be withdrawn in the majority of men following initial combination therapy with the dual 5α-reductase inhibitor dutasteride." European Urology 44(4): 461-466.	Wrong age
Barrios, V., et al. (2008). "Comparison of the effects of doxazosin and atenolol on target organ damage in adults with type 2 diabetes mellitus and hypertension in the CARDHIAC study: A 9-month, prospective, randomized, open-label, blinded-evaluation trial." Clinical Therapeutics 30(1): 98-107.	Wrong age
Barzilay, J. I., et al. (2004). "Cardiovascular outcomes using doxazosin vs. chlorthalidone for the treatment of hypertension in older adults with and	Wrong age

without glucose disorders: a report from the ALLHAT study." Journal of clinical hypertension (Greenwich, Conn.) 6(3): 116-125.	
Barzilay, J. I., et al. (2001). "Baseline characteristics of the diabetic participants in the antihypertensive and lipid-lowering treatment to prevent heart attack trial (ALLHAT)." Diabetes Care 24(4): 654-658.	Wrong intervention
Batista, J. E., et al. (2002). "Tamsulosin: Effect on quality of life in 2740 patients with lower urinary tract symptoms managed in real life practice in Spain." Archivos Espanoles de Urologia 55(1): 97-105.	Wrong age
Bird, M. R. (2004). "Urinary incontinence in the elderly." Journal of Pharmacy Practice and Research 34(4): 319-321.	Wrong study type
Bird, S. T., et al. (2013). "Tamsulosin treatment for benign prostatic hyperplasia and risk of severe hypotension in men aged 40-85 years in the United States: Risk window analyses using between and within patient methodology." BMJ (Online) 347.	Wrong age
Black, H. R., et al. (2006). "Controlled-release doxazosin as combination therapy in hypertension: the GATES study." Journal of clinical hypertension (Greenwich, Conn.) 8(3): 159-166; quiz 167-168.	Wrong age
Black, H. R., et al. (2000). "The addition of doxazosin to the therapeutic regimen of hypertensive patients inadequately controlled with other antihypertensive medications: A randomized, placebo-controlled study." American Journal of Hypertension 13(5 I): 468-474.	Wrong age
Boston, D. (1995). "Safety, efficacy, and lipid profile of doxazosin at a VA medical center." Hospital Formulary 30(4): 233-236.	Wrong age

Brawer, M. K., et al. (1993). "Terazosin in the treatment of benign prostatic hyperplasia. Terazosin Benign Prostatic Hyperplasia Study Group." Archives of Family Medicine 2(9): 929-935.	Wrong age
Brown, M. J. and J. E. C. Dickerson (1995). "Alpha-blockade and calcium antagonism: An effective and well-tolerated combination for the treatment of resistant hypertension." Journal of Hypertension 13(6): 701-707.	Wrong age
Bruskewitz, R. C. (1992). "Benign prostatic hyperplasia: Drug and nondrug therapies." Geriatrics 47(12): 39-45.	Wrong study type
Butt, T. F., et al. (2010). "Managing hypertension in the very elderly: Effect of adverse drug reactions (ADRs) on achieving targets." Journal of Human Hypertension 24(8): 514-518.	Wrong intervention
Buzelin, J. M., et al. (1993). "Alpha-blocking treatment with alfuzosin in symptomatic benign prostatic hyperplasia: Comparative study with prazosin." British Journal of Urology 72(6): 922-927.	Wrong age
Buzelin, J. M., et al. (1997). "Efficacy and safety of sustained-release alfuzosin 5 mg in patients with benign prostatic hyperplasia." European Urology 31(2): 190-198.	Wrong age
Caine, M. (1990). "Alpha-adrenergic blockers for the treatment of benign prostatic hyperplasia." Urologic Clinics of North America 17(3): 641-649.	Wrong study type
Cambio, A. J. and C. P. Evans (2007). "Outcomes and quality of life issues in the pharmacological management of benign prostatic hyperplasia (BPH)." Therapeutics and Clinical Risk Management 3(1): 181-196.	Wrong age

Canavan, M., et al. (2015). "Does lowering blood pressure with antihypertensive therapy preserve independence in activities of daily living? a systematic review." American Journal of Hypertension 28(2): 273-279.	Wrong age
Cai, T. et al. (2020). „Comparison of Serenoa repens With Tamsulosin in the Treatment of Benign Prostatic Hyperplasia: A Systematic Review and Meta-Analysis." American journal of men's health, 14(2), 1557988320905407.	Wrong age
Cao, Y., et al. (2016). "A randomized, open-label, comparative study of efficacy and safety of tolterodine combined with tamsulosin or doxazosin in patients with benign prostatic hyperplasia." Medical Science Monitor 22: 1895-1902.	Wrong age
Carbin, B. E., et al. (1991). "Efficacy of alfuzosine (an alpha 1-adrenoreceptor blocking drug) in benign hyperplasia of the prostate." Scandinavian journal of urology and nephrology. Supplementum 138: 73-75.	Wrong age
Carbone, A., et al. (2016). "Management of lower urinary tract symptoms associated with benign prostatic hyperplasia in elderly patients with a new diagnostic, therapeutic and care pathway." International Journal of Clinical Practice 70(9): 734-743.	Wrong intervention
Carbone Jr, D. J. and S. Hodges (2003). "Medical therapy for benign prostatic hyperplasia: Sexual dysfunction and impact on quality of life." International Journal of Impotence Research 15(4): 299-306.	Wrong age
Carruthers, G., et al. (1993). "Comparative trial of doxazosin and atenolol on cardiovascular risk reduction in systemic hypertension." American Journal of Cardiology 71(7): 575-581.	Wrong age

Cauchie, P., et al. (1995). "A Belgian post-marketing study of terazosin for the treatment of benign prostate hypertrophy (BPH)." Acta Therapeutica 21(3-4): 257-269.	Wrong age
Cervenakov, I. and J. Fillo (2001). "Our experience with the treatment of benign prostatic hyperplasia (BPH) with tamsulosin." Bratislavské lekárske listy 102(3): 138-141.	Wrong age
Chai, Y. et al. (2021). “Outcomes and complications of naftopidil versus tamsulosin for elderly men with lower urinary tract symptoms secondary to benign prostatic hyperplasia: A systematic review and meta-analysis.” Andrologia, 53(9), e14166.	Wrong age
Chang, S. J., et al. (2008). "The effectiveness of tamsulosin in treating women with voiding difficulty." International Journal of Urology 15(11): 981-985.	Wrong age
Chapple, C. R. (1996). "Selective α1-adrenoceptor antagonists in benign prostatic hyperplasia: Rationale and clinical experience." European Urology 29(2): 129-144.	Wrong study type
Chapple, C. R., et al. (2005). "Tamsulosin oral controlled absorption system (OCAS) in patients with lower urinary tract symptoms suggestive of benign prostatic hyperplasia (LUTS/BPH): Efficacy and tolerability in a placebo and active comparator controlled phase 3a study." European Urology, Supplements 4(2): 33-44.	Wrong age
Chapple, C. R., et al. (1994). "A three month double-blind study of doxazosin as treatment for benign prostatic bladder outlet obstruction." British Journal of Urology 74(1): 50-56.	Wrong age

Chapple, C. R., et al. (2005). "Tamsulosin oral controlled absorption system (OCAS) in patients with lower urinary tract symptoms suggestive of benign prostatic hyperplasia (LUTS/BPH): Efficacy and tolerability in a phase 2b dose-response study." European Urology, Supplements 4(2): 25-32.	Wrong age
Chapple, C. R., et al. (2011). "Silodosin therapy for lower urinary tract symptoms in men with suspected benign prostatic hyperplasia: Results of an international, randomized, double-blind, placebo- and active-controlled clinical trial performed in Europe." European Urology 59(3): 342-352.	Wrong age
Chapple, R., et al. (1996). "Tamsulosin, the first prostate-selective α(1A)-adrenoceptor antagonist. Meta-analysis of two randomized, placebo-controlled, multicentre studies in patients with benign prostatic obstruction (symptomatic BPH)." European Urology 29(2): 155-167.	Wrong age
Cho, H. J. and T. K. Yoo (2014). "Silodosin for the treatment of clinical benign prostatic hyperplasia: Safety, efficacy, and patient acceptability." Research and Reports in Urology 6: 113-119.	Wrong age
Choi, H., et al. (2015). "Assessment of Tamsulosin 0.2 mg for Symptomatic Bladder Outlet Obstruction Secondary to Benign Prostatic Enlargement: Data from a Korean Multicenter Cross-Sectional Study." Urologia Internationalis 95(1): 50-55.	Wrong age
Chow, K. L., et al. (2017). "A review on the management and pharmacological treatments of benign prostatic hyperplasia in primary care." Hong Kong Practitioner 39(4): 116-125.	Wrong study type

Christensen, M. M., et al. (1993). "Doxazosin treatment in patients with prostatic obstruction. A double-blind placebo-controlled study." Scandinavian Journal of Urology and Nephrology 27(1): 39-44.	Wrong age
Chung, B. H. and S. J. Hong (2006). "Long-term follow-up study to evaluate the efficacy and safety of the doxazosin gastrointestinal therapeutic system in patients with benign prostatic hyperplasia with or without concomitant hypertension." BJU International 97(1): 90-95.	Wrong age
Cindolo, L., et al. (2015). "Patient's adherence on pharmacological therapy for benign prostatic hyperplasia (BPH)-associated lower urinary tract symptoms (LUTS) is different: Is combination therapy better than monotherapy." BMC Urology 15(1).	Wrong age
Coetzer, R. (2013). "A review of medicines used to treat benign prostatic hyperplasia." SA Pharmaceutical Journal 80(4): 23-25.	Wrong study type
Colloi, D., et al. (1995). "Multicenter observational trial on symptomatic treatment of benign prostatic hyperplasia with alfuzosin: Clinical evaluation of impact on patient's quality of life." European Urology 27(2): 128-134.	Wrong age
Cox, D. A., et al. (1986). "The antihypertensive effects of doxazosin: A clinical overview." British Journal of Clinical Pharmacology 21(SUPPL. 1): 83S-90S.	Wrong age
Croom, K. F. and A. J. Wagstaff (2004). "Management of benign prostatic hyperplasia: Defining the role of tamsulosin." Disease Management and Health Outcomes 12(5): 337-350.	Wrong study type
Daae, L. N. W. and L. Westlie (1998). "A 5-year comparison of doxazosin and atenolol in patients with mild-to- moderate hypertension: Effects on blood	Wrong age

pressure, serum lipids, and coronary heart disease risk." Blood Pressure 7(1): 39-45.	
Dahm, P., et al. (2017). "Comparative Effectiveness of Newer Medications for Lower Urinary Tract Symptoms Attributed to Benign Prostatic Hyperplasia: A Systematic Review and Meta-analysis." European Urology 71(4): 570-581.	Wrong age
Davis, B. R. (2000). "Major cardiovascular events in hypertensive patients randomized to doxazosin vs chlorthalidone: The antihypertensive and lipid-lowering treatment to prevent heart attack trial (ALLHAT)." Journal of the American Medical Association 283(15): 1967-1975.	Other
Davis, B. R., et al. (2002). "Relationship of antihypertensive treatment regimens and change in blood pressure to risk for heart failure in hypertensive patients randomly assigned to doxazosin or chlorthalidone: Further analyses from the antihypertensive and lipid-lowering treatment to prevent heart attack trial." Annals of Internal Medicine 137(5 I): 313-320.	Wrong age
De Mey, C., et al. (1998). "A double-blind comparison of terazosin and tamsulosin on their differential effects on ambulatory blood pressure and nocturnal orthostatic stress testing." European Urology 33(5): 481-488.	Wrong age
De Reijke, T. M. and P. Klarskov (2004). "Comparative efficacy of two α1;-adrenoreceptor antagonists, doxazosin and alfuzosin, in patients with lower urinary tract symptoms from benign prostatic enlargement." BJU International 93(6): 757-762.	Wrong age
Debruyne, F., et al. (2004). "Evaluation of the clinical benefit of permixon and tamsulosin in severe BPH patients - PERMAL study subset analysis." European Urology 45(6): 773-780.	Wrong age

Debruyne, F. M. J., et al. (1998). "Sustained-release alfuzosin, finasteride and the combination of both in the treatment of benign prostatic hyperplasia." European Urology 34(3): 169-175.	Wrong age
Debruyne, F. M. J., et al. (1996). "The international terazosin trial: A multicentre study of the long-term efficacy and safety of terazosin in the treatment of benign prostatic hyperplasia." European Urology 30(3): 369-376.	Wrong age
Demir, O., et al. (2009). "The effect of α-blocker therapy on erectile functions in patients with lower urinary tract symptoms due to benign prostate hyperplasia." Asian Journal of Andrology 11(6): 716-722.	Wrong age
Dhaliwal, A. S., et al. (2009). "Impact of Alpha 1-Adrenergic Antagonist Use for Benign Prostatic Hypertrophy on Outcomes in Patients With Heart Failure." American Journal of Cardiology 104(2): 270-275.	Wrong age
Dhruva, S. S., et al. (2017). "Heterogeneity in Early Responses in ALLHAT (Antihypertensive and Lipid-Lowering Treatment to Prevent Heart Attack Trial)." Hypertension 70(1): 94-102.	Wrong age
Disantostefano, R. L., et al. (2006). "An evaluation of the economic costs and patient-related consequences of treatments for benign prostatic hyperplasia." BJU International 97(5): 1007-1016.	Wrong intervention
DiSantostefano, R. L., et al. (2006). "The long-term cost effectiveness of treatments for benign prostatic hyperplasia." PharmacoEconomics 24(2): 171-191.	Wrong intervention

Djavan, B. (2004). "α1-Adenoceptor antagonists for the treatment of lower urinary tract symptoms suggestive of benign prostatic hyperplasia (LUTS/BPH): State of the art." European Urology, Supplements 3(4): 23-30.	Wrong study type
Djavan, B., et al. (1999). "A meta-analysis on the efficacy and tolerability of α1-adrenoceptor antagonists in patients with lower urinary tract symptoms suggestive of benign prostatic obstruction." European Urology 36(1): 1-13.	Wrong study type
Djavan, B., et al. (2005). "The impact of tamsulosin oral controlled absorption system (OCAS) on nocturia and the quality of sleep: Preliminary results of a pilot study." European Urology, Supplements 4(2): 61-68.	Wrong age
Dogra, P. N., et al. (2005). "Comparative evaluation of Prostina® and terazosin in the treatment of benign prostatic hyperplasia." Journal of the Indian Medical Association 103(2): 108-112.	Wrong age
Dominguez, L. J., et al. (1995). "Doxazosin lowers blood pressure and improves insulin responses to a glucose load with no changes in tyrosine kinase activity or insulin binding." American Journal of Hypertension 8(5 I): 528-532.	Wrong age
Dunn, C. J., et al. (2002). "Tamsulosin: A review of its pharmacology and therapeutic efficacy in the management of lower urinary tract symptoms." Drugs and Aging 19(2): 135-161.	Wrong study type
Dutkiewics, S. (2001). "Efficacy and tolerability of drugs for treatment of benign prostatic hyperplasia." International Urology and Nephrology 32(3): 423-432.	Other

Dutkiewicz, S. (1995). "A comparative study of: Long-term alpha-1-blocker, Doxazosin therapy versus surgery in the treatment of benign prostatic hyperplasia." Materia Medica Polona 27(4): 151-152.	Wrong age
Dutkiewicz, S. (1996). "Doxazosin - Alpha-1-adrenergic antagonists drug in the long-term (3-years). Management of benign prostatic hyperplasia." Materia Medica Polona 28(3): 93-102.	Wrong age
Dutkiewicz, S. (2004). "Long-term treatment with doxazosin in men with benign prostatic hyperplasia: 10-year follow-up." International Urology and Nephrology 36(2): 169-173.	Wrong age
Dutkiewicz, S. and A. Witeska (1995). "Doxazosin - An alpha-1 receptor blocking agent in the long-term management of benign prostatic hyperplasia (Part one)." International Urology and Nephrology 27(3): 311-318.	Wrong age
Egilmez, T., et al. (2006). "Effects of selective alpha-1-adrenergic receptor blockers on bladder weight." Urologia Internationalis 76(1): 42-50.	Wrong age
Elhilali, M., et al. (2006). "Long-term efficacy and safety of alfuzosin 10 mg once daily: A 2-year experience in 'real-life' practice." BJU International 97(3): 513-519.	Wrong age
Englert, R. G. and H. Mauersberger (1988). "A single-blind study of doxazosin in the treatment of essential hypertension when added to nonresponders to angiotensin-converting enzyme inhibitor therapy." American Heart Journal 116(6 II SUPPL.): 1826-1832.	Wrong age

Fabricius, P. G. and W. Vahlensieck Jr (1996). "Alpha1 receptor blockers in benign prostatic hypertrophy. Effectiveness in nonsurgical patients with BPH symptoms." Munchener Medizinische Wochenschrift 138(1-2): 8-12.	Wrong age
Fabricius, P. G., et al. (1990). "Efficacy of once-a-day terazosin in benign prostatic hyperplasia: a randomized, double-blind placebo-controlled clinical trial." The Prostate. Supplement 3: 85-93.	Wrong age
Fawzy, A., et al. (1995). "Doxazosin in the treatment of benign prostatic hyperplasia in normotensive patients: A multicenter study." Journal of Urology 154(1): 105-109.	Wrong age
Fawzy, A., et al. (1999). "Long-term (4 year) efficacy and tolerability of doxazosin for the treatment of concurrent benign prostatic hyperplasia and hypertension." International Journal of Urology 6(7): 346-354.	Wrong age
Fine, S. R. and P. Ginsberg (2008). "Alpha-adrenergic receptor antagonists in older patients with benign prostatic hyperplasia: Issues and potential complications." Journal of the American Osteopathic Association 108(7): 333-337.	Wrong study type
Fitzpatrick, J. M. and F. Desgrandchamps (2005). "The clinical efficacy and tolerability of doxazosin standard and gastrointestinal therapeutic system for benign prostatic hyperplasia." BJU International 95(4): 575-579.	Wrong study type
Flack, J. M. (2002). "The effect of doxazosin on sexual function in patients with benign prostatic hyperplasia, hypertension, or both." International Journal of Clinical Practice 56(7): 527-530.	Wrong study type

Flannery, M. T., et al. (2006). "Efficacy and safety of tamsulosin for benign prostatic hyperplasia: Clinical experience in the primary care setting." Current Medical Research and Opinion 22(4): 721-730.	Wrong age
Fonseca, J. and C. Martins da Silva (2015). "The Diagnosis and Treatment of Lower Urinary Tract Symptoms due to Benign Prostatic Hyperplasia with α-Blockers: Focus on Silodosin." Clinical Drug Investigation 35(1): 7-18.	Wrong study type
Fourcade, R. O., et al. (2012). "Outcomes and general health-related quality of life among patients medically treated in general daily practice for lower urinary tract symptoms due to benign prostatic hyperplasia." World Journal of Urology 30(3): 419-426.	Wrong age
Frankel, J. K., et al. (2018). "Is Tamsulosin Linked to Dementia in the Elderly?" Current Urology Reports 19(9).	Wrong study type
Fulton, B., et al. (1995). "Doxazosin: An update of its clinical pharmacology and therapeutic applications in hypertension and benign prostatic hyperplasia." Drugs 49(2): 295-320.	Wrong study type
Gillenwater, J. Y., et al. (1995). "Doxazosin for the treatment of benign prostatic hyperplasia in patients with mild to moderate essential hypertension: A double-blind, placebo- controlled, dose-response multicenter study." Journal of Urology 154(1): 110-115.	Wrong age
Giorgi, G., et al. (1988). "A comparative study of doxazosin versus atenolol in mild-to-moderate hypertension." American Heart Journal 116(6 II SUPPL.): 1801-1805.	Wrong age

Gokkaya, C. S., et al. (2015). "Flurbiprofen alone and in combination with alfuzosin for the management of lower urinary tract symptoms." Central European Journal of Urology 68(1): 51-56.	Wrong age
Gopi, S. S., et al. (2006). "A prospective pilot study to validate the management protocol for patients presenting with acute urinary retention: a community-based, nonhospitalised protocol." TheScientificWorldJournal 6: 2436-2441.	Wrong age
Gotoh, M., et al. (2012). "Correlations among Lower Urinary Tract Symptoms, Bother, and Quality of Life in Patients with Benign Prostatic Hyperplasia and Associated Fluctuations with Tamsulosin Administration." LUTS: Lower Urinary Tract Symptoms 4(1): 45-50.	Wrong age
Grasso, M., et al. (1995). "Comparative effects of alfuzosin versus Serenoa repens in the treatment of symptomatic benign prostatic hyperplasia." Archivos Espanoles de Urologia 48(1): 97-103.	Wrong age
Graybill, S. D. and R. A. Vigersky (2015). "Effects of α-blocker therapy on active duty military and military retirees for benign prostatic hypertrophy on diabetic complications." Military medicine 180(3): 355-360.	Wrong age
Grimm Jr, R. H., et al. (1996). "α-Blockade and thiazide treatment of hypertension. A double-blind randomized trial comparing doxazosin and hydrochlorothiazide." American Journal of Hypertension 9(5): 445-454.	Wrong age
Gross, A. J., et al. (2005). "Switch from phytotherapy to tamsulosin in patients with lower urinary tract symptoms suggestive of benign prostatic hyperplasia (LUTS/BPH)." Prostate Cancer and Prostatic Diseases 8(3): 210-214.	Wrong age
Guo, B. et al. (2020). „Comparative Effectiveness of Tadalafil versus Tamsulosin in Treating Lower Urinary Tract Symptoms Suggestive of Benign	Wrong age

Prostate Hyperplasia: A Meta-Analysis of Randomized Controlled Trials." Medical science monitor : international medical journal of experimental and clinical research, 26, e923179.	
Guthrie, R. (1997). "Doxazosin for benign prostatic hyperplasia in primary care." Clinical Therapeutics 19(6): 1269-1277.	Wrong study type
Guthrie, R. M. and R. L. Siegel (1999). "A multicenter, community-based study of doxazosin in the treatment of concomitant hypertension and symptomatic benign prostatic hyperplasia: The hypertension and BPH intervention trial (HABIT)." Clinical Therapeutics 21(10): 1732-1748.	Wrong age
Hadi, N., et al. (2013). "Superselective α-adrenergic blockers versus transurethral resection of the prostate: a prospective comparison of health-related quality of life outcome after treating patients with benign prostatic hyperplasia." Quality of life research : an international journal of quality of life aspects of treatment, care and rehabilitation 22(6): 1287-1293.	Wrong age
Haillot, O., et al. (2011). "The effects of combination therapy with dutasteride plus tamsulosin on clinical outcomes in men with symptomatic BPH: 4-year post hoc analysis of European men in the CombAT study." Prostate Cancer and Prostatic Diseases 14(4): 302-306.	Wrong age
Hansen, B. J., et al. (1996). "Symptomatic outcome of transurethral prostatectomy, alpha-blockade and placebo in the treatment of benign prostatic hyperplasia. Evaluation of treatment with the danish prostatic symptom score (DAN-PSS-1) system." Scandinavian Journal of Urology and Nephrology 30(2): 103-107.	Wrong age

Hansen, B. J., et al. (1994). "Alfuzosin in the treatment of benign prostatic hyperplasia: Effects on symptom scores, urinary flow rates and residual volume. A multicentre, double-blind, placebo-controlled trial." Scandinavian Journal of Urology and Nephrology, Supplement(157): 169-175.	Wrong age
Hansson, L. (1988). "Implications of doxazosin therapy on risk of coronary heart disease." American Heart Journal 116(6 II SUPPL.): 1832-1837.	Wrong study type
Hartung, R., et al. (2006). "Age, comorbidity and hypertensive co-medication do not affect cardiovascular tolerability of 10 mg alfuzosin once daily." Journal of Urology 175(2): 624-628.	Wrong age
Hayduk, K. (1987). "Efficacy and safety of doxazosin in hypertension therapy." American Journal of Cardiology 59(14): 35G-39G.	Wrong age
Höfner, K. (1998). "Efficacy and safety of slow-release afluzosin in benign prostate hyperplasia. Experience of a controlled study with 11,562 patients." Urologe - Ausgabe B 38(5): 452-457.	Wrong age
Hofner, K., et al. (1999). "Tamsulosin 0.4 mg once daily: Effect on sexual function in patients with lower urinary tract symptoms suggestive of benign prostatic obstruction." European Urology 36(4): 335-341.	Wrong study type
Höfner, K. and U. Jonas (2002). "Alfuzosin: a clinically uroselective alpha1-blocker." World Journal of Urology 19(6): 405-412.	Wrong study type
Horváth, K., et al. (2006). "A novel approach to the treatment of benign prostatic hyperplasia." BJU International 97(6): 1252-1255.	Wrong age

Hosmane, B. S., et al. (1992). "Effect of age and dose on the incidence of adverse events in the treatment of hypertension in patients receiving terazosin." Journal of Clinical Pharmacology 32(5): 434-443.	Wrong study type
Ichihara, K., et al. (2018). "Silodosin as second-line α-blocker monotherapy in patients with benign prostatic hyperplasia: A prospective observational study." International Journal of Urology 25(10): 849-854.	Wrong age
Ichioka, K., et al. (2004). "Long-term treatment outcome of tamsulosin for benign prostatic hyperplasia." International Journal of Urology 11(10): 870-875.	Wrong age
Ito, H., et al. (2012). "Male Lower Urinary Tract Symptoms: Hypertension as a Risk Factor for Storage Symptoms, but Not Voiding Symptoms." LUTS: Lower Urinary Tract Symptoms 4(2): 68-72.	Wrong age
Itskovitz, H. D. (1991). "Alpha1 blockers: Safe, effective treatment for hypertension." Postgraduate Medicine 89(8): 89-92+95-98+103-104+106+111-112.	Wrong study type
Itskovitz, H. D. (1994). "Alpha1-blockade for the treatment of hypertension: A megastudy of terazosin in 2214 clinical practice settings." Clinical Therapeutics 16(3): 490-504.	Wrong intervention
Janknegt, R. A. and C. R. Chapple (1993). "Efficacy and safety of the alpha-1 blocker doxazosin in the treatment of benign prostatic hyperplasia." European Urology 24(3): 319-326.	Wrong age
Jardin, A., et al. (1991). "Alfuzosin for treatment of benign prostatic hypertrophy." Lancet 337(8755): 1457-1461.	Wrong age

Jardin, A., et al. (1993). "Long-term treatment of benign prostatic hyperplasia with alfuzosin: A 12-18 month assessment." British Journal of Urology 72(5 I): 615-620.	Wrong age
Jin, Z., et al. (2011). "An open, comparative, multicentre clinical study of combined oral therapy with sildenafil and doxazosin GITS for treating Chinese patients with erectile dysfunction and lower urinary tract symptoms secondary to benign prostatic hyperplasia." Asian Journal of Andrology 13(4): 630-635.	Wrong age
Johnson, I. T. M., et al. (2003). "Changes in nocturia from medical treatment of benign prostatic hyperplasia: Secondary analysis of the Department of Veterans Affairs Cooperative Study Trial." Journal of Urology 170(1): 145-148.	Wrong age
Jones, D. W. and C. D. Sands (1993). "Effects of doxazosin and hydrochlorothiazide on lipid levels in Korean patients with essential hypertension." Journal of Cardiovascular Pharmacology 22(3): 431-437.	Wrong age
Kageyama, S., et al. (2003). "Clinical effects of the α1 receptor-blocker, urapidil, on the irritative symptoms of benign prostate hyperplasia." Nishinihon Journal of Urology 65(8): 519-528.	Wrong age
Kaplan, S. A. (2004). "Use of alpha-adrenergic inhibitors in treatment of benign prostatic hyperplasia and implications on sexual function." Urology 63(3): 428-434.	Wrong study type
Kaplan, S. A. and P. M. D'Alisera (1998). "Tolerability of α-blockade with doxazosin as a therapeutic option for symptomatic benign prostatic hyperplasia in the elderly patient: A pooled analysis of seven double-blind, placebo-	Other

controlled studies." Journals of Gerontology - Series A Biological Sciences and Medical Sciences 53(3): M201-M206.	
Kaplan, S. A., et al. (2006). "Combination therapy with doxazosin and finasteride for benign prostatic hyperplasia in patients with lower urinary tract symptoms and a baseline total prostate volume of 25 Ml or greater." Journal of Urology 175(1): 217-220.	Wrong age
Kaplan, S. A., et al. (1995). "Doxazosin in physiologically and pharmacologically normotensive men with benign prostatic hyperplasia." Urology 46(4): 512-517.	Wrong age
Kaplan, S. A., et al. (1997). "The treatment of benign prostatic hyperplasia with alpha blockers in men over the age of 80 years." British Journal of Urology 80(6): 875-879.	Other
Kawabe, K., et al. (2006). "Silodosin, a new α1A-adrenoceptor-selective antagonist for treating benign prostatic hyperplasia: Results of a phase III randomized, placebo-controlled, double-blind study in Japanese men." BJU International 98(5): 1019-1024.	Wrong age
Kawachi, Y. (1998). "Effect of tamsulosin on urodynamics in benign prostatic hypertrophy." Current Therapeutic Research - Clinical and Experimental 59(3): 149-161.	Wrong age
Kim, H. L., et al. (2001). "Results of treatment with tamsulosin in men with acute urinary retention." Techniques in Urology 7(4): 256-260.	Wrong age

Kim, J. H., et al. (2012). "Treatment satisfaction with low-dose tamsulosin for symptomatic benign prostatic hyperplasia: Results from a multicentre cross-sectional survey." International Journal of Clinical Practice 66(12): 1209-1215.	Wrong age
Kim, K. S., et al. (2016). "Tamsulosin Treatment Affecting Patient-reported Outcomes in Benign Prostatic Hyperplasia-associated Depressive Symptoms." Urology 87: 172-177.	Wrong age
Kim, S. C., et al. (2011). "Tadalafil Administered Once Daily for Treatment of Lower Urinary Tract Symptoms in Korean men with Benign Prostatic Hyperplasia: Results from a Placebo-Controlled Pilot Study Using Tamsulosin as an Active Control." LUTS: Lower Urinary Tract Symptoms 3(2): 86-93.	Wrong age
Kim, S. O., et al. (2014). "The α1 adrenoceptor antagonist tamsulosin for the treatment of voiding symptoms improves nocturia and sleep quality in women." Urology journal 11(3): 1636-1641.	Wrong age
Kirby, R. S. (2003). "A randomized, double-blind crossover study of tamsulosin and controlled-release doxazosin in patients with benign prostatic hyperplasia." BJU International 91(1): 41-44.	Wrong age
Kirby, R. S., et al. (2001). "A combined analysis of double-blind trials of the efficacy and tolerability of doxazosin-gastrointestinal therapeutic system, doxazosin standard and placebo in patients with benign prostatic hyperplasia." BJU International 87(3): 192-200.	Wrong age
Kirby, R. S., et al. (2005). "Efficacy of extended-release doxazosin and doxazosin standard in patients with concomitant benign prostatic hyperplasia and sexual dysfunction." BJU International 95(1): 103-109.	Wrong age

Kongkanand, A., et al. (2009). "Safety and efficacy of a prolonged-release formulation of alfuzosin 10 mg once daily in patients with lower urinary tract symptoms suggestive of benign prostatic hyperplasia." Journal of the Medical Association of Thailand 92(7): 969-978.	Wrong age
Kosugi, S., et al. (2007). "A comparative study assessing clinical effects of naftopidil and tamsulosin hydrochloride on benign prostatic hyperplasia with overactive bladder." Japanese Journal of Urology 98(5): 691-699.	Other
Koval, P. G. and T. McDiarmid (2000). "How effective is doxazosin compared with chlorthalidone in the treatment of hypertension?" The Journal of family practice 49(7): 597-598.	Wrong study type
Kumar, R., et al. (2009). "α-Blocker Use Is Associated With Decreased Risk of Sexual Dysfunction." Urology 74(1): 82-87.	Wrong age
Kuritzky, L., et al. (2006). "Efficacy and safety of alfuzosin 10 mg once daily in the treatment of symptomatic benign prostatic hyperplasia." International Journal of Clinical Practice 60(3): 351-358.	Wrong study type
Kwak, C., et al. (2007). "High-dose terazosin therapy (5mg) in Korean patients with lower urinary tract symptoms with or without concomitant hypertension: A prospective, open-label study." Yonsei Medical Journal 48(6): 994-1000.	Wrong age
Langdon, C. G. and R. S. Packard (1994). "Doxazosin in hypertension: Results of a general practice study in 4809 patients." British Journal of Clinical Practice 48(6): 293-298.	Wrong intervention
Lapitan, M. C. M., et al. (2005). "A comparative study on the safety and efficacy of tamsulosin and alfuzosin in the management of symptomatic benign	Wrong age

prostatic hyperplasia: A randomized controlled clinical trial." Journal of International Medical Research 33(5): 562-573.	
Lee, C. L. and H. C. Kuo (2018). "Tailoring Medication for Lower Urinary Tract Symptoms in Men Based on International Prostate Symptom Score Voiding to Storage Ratio." Urology 120: 30-35.	Wrong age
Lee, C. L. and H. C. Kuo (2019). "Male patients with a higher frequency of nocturnal urinary episodes are more likely to benefit from alpha-blocker therapy for bothersome nocturia." LUTS: Lower Urinary Tract Symptoms 11(2): O174-O179.	Wrong age
Lee, E. (2002). "Comparison of tamsulosin and finasteride for lower urinary tract symptoms associated with benign prostatic hyperplasia in Korean patients." Journal of International Medical Research 30(6): 584-590.	Wrong age
Lee, H. S., et al. (2012). "Efficacy and safety of tamsulosin for treating lower urinary tract symptoms associated with benign prostatic hyperplasia: A multicenter, randomized, controlled, open-label non-inferiority study." Korean Journal of Urology 53(3): 178-183.	Wrong age
Lee, J. Y., et al. (2012). "Effect of Discontinuation of Tamsulosin in Korean Men with Benign Prostatic Hyperplasia Taking Tamsulosin and Dutasteride: An Open-Label, Prospective, Randomized Pilot Study." LUTS: Lower Urinary Tract Symptoms 4(1): 35-40.	Wrong age
Lee, K. S., et al. (2010). "Efficacy and safety of tamsulosin for the treatment of non-neurogenic voiding dysfunction in females: a 8-week prospective study." Journal of Korean medical science 25(1): 117-122.	Wrong age

Lee, S. H., et al. (2010). "Effects of α-blocker 'add on' treatment on blood pressure in symptomatic BPH with or without concomitant hypertension." Prostate Cancer and Prostatic Diseases 13(4): 333-337.	Wrong age
Lee, S. N., et al. (2017). "Age Related Differences in Responsiveness to Sildenafil and Tamsulosin are due to Myogenic Smooth Muscle Tone in the Human Prostate." Scientific reports 7(1): 10150.	Wrong age
Leliefeld, H. H. J., et al. (2002). "Sexual function before and after various treatments for symptomatic benign prostatic hyperplasia." BJU International 89(3): 208-213.	Wrong age
Lemmer, B. and G. Nold (2003). "Effect of doxazosin GITS on 24-hour blood pressure profile in patients with stage 1 to stage 2 primary hypertension." Blood Pressure Monitoring 8(3): 119-125.	Wrong age
Lepor, H., et al. (1992). "A randomized, placebo-controlled multicenter study of the efficacy and safety of terazosin in the treatment of benign prostatic hyperplasia." Journal of Urology 148(5 I): 1467-1474.	Wrong age
Lepor, H., et al. (1991). "The efficacy and safety of terazosin for the treatment of symptomatic BPH." Prostate 18(4): 345-355.	Wrong age
Lepor, H., et al. (2000). "The mechanism of adverse events associated with terazosin: An analysis of the Veterans Affairs cooperative study." Journal of Urology 163(4): 1134-1137.	Wrong study type
Lepor, H., et al. (1997). "Doxazosin for benign prostatic hyperplasia: Long-term efficacy and safety in hypertensive and normotensive patients." Journal of Urology 157(2): 525-530.	Wrong age

Lepor, H., et al. (1990). "A dose titration study evaluating terazosin, a selective, once-a-day α1-blocker for the treatment of symptomatic benign prostatic hyperplasia." Journal of Urology 144(6): 1393-1397.	Wrong age
Lepor, H., et al. (1992). "The safety, efficacy and compliance of terazosin therapy for benign prostatic hyperplasia." Journal of Urology 147(6): 1554-1557.	Wrong age
Lepor, H. and C. Theune (1995). "Randomized double-blind study comparing the efficacy of terazosin versus placebo in women with prostatism-like symptoms." Journal of Urology 154(1): 116-118.	Wrong age
Lepor, H., et al. (1996). "The efficacy of terazosin, finasteride, or both in benign prostatic hyperplasia." New England Journal of Medicine 335(8): 533-539.	Wrong age
Leungwattanakij, S., et al. (2010). "Sexuality and Management of Benign Prostatic Hyperplasia with Alfuzosin: SAMBA Thailand." Journal of Sexual Medicine 7(9): 3115-3126.	Wrong age
Levy, D., et al. (1996). "Principal results of the Hypertension and Lipid Trial (HALT): A multicenter study of doxazosin in patients with hypertension." American Heart Journal 131(5): 966-973.	Wrong age
Li, N. C., et al. (2003). "Efficacy of Low-Dose Tamsulosin in Chinese Patients with Symptomatic Benign Prostatic Hyperplasia." Clinical Drug Investigation 23(12): 781-787.	Wrong age
Liao, C. H., et al. (2012). "Therapeutic effect of α-blockers and antimuscarinics in male lower urinary tract symptoms based on the International Prostate	Wrong age

Symptom Score subscore ratio." International Journal of Clinical Practice 66(2): 139-145.	
Liguori, G., et al. (2009). "Efficacy and safety of combined oral therapy with tadalafil and alfuzosin: An integrated approach to the management of patients with lower urinary tract symptoms and erectile dysfunction. Preliminary report." Journal of Sexual Medicine 6(2): 544-552.	Wrong age
Lin, H. Z. et al. (2020). "Systemic Alpha1-Adrenoceptor Antagonists and Increased Risk of Open-Angle Glaucoma: A Nationwide Population-Based Cohort Study." Investigative ophthalmology & visual science, 61(10), 15.	Wrong age
Lindner, U. K., et al. (1988). "The addition of doxazosin to the treatment regimen of hypertensive patients not responsive to nifedipine." American Heart Journal 116(6 II SUPPL.): 1814-1820.	Wrong age
Linnebur, S. A. (2004). "Pharmacotherapy update: What's new about old medications." Annals of Long-Term Care 12(9): 34-44.	Wrong study type
Liu, H., et al. (2009). "Efficacy of Combined Amlodipine/Terazosin Therapy in Male Hypertensive Patients With Lower Urinary Tract Symptoms: A Randomized, Double-blind Clinical Trial." Urology 74(1): 130-136.	Wrong age
Lucas, M. G., et al. (2005). "Tamsulosin in the management of patients in acute urinary retention from benign prostatic hyperplasia." BJU International 95(3): 354-357.	Wrong age
Lund-Johansen, P. and P. Omvik (1991). "Acute and chronic hemodynamic effects of drugs with different actions on adrenergic receptors: A comparison	Wrong age

between alpha blockers and different types of beta blockers with and without vasodilating effect." Cardiovascular Drugs and Therapy 5(3): 605-616.	
MacDiarmid, S. A., et al. (1999). "A randomized double-blind study assessing 4 versus 8 mg. doxazosin for benign prostatic hyperplasia." Journal of Urology 162(5): 1629-1632.	Wrong age
Machado-Leiva, M., et al. (2016). "Clinical evaluation of patients with benign prostatic hyperplasia, treated with the natural product Calprost®: A randomized, controlled study." Journal of Pharmacy and Pharmacognosy Research 4(5): 187-198.	Wrong age
Malde, S., et al. (2019). "Incidence of Nocturia in Men with Lower Urinary Tract Symptoms Associated with Benign Prostatic Enlargement and Outcomes After Medical Treatment: Results from the Evolution European Association of Urology Research Foundation Prospective Multinational Registry." European Urology Focus.	Wrong age
Man In, T. V. A. J., et al. (1998). "Drug treatment of hypertension in the elderly: The role of α-adrenoceptor blockade." British Journal of Urology, Supplement 81(1): 21-25.	Wrong study type
Marshall, H. J. and D. G. Beevers (1996). "α-Adrenoceptor blocking drugs and female urinary incontinence: Prevalence and reversibility." British Journal of Clinical Pharmacology 42(4): 507-509.	Wrong age
Martell, N. and M. Luque (2001). "Doxazosin added to single-drug therapy in hypertensive patients with benign prostatic hypertrophy." Journal of Clinical Hypertension 3(4): 218-223.	Wrong age

Martorana, G., et al. (1997). "Effects of short-term treatment with the α1-blocker alfuzosin on urodynamic pressure/flow parameters in patients with benign prostatic hyperplasia." European Urology 32(1): 47-53.	Wrong age
Masuo, K., et al. (1996). "Changes in frequency of orthostatic hypotension in elderly hypertensive patients under medications." American Journal of Hypertension 9(3): 263-268.	Other
McConnell, J. D., et al. (2003). "The Long-Term Effect of Doxazosin, Finasteride, and Combination Therapy on the Clinical Progression of Benign Prostatic Hyperplasia." New England Journal of Medicine 349(25): 2387-2398.	Wrong age
McNeil, J. J., et al. (1987). "Effect of age on pharmacokinetics of and blood pressure responses to prazosin and terazosin." Journal of Cardiovascular Pharmacology 10(2): 168-175.	Wrong age
Michel, M. C., et al. (2001). "Does the time of administration (morning or evening) affect the tolerability or efficacy of tamsulosin?" BJU International 87(1): 31-34.	Wrong age
Miekos, E., et al. (2002). "The efficacy of terazosin for treating benign prostatic hyperplasia: A multicentre clinical trial." BJU International 89(7): 771-772.	Wrong age
Miyakita, H., et al. (2010). "Short-term effects of crossover treatment with silodosin and tamsulosin hydrochloride for lower urinary tract symptoms associated with benign prostatic hyperplasia." International Journal of Urology 17(10): 869-875.	Wrong age

Miyazawa, Y., et al. (2001). "Pharmacokinetics and safety of tamsulosin in subjects with normal and impaired renal or hepatic function." Current Therapeutic Research - Clinical and Experimental 62(9): 603-621.	Wrong age
Mohanty, N. K., et al. (2003). "Doxazocin in management of benign prostatic hyperplasia." Indian Journal of Urology 19(2): 113-116.	Wrong age
Momose, H., et al. (2007). "Crossover comparison study on the therapeutic effects of tamsulosin hydrochloride and naftopidil in lower urinary tract symptoms associated with benign prostatic hyperplasia." Drugs of Today 43(SUPPL. A): 1-10.	Wrong age
Nadeem, H. M. R., et al. (2017). "Comparison of Anti BPH capsule (herbal) and Terazosin HCl in the treatment of benign prostate hyperplasia." Pakistan journal of pharmaceutical sciences 30(1): 289-293.	Wrong age
Nordling, J. (2005). "Efficacy and safety of two doses (10 and 15 mg) of alfuzosin or tamsulosin (0.4 mg) once daily for treating symptomatic benign prostatic hyperplasia." BJU International 95(7): 1006-1012.	Wrong age
Oelke, M., et al. (2012). "Monotherapy with tadalafil or tamsulosin similarly improved lower urinary tract symptoms suggestive of benign prostatic hyperplasia in an international, randomised, parallel, placebo-controlled clinical trial." European Urology 61(5): 917-925.	Wrong age
Olowofela, A. O. and A. O. Isah (2017). "A profile of adverse effects of antihypertensive medicines in a tertiary care clinic in Nigeria." Annals of African Medicine 16(3): 114-119.	Wrong age

Olsen, H., et al. (1999). "Adverse drug reactions in current antihypertensive therapy: A general practice survey of 2586 patients in Norway." Blood Pressure 8(2): 94-101.	Wrong age
Os, I. and H. P. Stokke (1999). "Doxazosin GITS compared with doxazosin standard and placebo in patients with mild hypertension." Blood Pressure 8(3): 184-191.	Wrong age
Os, I. and H. P. Stokke (1999). "Effects of doxazosin in the gastrointestinal therapeutic system formulation versus doxazosin standard and placebo in mild-to-moderate hypertension." Journal of Cardiovascular Pharmacology 33(5): 791-797.	Wrong age
Palit, V., et al. (2005). "Long term follow up of men with Alfuzosin who voided successfully following acute urinary retention." International Urology and Nephrology 37(3): 507-510.	Wrong intervention
Peters, R., et al. (2015). "Antihypertensive drug use and risk of cognitive decline in the very old: An observational study - The Newcastle 85+ Study." Journal of Hypertension 33(10): 2156-2164.	Wrong intervention
Pompeo, A. C. L., et al. (2006). "A randomised, double-blind study comparing the efficacy and tolerability of controlled-release doxazosin and tamsulosin in the treatment of benign prostatic hyperplasia in Brazil." International Journal of Clinical Practice 60(10): 1172-1177.	Wrong age
Pöyhönen, A., et al. (2014). "Outcomes of medical and surgical treatment for lower urinary tract symptoms (benign prostatic obstruction) - A population-based cohort study." International Journal of Clinical Practice 68(3): 349-355.	Wrong age

Prieto, L., et al. (2008). "Efficacy of doxazosin in the treatment of acute urinary retention due to benign prostate hyperplasia." Urologia Internationalis 81(1): 66-71.	Wrong age
Pummangura, N. and W. Kochakarn (2007). "Efficacy of tamsulosin in the treatment of lower urinary tract symptoms (LUTS) in women." Asian Journal of Surgery 30(2): 131-137.	Wrong age
Quek, K. F., et al. (2000). "The psychological effects of treatments for lower urinary tract symptoms." BJU International 86(6): 630-633.	Wrong age
Quek, K. F., et al. (2004). "Effect of treating lower urinary tract symptoms on anxiety, depression and psychiatric morbidity: A one-year study." International Journal of Urology 11(10): 848-855.	Wrong age
Ram, C. V., et al. (1987). "Antihypertensive therapy in the elderly. Effects on blood pressure and cerebral blood flow." The American journal of medicine 82(1 A): 53-57.	Wrong age
Rashidi, A. and J. T. Wright Jr (2009). "Drug Treatment of Hypertension in Older Hypertensives." Clinics in Geriatric Medicine 25(2): 235-244.	Wrong study type
Rhalimi, M., et al. (2009). "Medication use and increased risk of falls in hospitalized elderly patients: A retrospective, case-control study." Drugs and Aging 26(10): 847-852.	Wrong intervention
Roehrborn, C. G., et al. (2005). "A double-blind placebo-controlled study evaluating the onset of action of doxazosin gastrointestinal therapeutic system in the treatment of benign prostatic hyperplasia." European Urology 48(3): 445-452.	Wrong age

Roehrborn, C. G., et al. (2008). "The effects of dutasteride, tamsulosin and combination therapy on lower urinary tract symptoms in men with benign prostatic hyperplasia and prostatic enlargement: 2-year results from the CombAT study." Journal of Urology 179(2): 616-621.	Wrong age
Roehrborn, C. G., et al. (2010). "The Effects of Combination Therapy with Dutasteride and Tamsulosin on Clinical Outcomes in Men with Symptomatic Benign Prostatic Hyperplasia: 4-Year Results from the CombAT Study." European Urology 57(1): 123-131.	Wrong age
Roehrborn, C. G., et al. (2012). "Quantifying the Contribution of symptom improvement to satisfaction of men with moderate to severe benign prostatic hyperplasia: 4-year data from the CombAT trial." Journal of Urology 187(5): 1732-1738.	Wrong age
Rosen, R. C., et al. (2009). "Association of Sexual Dysfunction With Lower Urinary Tract Symptoms of BPH and BPH Medical Therapies: Results From the BPH Registry." Urology 73(3): 562-566.	Wrong age
Schall, P. and M. Wehling (2011). "Treatment of arterial hypertension in the very elderly: A meta-analysis of clinical trials." Arzneimittel-Forschung/Drug Research 61(4): 221-228.	Wrong intervention
Schulman, C. C., et al. (1994). "Belgian multicenter clinical study of alfuzosin, a selective alpha1blocker, in the treatment of benign prostatic hyperplasia." Acta Urologica Belgica 62(4): 15-21.	Wrong age
Seedat, Y. K. and I. P. Naiker (1997). "A single-masked study comparing doxazosin and enalapril in patients with non-insulin-dependent diabetes	Wrong age

mellitus and hypertension." Current Therapeutic Research - Clinical and Experimental 58(9): 633-652.	
Seftel, A. (2005). "Alfuzosin 10 mg once daily improves sexual function in men with lower urinary tract symptoms and concomitant sexual dysfunction." The Journal of urology 174(5): 1940-1941.	Wrong study type
Sengupta, G., et al. (2011). "Comparison of Murraya koenigii- and Tribulus terrestris-Based Oral Formulation Versus Tamsulosin in the Treatment of Benign Prostatic Hyperplasia in Men Aged >50 Years: A Double-Blind, Double-Dummy, Randomized Controlled Trial." Clinical Therapeutics 33(12): 1943-1952.	Wrong age
Shakir, S., et al. (2001). "Finasteride and tamsulosin used in benign prostatic hypertrophy: A review of the prescription-event monitoring data." BJU International 87(9): 789-796.	Wrong age
Shirakawa, T., et al. (2013). "Silodosin versus naftopidil in japanese patients with lower urinary tract symptoms associated with benign prostatic hyperplasia: A randomized multicenter study." International Journal of Urology 20(9): 903-910.	Wrong age
Simaioforidis, V., et al. (2011). "Tamsulosin versus transurethral resection of the prostate: Effect on nocturia as a result of benign prostatic hyperplasia." International Journal of Urology 18(3): 243-248.	Wrong age
Smyth, P., et al. (1988). "24-hour control of blood pressure by once daily doxazosin: A multicentre double-blind comparison with placebo." European Journal of Clinical Pharmacology 34(6): 613-618.	Wrong age

Suman, D., et al. (2015). "Prospective observational study to assess the safety and efficacy of once-daily Tamcontin® tablet (Continus® controlled release tablet of tamsulosin hydrochloride, 0.4 mg) in the treatment of lower urinary tract symptoms secondary to benign prostatic hyperplasia (BPH) in the routine clinical practice- an indian experience." Research Journal of Pharmaceutical, Biological and Chemical Sciences 6(1): 1396-1401.	Wrong age
Sung, H. H. et al. (2020). „Efficacy and Safety of Naftopidil in Patients With Neurogenic Lower Urinary Tract Dysfunction: An 8-Week, Active-Controlled, Stratified-Randomized, Double-Blind, Double-Dummy, Parallel Group, Noninferiority, Multicenter Design." International neurourology journal, 24(2), 163–171.	Wrong age
Takeda, M., et al. (2011). "Predictive factors for the effect of the α1-D/A adrenoceptor antagonist naftopidil on subjective and objective criteria in patients with neurogenic lower urinary tract dysfunction." BJU International 108(1): 100-107.	Wrong age
Tanaka, M., et al. (2010). "Conversion to silodosin in men on conventional α1-blockers for symptomatic benign prostatic hyperplasia." LUTS: Lower Urinary Tract Symptoms 2(1): 11-15.	Wrong age
Tanaka, Y., et al. (2002). "Urodynamic effects of terazosin treatment for Japanese patients with symptomatic benign prostatic hyperplasia." Journal of Urology 167(6): 2492-2495.	Wrong age
Tang, H. N., et al. (2015). "Terazosin versus alfuzosin in treatment of acute urinary retention in patients with benign prostatic hypertrophy." Hong Kong Journal of Emergency Medicine 22(4): 210-218.	Wrong age

Tinetti, M. E., et al. (2014). "Antihypertensive medications and serious fall injuries in a nationally representative sample of older adults." JAMA Internal Medicine 174(4): 588-595.	Wrong intervention
Turner, A. S., et al. (1975). "Clinical experience with prazosin hydrochloride in arterial hypertension." The New Zealand medical journal 81(535): 240-242.	Wrong study type
Van Kerrebroeck, P., et al. (2000). "Efficacy and safety of a new prolonged release formulation of alfuzosin 10 mg once daily versus alfuzosin 2.5 mg thrice daily and placebo in patients with symptomatic benign prostatic hyperplasia." European Urology 37(3): 306-313.	Wrong age
Van Kerrebroeck, P., et al. (2002). "Long-term safety and efficacy of a once-daily formulation of alfuzosin 10 mg in patients with symptomatic benign prostatic hyperplasia: Open-label extension study." European Urology 41(1): 54-61.	Other
Van Moorselaar, R. J. A., et al. (2005). "Alfuzosin 10 mg once daily improves sexual function in men with lower urinary tract symptoms and concomitant sexual dysfunction." BJU International 95(4): 603-608.	Wrong age
Van Rompay, M. I., et al. (2019). "Impact of 5α-reductase inhibitor and α-blocker therapy for benign prostatic hyperplasia on prostate cancer incidence and mortality." BJU International 123(3): 511-518.	Wrong age
Vestergaard, P., et al. (2011). "Risk of fractures associated with treatment for benign prostate hyperplasia in men." Osteoporosis International 22(2): 731-737.	Wrong age

Wang, J. G. and J. A. Staessen (2003). "Benefits of antihypertensive pharmacologic therapy and blood pressure reduction in outcome trials." Journal of Clinical Hypertension 5(1): 66-75.	Wrong study type
Wang, X., et al. (2014). "Comparative effectiveness of oral drug therapies for lower urinary tract symptoms due to benign prostatic hyperplasia: A systematic review and network meta-Analysis." PLoS ONE 9(9).	Wrong study type
Watanabe, T., et al. (2011). "A randomized crossover study comparing patient preference for tamsulosin and silodosin in patients with lower urinary tract symptoms associated with benign prostatic hyperplasia." Journal of International Medical Research 39(1): 129-142.	Wrong age
Xue, Z., et al. (2007). "Doxazosin gastrointestinal therapeutic system versus tamsulosin for the treatment of benign prostatic hyperplasia: A study in Chinese patients." International Journal of Urology 14(2): 118-122.	Wrong age
Yang, P. S., et al. (2018). "An open-label, prospective interventional study of the tolerability and efficacy of 0.4 mg oral tamsulosin oral controlled absorption system in men with lower urinary tract symptoms associated with benign prostatic hyperplasia who are unsatisfied with treatment with 0.2 mg tamsulosin." Clinical Interventions in Aging 13: 235-242.	Wrong age
Yokoyama, T., et al. (2012). "Comparison of Two Different α1-Adrenoceptor Antagonists, Tamsulosin and Silodosin, in the Treatment of Male Lower Urinary Tract Symptoms Suggestive of Benign Prostatic Hyperplasia: A Prospective Randomized Crossover Study." LUTS: Lower Urinary Tract Symptoms 4(1): 14-18.	Wrong age

Yu, H. J., et al. (2011). "Non-inferiority of silodosin to tamsulosin in treating patients with lower urinary tract symptoms (LUTS) associated with benign prostatic hyperplasia (BPH)." BJU International 108(11): 1843-1848.	Wrong age
Zabkowski, T. and M. Saracyn (2017). "Clinical evaluation of α-1-adrenolytics in patients diagnosed with benign prostatic hyperplasia." Acta Poloniae Pharmaceutica - Drug Research 74(4): 1247-1253.	Wrong age
Zacks, M. (1996). "Terazosin vs finasteride for BPH." The Journal of family practice 43(6): 533.	Wrong study type
Zhang, Z., et al. (2019). "Efficacy and safety of tadalafil 5 mg once-daily in Asian men with both lower urinary tract symptoms associated with benign prostatic hyperplasia and erectile dysfunction: A phase 3, randomized, double-blind, parallel, placebo- and tamsulosin-controlled study." International Journal of Urology 26(2): 192-200.	Wrong age
Zlotta, A. R., et al. (2005). "Evaluation of male sexual function in patients with Lower Urinary Tract Symptoms (LUTS) associated with Benign Prostatic Hyperplasia (BPH) treated with a phytotherapeutic agent (Permixon®), Tamsulosin or Finasteride." European Urology 48(2): 269-276.	Wrong age
Lowe, F. C. (2004). "Role of the newer alpha, -adrenergic-receptor antagonists in the treatment of benign prostatic hyperplasia-related lower urinary tract symptoms." Clinical Therapeutics 26(11): 1701-1713.	Wrong study type
Lukacs, B., et al. (1996). "Safety profile of 3 months' therapy with alfuzosin in 13,389 patients suffering from benign prostatic hypertrophy." European Urology 29(1): 29-35.	Wrong study type

Lukacs, B., et al. (2000). "One-year follow-up of 2829 patients with moderate to severe lower urinary tract symptoms treated with alfuzosin in general practice according to IPSS and a health-related quality-of-life questionnaire." Urology 55(4): 540-546.	Wrong study type
Lukacs, B., et al. (2000). "History of 7093 patients with lower urinary tract symptoms related to benign prostatic hyperplasia treated with alfuzosin in general practice up to 3 years." European Urology 37(2): 183-190.	Wrong age
Lukacs, B., et al. (1996). "Prospective study of men with clinical benign prostatic hyperplasia treated with alfuzosin by general practitioners: 1-Year results." Urology 48(5): 731-740.	Wrong study type
MacDonald, D. and T. A. McNicholas (2003). "Drug treatments for lower urinary tract symptoms secondary to bladder outflow obstruction: Focus on quality of life." Drugs 63(18): 1947-1962.	Wrong study type
MacDonald, R. and T. J. Wilt (2005). "Alfuzosin for treatment of lower urinary tract symptoms compatible with benign prostatic hyperplasia: A systematic review of efficacy and adverse effects." Urology 66(4): 780-788.	Wrong age
Macphee, G. J. A., et al. (1992). "Placebo-controlled trial of doxazosin in management of patients with hypertension and hypercholesterolaemia." Journal of Cardiovascular Pharmacology 20(3): 429-433.	Wrong age
Magoha, G. A. O. (1996). "Medical management of benign prostatic hyperplasia: A review." East African Medical Journal 73(7): 453-456.	Wrong study type

Manjunatha, R., et al. (2016). "A randomized, comparative, open-label study of efficacy and tolerability of alfuzosin, tamsulosin and silodosin in benign prostatic hyperplasia." Indian Journal of Pharmacology 48(2): 134-140.	Wrong age
Mann, R. D., et al. (2000). "The pharmacovigilance of tamsulosin: Event data on 12,484 patients." BJU International 85(4): 446-450.	Wrong age
Marks, L. S., et al. (2003). "First dose efficacy of alfuzosin once daily in men with symptomatic benign prostatic hyperplasia." Urology 62(5): 888-893.	Wrong age
Masumori, N., et al. (2007). "Short-term efficacy and long-term compliance/treatment failure of the α1 blocker naftopidil for patients with lower urinary tract symptoms suggestive of benign prostatic hyperplasia." Scandinavian Journal of Urology and Nephrology 41(5): 422-429.	Wrong age
Masumori, N., et al. (2013). "α1-blocker tamsulosin as initial treatment for patients with benign prostatic hyperplasia: 5-year outcome analysis of a prospective multicenter study." International Journal of Urology 20(4): 421-428.	Wrong age
Masumori, N., et al. (2009). "Ejaculatory disorders caused by alpha-1 blockers for patients with lower urinary tract symptoms suggestive of benign prostatic hyperplasia: Comparison of naftopidil and tamsulosin in a randomized multicenter study." Urologia Internationalis 83(1): 49-54.	Wrong age
Matsumoto, S., et al. (2019). "Influence of alpha-adrenoceptor antagonists therapy on stool form in patients with lower urinary tract symptoms suggestive of benign prostatic hyperplasia." LUTS: Lower Urinary Tract Symptoms.	Wrong age

Matzkin, H., et al. (1992). "Efficacy of terazosin in patients with benign prostatic hyperplasia." European Urology 21(2): 126-130.	Wrong age
Mazzola, C., et al. (1990). "The efficacy and safety of doxazosin in the treatment of mild or moderate essential hypertension when dose adjustment is simplified." Current Therapeutic Research - Clinical and Experimental 48(5): 809-816.	Wrong age
McKiernan, J. M. and F. C. Lowe (1997). "Side effects of terazosin in the treatment of symptomatic benign prostatic hyperplasia." Southern Medical Journal 90(5): 509-523.	Other
McVary, K. T. (2006). "Alfuzosin for symptomatic benign prostatic hyperplasia: Long-term experience." Journal of Urology 175(1): 35-42.	Wrong age
Melo É, A., et al. (2002). "A double-blind, randomized, placebo-controlled study, to assess the efficacy of alfuzosin in the treatment of patients with benign prostatic hyperplasia." Brazilian Journal of Urology 28(1): 25-32.	Wrong age
Michel, M. C., et al. (2001). "A 6-month large-scale study into the safety of tamsulosin." British Journal of Clinical Pharmacology 51(6): 609-614.	Wrong age
Michel, M. C., et al. (1998). "Tamsulosin treatment of 19,365 patients with lower urinary tract symptoms: Does co-morbidity alter tolerability?" Journal of Urology 160(3 I): 784-791.	Wrong age
Mikhailidis, D. P., et al. (1999). "The treatment of hypertension in patients with erectile dysfunction." Current Medical Research and Opinion, Supplement 16(1): s31-s36.	Wrong study type

Milani, S. and B. Djavan (2005). "Lower urinary tract symptoms suggestive of benign prostatic hyperplasia: Latest update on α1-adrenoceptor antagonists." BJU International, Supplement 95(4): 29-36.	Wrong study type
Milicevic, S. and R. Bijelic (2012). "Efficacy and safety of tamsulosin in the treatment of benign prostatic hyperplasia." Medicinski arhiv 66(3): 173-176.	Wrong age
Mori, Y., et al. (2001). "Safety and availability of doxazosin in treating hypertensive patients with chronic renal failure." Hypertension Research 24(4): 359-363.	Wrong age
Narayan, P., et al. (2003). "Long-term safety and efficacy of tamsulosin for the treatment of lower urinary tract symptoms associated with benign prostatic hyperplasia." Journal of Urology 170(2 I): 498-502.	Wrong age
Narayan, P. and H. Lepor (2001). "Long-term, open-label, phase III multicenter study of tamsulosin in benign prostatic hyperplasia." Urology 57(3): 466-470.	Wrong age
Narayan, P., et al. (2005). "Early efficacy of tamsulosin versus terazosin in the treatment of men with benign prostatic hyperplasia: A randomized, open-label trial." Journal of Applied Research 5(2): 237-245.	Wrong age
Nechwatal, W. (1987). "Doxazosin and nitrendipine - A double-blind comparative study in patients with mild to moderate hypertension." Fortschritte der Medizin 105(34): 679-682.	Wrong age
Nguyen, Q. T., et al. (2012). "Managing hypertension in the elderly: A common chronic disease with increasing age." American Health and Drug Benefits 5(3): 146-153.	Wrong study type

Nickel, J. C., et al. (2006). "The beneficial effect of alfuzosin 10 mg once daily in 'real-life' practice on lower urinary tract symptoms (LUTS), quality of life and sexual dysfunction in men with LUTS and painful ejaculation." BJU International 97(6): 1242-1246.	Wrong age
Oelke, M., et al. (2015). "Appropriateness of oral drugs for long-term treatment of lower urinary tract symptoms in older persons: Results of a systematic literature review and international consensus validation process (LUTS-FORTA 2014)." Age and Ageing 44(5): 745-755.	Wrong intervention
Okada, H., et al. (2000). "A comparative study of terazosin and tamsulosin for symptomatic benign prostatic hyperplasia in Japanese patients." BJU International 85(6): 676-681.	Wrong age
Osman, N. I., et al. (2015). "Open-label, 9-month extension study investigating the uro-selective alpha-blocker silodosin in men with LUTS associated with BPH." World Journal of Urology 33(5): 697-706.	Wrong age
Palacio, A., et al. (2004). "Long-term study to assess the efficacy of tamsulosin in the control of symptoms and complications developed in patients with symptomatic benign prostatic hyperplasia (OMNICONTROL study): first-year follow-up report." Archivos Espanoles de Urologia 57(4): 451-460.	Wrong age
Permpongkosol, S., et al. (2011). "Treatment with a Uroselective α1-Blocker Improves Voiding and Sexual Function: A Study in Thai Men with Lower Urinary Tract Symptoms." Journal of Sexual Medicine 8(9): 2582-2589.	Wrong age
Pickering, T. G., et al. (1994). "Differential effects of doxazosin on clinic and ambulatory pressure according to age, gender, and presence of white coat	Other

hypertension. Results of the HALT study." American Journal of Hypertension 7(9 I): 848-852.	
Pogula, V. R., et al. (2019). "Tadalafil vs. tamsulosin in the treatment of lower urinary tract symptoms secondary to benign prostatic hyperplasia: A prospective, randomized study." Central European Journal of Urology 72(1): 44-50.	Wrong age
Pool, J. L. (1996). "Doxazosin: A new approach to hypertension and benign prostatic hyperplasia." British Journal of Clinical Practice 50(3): 154-163.	Wrong study type
Quek, K. F., et al. (2002). "The effects of treating lower urinary tract symptoms on health-related quality of life: a short-term outcome." Singapore medical journal 43(8): 391-398.	Wrong age
Rahardjo, D., et al. (2006). "Efficacy and safety of tamsulosin hydrochloride compared to doxazosin in the treatment of Indonesian patients with lower urinary tract symptoms due to benign prostatic hyperplasia." International Journal of Urology 13(11): 1405-1409.	Wrong age
Resnick, M. I. and C. G. Roehrborn (2007). "Rapid onset of action with alfuzosin 10 mg once daily in men with benign prostatic hyperplasia: A randomized, placebo-controlled trial." Prostate Cancer and Prostatic Diseases 10(2): 155-159.	Wrong age
Rigatti, P., et al. (2003). "A comparison of the efficacy and tolerability of tamsulosin and finasteride in patients with lower urinary tract symptoms suggestive of benign prostatic hyperplasia." Prostate Cancer and Prostatic Diseases 6(4): 315-323.	Wrong age

Roehrborn, C. G. (2001). "Efficacy and safety of once-daily alfuzosin in the treatment of lower urinary tract symptoms and clinical benign prostatic hyperplasia: A randomized, placebo-controlled trial." Urology 58(6): 953-959.	Other
Roehrborn, C. G., et al. (1996). "The Hytrin Community Assessment Trial study: A one-year study of terazosin versus placebo in the treatment of men with symptomatic benign prostatic hyperplasia." Urology 47(2): 159-168.	Wrong age
Roehrborn, C. G. and R. C. Rosen (2008). "Medical therapy options for aging men with benign prostatic hyperplasia: Focus on alfuzosin 10 mg once daily." Clinical Interventions in Aging 3(3): 511-524.	Wrong study type
Roehrborn, C. G., et al. (2003). "Safety and efficacy of alfuzosin 10 mg once-daily in the treatment of lower urinary tract symptoms and clinical benign prostatic hyperplasia: A pooled analysis of three double-blind, placebo-controlled studies." BJU International 92(3): 257-261.	Wrong age
Romero, E., et al. (1992). "Double-blind placebo-controlled trial of terazosin effect on blood pressure and urinary output of dopamine in hypertensive patients." Journal of Clinical Pharmacology 32(9): 816-821.	Wrong age
Rossi, C., et al. (2001). "α-blockade improves symptoms suggestive of bladder outlet obstruction but fails to relieve it." Journal of Urology 165(1): 38-41.	Wrong age
Russo, A., et al. (2014). "Latest pharmacotherapy options for benign prostatic hyperplasia." Expert Opinion on Pharmacotherapy 15(16): 2319-2328.	Wrong study type
Sanchez-Chapado, M. (2000). "The clinical uroselectivity of alfuzosin is not significantly affected by the age of patients with lower urinary tract symptoms suggestive of benign prostatic hyperplasia." BJU International 86(4): 432-438.	Wrong study type

Sánchez-Chapado, M., et al. (2000). "Safety and efficacy of sustained-release alfuzosin on lower urinary tract symptoms suggestive of benign prostatic hyperplasia in 3095 Spanish patients evaluated during general practice." European Urology 37(4): 421-427.	Wrong age
Schimke, L. and J. Schinike (2014). "Urological Implications Of Falls in the Elderly: Lower Urinary Tract Symptoms And Alpha-Blocker Medications." Urologic nursing 34(5): 223-229.	Wrong study type
Schulman, C. C., et al. (1999). "Tamsulosin: 3-year long-term efficacy and safety in patients with lower urinary tract symptoms suggestive of benign prostatic obstruction: Analysis of a European, multinational, multicenter, open-label study." European Urology 36(6): 609-620.	Wrong age
Schulman, C. C., et al. (2001). "Long-term use of tamsulosin to treat lower urinary tract symptoms/benign prostatic hyperplasia." Journal of Urology 166(4): 1358-1363.	Wrong age
Scott, P. J. W., et al. (1988). "A double-blind and cross-over comparison of once daily doxazosin and placebo with steady-state pharmacokinetics in elderly hypertensive patients." European Journal of Clinical Pharmacology 34(2): 119-123.	Other
Setoguchi, M., et al. (1998). "Comparative efficacies of a calcium antagonist and an alpha1 blocker in elderly hypertensive patients with stroke." Clinical and Experimental Hypertension 20(7): 763-774.	Wrong intervention
Shigemura, K., et al. (2012). "Comparison of Naftopidil 75 mg with Tamsulosin Hydrochloride 0.2 mg in the Treatment of Lower Urinary Tract Symptoms with	Wrong age

Benign Prostatic Hyperplasia." LUTS: Lower Urinary Tract Symptoms 4(3): 136-139.	
Sica, D. A. (2001). "Doxazosin revisited." Cardiovascular Reviews and Reports 22(9): 509-516.	Wrong study type
Silke, B., et al. (1992). "Comparison of antihypertensive and lipid actions of terazosin and atenolol in essential hypertension." Journal of Human Hypertension 6(3): 221-225.	Wrong age
Singh, I. et al. (2020). “Efficacy and safety of tadalafil vs tamsulosin in lower urinary tract symptoms (LUTS) as a result of benign prostate hyperplasia (BPH)-open label randomised controlled study.” International journal of clinical practice, 74(8), e13530.	Wrong age
Singh, P., et al. (2013). "Comparison of the efficacy and safety of tamsulosin (0.4 mg) v/s (and)finasteride for short-term treatment of patients with symptomatic benign prostatic hyperplasia." International Journal of Current Pharmaceutical Research 5(1): 24-28.	Wrong age
Singh, P., et al. (2012). "Efficacy and safety of tamsulosin (0.4 mg) once daily for treating symptomatic benign prostatic hyperplasia." Asian Journal of Pharmaceutical and Clinical Research 5(SUPPL.4): 87-91.	Wrong age
Soltero, I., et al. (1988). "A multicenter study of doxazosin in the treatment of severe essential hypertension." American Heart Journal 116(6 II SUPPL.): 1767-1771.	Wrong age
Son, H., et al. (2013). "A retrospective study of clinical outcomes of α-blocker or finasteride monotherapy followed by combination therapy: Determination of	Wrong age

the period of combination therapy of α-blocker and finasteride." International Journal of Clinical Practice 67(4): 351-355.	
Song, K., et al. (2011). "The long-term effect of alfuzosin in patients with lower urinary tract symptoms suggestive of benign prostate hyperplasia: Evaluation of voiding and storage function with respect to bladder outlet obstruction grade and contractility." Urology 77(5): 1177-1182.	Wrong age
Souverein, P. C., et al. (2003). "Use of α-blockers and the risk of hip/femur fractures." Journal of Internal Medicine 254(6): 548-554.	Wrong age
Speakman, M. (2006). "Efficacy and safety of tamsulosin OCAS." BJU International 98(SUPPL. 2): 13-17.	Wrong study type
Srulevich, M. (2009). "Medical management of benign prostatic hypertrophy in older men." Clinical Geriatrics 17(7): 28-32.	Wrong study type
Stokes, G. S. (1988). "Age-related effects of antihypertensive therapy with α-blockers." Journal of Cardiovascular Pharmacology 12(SUPPL. 8): S109-S115.	Wrong study type
Stumpe, K. O., et al. (1995). "Selective α1-receptor blockade by doxazosin. Antihypertensive and metabolic effects in comparison to doxazosin." Munchener Medizinische Wochenschrift 137(41): 650-655.	Wrong age
Su, P. L. et al. (2019). „Alpha-1 Adrenergic-Antagonist Use Increases the Risk of Sleep Apnea: A Nationwide Population-Based Cohort Study." Journal of clinical sleep medicine : JCSM : official publication of the American Academy of Sleep Medicine, 15(11), 1571–1579.	Wrong age

Sun, G. H., et al. (2010). "Efficacy and safety of the doxazosin gastrointestinal therapeutic system for the treatment of benign prostate hyperplasia." Kaohsiung Journal of Medical Sciences 26(10): 532-539.	Wrong age
Sun, X., et al. (2018). "Efficacy and safety of PDE5-Is and α-1 blockers for treating lower ureteric stones or LUTS: A meta-analysis of RCTs." BMC Urology 18(1).	Wrong age
Sun, Y. H., et al. (2011). "Long-term efficacy and safety of tamsulosin hydrochloride for the treatment of lower urinary tract symptoms associated with benign prostatic hyperplasia: Data from china." Chinese Medical Journal 124(1): 56-60.	Wrong age
Sung, S. Y. et al. (2020). „Alpha-1 blocker use increased risk of subsequent renal cell carcinoma: A nationwide population-based study in Taiwan." PloS one, 15(11), e0242429.	Wrong age
Suzuki, H., et al. (2006). "Clinical impact of tamsulosin on generic and symptom-specific quality of life for benign prostatic hyperplasia patients: Using international prostate symptom score and Rand Medical Outcomes Study 36-item Health Survey." International Journal of Urology 13(9): 1202-1206.	Wrong age
Takahashi, H., et al. (2019). "Post-Marketing Surveillance of Silodosin in Patients with Benign Prostatic Hyperplasia and Poor Response to Existing Alpha-1 Blockers: The SPLASH Study." Drugs in R and D 19(1): 47-55.	Wrong study type
Taylor, S. H., et al. (1988). "A comparison of doxazosin and enalapril in the treatment of mild and moderate essential hypertension." American Heart Journal 116(6 II SUPPL.): 1820-1825.	Wrong age

Tsujii, T. (2000). "Comparison of prazosin, terazosin and tamsulosin in the treatment of symptomatic benign prostatic hyperplasia: A short-term open, randomized multicenter study." International Journal of Urology 7(6): 199-205.	Wrong age
Vallancien, G., et al. (2008). "Alfuzosin 10 mg once daily for treating benign prostatic hyperplasia: A 3-year experience in real-life practice." BJU International 101(7): 847-852.	Wrong study type
van der Worp, H. et al. (2019). „Discontinuation of alpha-blocker therapy in men with lower urinary tract symptoms: a systematic review and meta-analysis." BMJ open, 9(11), e030405.	Wrong age
Wagg, A. (2015). "Pharmacological Treatment of Voiding Dysfunction in Older Men." Current Geriatrics Reports 4(1): 44-50.	Wrong study type
Weber, M. (1999). "Management of benign prostatic hyperplasia in patients with hypertension." Cardiovascular Reviews and Reports 20(7): 363-367+374.	Wrong study type
Weinberger, M. H. and A. Fawzy (2000). "Doxazosin in elderly patients with hypertension." International Journal of Clinical Practice 54(3): 181-187,189.	Wrong study type
Wilde, M. I., et al. (1993). "Alfuzosin: A review of its pharmacodynamic and pharmacokinetic properties, and therapeutic potential in benign prostatic hyperplasia." Drugs 45(3): 410-429.	Wrong study type
Wilde, M. I., et al. (1993). "Terazosin. A review of its pharmacodynamic and pharmacokinetic properties, and therapeutic potential in benign prostatic hyperplasia." Drugs & aging 3(3): 258-277.	Wrong study type
Wilt, T. J., et al. (2002). "Terazosin for benign prostatic hyperplasia." Cochrane database of systematic reviews (Online)(4): CD003851.	Wrong study type

Wilt, T. J., et al. (2002). "Terazosin for treating symptomatic benign prostatic obstruction: A systematic review of efficacy and adverse effects." BJU International 89(3): 214-225.	Wrong study type
Wolak, T., et al. (2014). "Doxazosin to treat hypertension: It's time to take it personally - A retrospective analysis of 19495 patients." Journal of Hypertension 32(5): 1132-1137.	Wrong age
Woodard, T. J., et al. (2016). "Management of benign prostatic hyperplasia in older adults." Consultant Pharmacist 31(8): 412-424.	Wrong study type
Wu, Y. J., et al. (2013). "A meta-analysis of efficacy and safety of the new α 1A -adrenoceptor-selective antagonist silodosin for treating lower urinary tract symptoms associated with BPH." Prostate Cancer and Prostatic Diseases 16(1): 78-83.	Wrong study type
Wykretowicz, A., et al. (2008). "Doxazosin in the current treatment of hypertension." Expert Opinion on Pharmacotherapy 9(4): 625-633.	Wrong study type
Yamaguchi, K., et al. (2013). "Silodosin versus naftopidil for the treatment of benign prostatic hyperplasia: A multicenter randomized trial." International Journal of Urology 20(12): 1234-1238.	Wrong age
Yasukawa, K., et al. (2001). "Investigation of the effects of tamsulosin on blood pressure in normotensive, controlled hypertensive, and uncontrolled hypertensive men with benign prostatic hyperplasia." Journal of Health Science 47(2): 192-202.	Other
Yasukawa, K., et al. (2001). "Review of orthostatic tests on the safety of tamsulosin, a selective α1A-adrenergic receptor antagonist, shows lack of	Wrong age

orthostatic hypotensive effects." Journal of International Medical Research 29(3): 236-251.	
Yoshida, M., et al. (2011). "Safety and efficacy of silodosin for the treatment of benign prostatic hyperplasia." Clinical Interventions in Aging 6(1): 161-172.	Wrong study type
Yoshida, M., et al. (2012). "New clinical evidence of silodosin, an α1A selective adrenoceptor antagonist, in the treatment for lower urinary tract symptoms." International Journal of Urology 19(4): 306-316.	Wrong study type
Yoshida, M., et al. (2007). "Effect of tamsulosin hydrochloride on lower urinary tract symptoms and quality of life in patients with benign prostatic hyperplasia evaluation using bother score." Drugs of Today 43(SUPPL. B): 1-7.	Wrong age
Zhang, L., et al. (2016). "Doxazosin oral intake therapy to relieve stent - related urinary symptoms and pain: a prospective, randomized, controlled study." International braz j urol : official journal of the Brazilian Society of Urology 42(4): 727-733.	Wrong age
Zhang, T., et al. (2017). "Clinical evaluation of tamsulosin in the relief of lower urinary tract symptoms in advanced prostate cancer patients." International Urology and Nephrology 49(7): 1111-1117.	Wrong age
Zhang, Y., et al. (2009). "Extended-Release Doxazosin for Treatment of Renal Transplant Recipients With Benign Prostatic Hyperplasia." Transplantation Proceedings 41(9): 3747-3751.	Wrong age
Zhou, Z. et al. (2019). „Meta-Analysis of Efficacy and Safety of Tadalafil Plus Tamsulosin Compared with Tadalafil Alone in Treating Men with Benign	Wrong age

Prostatic Hyperplasia and Erectile Dysfunction." American journal of men's health, 13(5), 1557988319882597.	
Nechwatal, W., et al. (1988). "A double-blind comparative study of doxazosin and nitrendipine in patients with mild-to-moderate essential hypertension." American Heart Journal 116(6 II SUPPL.): 1806-1814.	Wrong age
