# Supplementary material for: Efficacy and safety of adrenergic alpha-1 receptor antagonists in older adults: a systematic review and meta-analysis supporting the development of recommendations to reduce potentially inappropriate prescribing
Source: BMC Geriatr. 2022 Sep 28;22:771. doi: 10.1186/s12877-022-03415-7 (PMC9516834; doi:10.1186/s12877-022-03415-7)
Supplement: Supplementary file 3 — Additional file 3. Summary of characteristics of included studies. [file 12877_2022_3415_MOESM3_ESM.pdf]

Additional file 3 Summary of patient characteristics of included studies

Table S1 Summary of patient characteristics of included interventional studies

**Authors (Year)**	**Recruitment**	**Sample size and age profile**	**Race/ethnicity in %**	**Men in %**	**BPH related scores (mean values)**	**Co-medication**	**Co-morbidities**
ALLHAT (2003) [47][1]	Practice based setting in the US only through (e.g.):<br>· Universities or medical centers<br>· Veterans Hospitals<br>· Practices<br>· Primary care<br>· Specialty clinics	Doxazosin:<br>· n=9,061<br>· ≥70 y: 3,092<br><br>Chlorthalidone:<br>· n=15,255<br>· ≥70 y: 5,410	Chlorthalidone:<br>· White: 47.2%<br>· Black: 31.9%<br>· Hispanic: 15.8%<br>· Other: 5.1%<br><br>Doxazosin:<br>· White: 46.5%<br>· Black: 32.9%<br>· Hispanic: 16%<br>· Other: 4.6%	Chlorthalidone: 53.0%<br><br>Doxazosin: 53.6%	N.a.	Additional treatment for hypertension was allowed with:<br>· Atenolol<br>· Reserpine<br>· Clonidine<br>· Hydralazine	Comorbidities such as atherosclerotic cardiovascular disease, type 2 diabetes and unfavourable cholesterol levels were matched between the chlorthalidone and doxazosin group

Gotoh et al. (2005) [35][2]	Multicenter trial in JP through 17 urologists in 16 sites	Tamsulosin:<br>· n=75<br>· mean age: 68.5 y<br>· 95% CI: 67.0 – 70.1 y<br><br>Naftopidil:<br>· n=69<br>· mean age: 68.0 y<br>· 95% CI: 66.4 – 69.8 y	N.a.	100%	Tamsulosin:<br>· $V_{prostate}$: 33.6 ml<br>· IPSS: 17.1<br>· QoL: 4.4<br>· PVR: 42.5 ml<br><br>Naftopidil:<br>· $V_{prostate}$: 29 ml<br>· IPSS: 15.5<br>· QoL: 4.5<br>· PVR: 46.6 ml	Patients were excluded if they were currently in treatment with:<br>· Antiandrogens<br>· α1-antagonists<br>· Anticholinergic drugs	Patients were excluded if they had (a history of) one or more of the following diseases:<br>· Orthostatic hypertension<br>· Neurological disease incl. bladder dysfunction<br>· Carcinoma of bladder or prostate<br>· Surgery for BPH or bladder neck obstruction<br>· Urinary tract infections
Nishino et al. (2006) [36][3]	Patients of the Department of Urology at Gifu University (JP)	Tamsulosin/ naftopidil:<br>· n=17<br><br>Naftopidil/ tamsulosin:<br>· n=17	N.a.	100%	· $V_{prostate}$: 19.8 ml<br>· IPSS: 20.4<br>· QoL: 4.9<br>· PVR: 54.1 ml<br>· Q_{max}: 9.9 ml/s	Patients were excluded if they had ever been medically treated for BPH	Patients were excluded if they had (a history of) one or more of the following diseases (e.g.):<br>· Neurogenic disorders<br>· Urinary retention<br>· Carcinoma of bladder<br>· Urinary tract infections

Oelke et al. (2014) [48][4]	Patients were recruited internationally in 44 urology sites in Europe (71%), Mexico and Australia	Tamsulosin:<br>· n=168<br>· ≥66 y: 72<br><br>Tadalafil:<br>· n=171<br>· ≥66 y: 75<br><br>Placebo:<br>· n=172<br>· ≥66 y: 77	Tamsulosin:<br>· White: 78%<br>· Black/African American: 0%<br>· American Indian/Alaska Native: 22%<br><br>Tadalafil:<br>· White: 76%<br>· Black/African American: 0.6%<br>· American Indian/Alaska Native: 23.4%<br><br>Placebo:<br>· White: 76.2%<br>· Black/African American: 0%<br>· American Indian/Alaska Native: 23.8%	100%	Tamsulosin:<br>· IPSS: 16.8<br>· Erectile dysfunction (ED): 69%<br>· BMI: 27.9 kg/m²<br><br>Tadalafil:<br>· IPSS: 17.2<br>· ED: 70.8%<br>· BMI: 27.1 kg/m²<br><br>Placebo:<br>· IPSS: 17.4<br>· ED: 69.8%<br>· BMI: 28.1 kg/m²	Previous therapies within 12 mo prior to screening:<br><br>Tamsulosin:<br>· α-blockers: 25.6%<br>· Other LUTS/BPH therapy: 5.4%<br>· ED therapy: 12.5%<br><br>Tadalafil:<br>· α-blockers: 24%<br>· Other LUTS/BPH therapy: 3.5%<br>· ED therapy: 12.3%<br><br>Placebo:<br>· α-blockers: 26.2%<br>· Other LUTS/BPH therapy: 4.7%<br>· ED therapy: 13.4%	Patients excluded if they had (had) prostate cancer
Roehrborn (2006) [49][5]	Patients were recruited internationally in 148 urology sites in	Alfuzosin:<br>· n=759<br>· ≥65 y: 449	N.a.	100%	Alfuzosin:<br>· $V_{prostate}$: 46.9 ml<br>· IPSS: 19.2<br>· PVR: 95.3 ml	Patients were excluded if they were taking medication which would eventually change the voiding pattern	Hypertension:<br>· Alfuzosin: 36.1%<br>· Placebo: 35%

	North America, Europe, Australia, Middle East and South-Africa	Placebo:<br>· n=763<br>· ≥65 y: 439			· Q_{max}: 8.9 ml/s<br><br>Placebo:<br>· $V_{prostate}$: 46.6 ml<br>· IPSS: 19.2<br>· PVR: 89 ml<br>· Q_{max}: 8.8 ml/s		Patients were excluded if they had (a history of) one or more of the following diseases:<br>· Postural hypotension or syncope<br>· Carcinoma of prostate<br>· Surgery of prostate<br>· AUR
Yokoyama et al. (2011) [34][6]	Department of Urology at Kawasaki Medical School, Japan	Tamsulosin:<br>· n=45<br>· mean age: 71.5 y<br><br>Silodosin:<br>· n=45<br>· mean age: 70.2 y<br><br>Naftopidil:<br>· n=46<br>· mean age: 69.1 y	N.a.	100%	Tamsulosin:<br>· $V_{prostate}$: 32.5 ml<br>· IPSS: 18<br>· QoL: 4.49<br>· PVR: 29.7 ml<br>· Q_{max}: 8.56 ml/s<br><br>Silodosin:<br>· $V_{prostate}$: 33.3 ml<br>· IPSS: 18.7<br>· QoL: 4.5<br>· PVR: 57.6 ml<br>· Q_{max}: 9.03 ml/s<br><br>Naftopidil:<br>· $V_{prostate}$: 35 ml<br>· IPSS: 17.4<br>· QoL: 4.55	N.a.	N.a.

					· PVR: 39.1 ml<br>· Q_{max}: 8.63 ml/s		

[1] Patient characteristics refer to total study population including patients of all age groups ≥55 y.

[2] Patient characteristics refer to total study population including patients of all age groups ≥50 y with mean age (95% CI) being 68.5 y (67.0 y – 70.1 y)

[3] All patients are aged ≥66 y.

[4] Patient characteristics refer to total study population including patients of all age groups ≥45 y.

[5] Patient characteristics refer to total study population including patients of all age groups ≥55 y.

[6] Patient characteristics refer to total study population including patients of all age groups ≥50 y with mean age (SD) being 70.2 y (0.9), 71.5 y (1.1) and 69 y (1.2) for the silodosin, tamsulosin or nifedipine group, respectively.

Abbreviations: **BMI** = body mass index, **BPH** = benign prostatic hyperplasia, **ED** = erectile dysfunction, **IPSS =** international prostate symptom score, **N.a. =** not available, **PVR =** post-void residual urine, $\mathbf{Q_{max}}$ **=** maximum urinary flow rate, **QoL =** quality of life**, y** = years

Table S2 Summary of patient characteristics of included observational studies

Authors (Year)	Recruitment	Sample size and age profile	Race/ethnicity in %	Men in %	Co-medication	Co-morbidities
**Retrospective Cohort Studies:**						
Chrischilles et al. (2001) [50][1]	Cohorts were created from information received through a medical claims database in the US (1995-1997) including:<br>· Outpatient drug utilization<br>· Inpatient physician services<br>· Outpatient physician services	Users:<br>· n=1,564<br>· Mean age: 73 y<br>· Prazosin=15<br>· Doxazosin=782<br>· Terazosin=839<br><br>Non-Users:<br>· n=8,641<br>· Mean age: 72.5 y	N.a.	100%	Use of additional antihypertensive drugs (α1-blocker users vs. non-users):<br>· Any agent: 56.3 % vs. 26.2%<br>· ACE-inhibitors: 28.9% vs. 12.2%<br>· Beta-blockers: 15.4% vs. 6.6%<br>· Ca-Channel-blockers: 35.6% vs. 14.7%<br>· Diuretics: 33.7% vs. 13.4%<br><br>No. of agents used:<br>· 0: 34.1% vs. 71.4%<br>· 1: 30.2% vs. 14.3%<br>· 2: 21.4% vs. 9.8%<br>· >3: 14.2% vs. 4.6%	α1-blocker users vs. non-users:<br>· Hypertension: 23% vs. 20.4%<br>· Type 2 Diabetes: 8.2% vs. 7.1%<br>· Cardiac arrhythmia: 8.3% vs. 7.2%<br><br>No. of comorbidities:<br>· 0: 62.7% vs. 66.3%<br>· 1: 28.8% vs. 27.6%<br>· 2: 7.4% vs. 5.0%<br>· >3: 1.2% vs. 1.1%

Duan et al. (2018) [33][2]	Cohorts were created from US Medicare data (2006-2012) including 100% US Medicare beneficiaries	Tamsulosin:<br>· n=253,136<br><br>No BPH-medication:<br>· n=180,926<br><br>Doxazosin:<br>· n=28,581<br><br>Terazosin:<br>· n=23,858<br><br>Alfuzosin:<br>· n=17,934<br><br>Dutasteride:<br>· n=34,027<br><br>Finasteride:<br>· n=38,767	Tamsulosin:<br>· White: 86.7%<br>· Black: 5.7%<br>· Hispanic: 2.6%<br>· Other: 5.1%<br><br>No BPH medication:<br>· White: 86.8%<br>· Black: 5.6%<br>· Hispanic: 2.5%<br>· Other: 5.1%	100%	No. of drugs used (tamsulosin vs. no BPH medication):<br>· 1-2: 29.2% vs. 29.2%<br>· 3-4: 31.3% vs. 31.3.%<br>· 5-6: 18.2% vs. 18.4%<br>· ≥7: 8.7% vs. 8.6%	Tamsulosin vs. no BPH medication:<br>· CeVD: 7.7% vs. 7.7%<br>· PVD: 10.9% vs. 10.9%<br>· CHF: 10.3% vs. 10.1%<br>· Hypertension: 64.4% vs. 64.3%<br>· Diabetes: 26.7% vs. 26.7%<br>· Hyperlipidemia: 56.9% vs. 56.7%<br>· Depression: 4.9% vs. 4.8%

Hiremath et al. (2019) [54][3]	Cohorts were derived from medical and administrative databases of Ontario, Canada (April 1st 1997 – March 31st 2015)	Matched cohorts:<br>Alpha-1 antagonist users (terazosin, prazosin, doxazosin):<br>· n=14,106<br>· Mean age: 75.7 y<br><br>Other BP-lowering medication users:<br>· n=14,106<br>· Mean age: 75.7 y	N.a.	0%, 100% women	High dimensional propensity score matching (limit ≥10%) between cohorts (α1-blocker users vs. other BP-lowering medication) with regard to :<br>· Statins: 42.7% vs. 40.1%<br>· Beta-blockers: 42.7% vs. 57.6%<br>· ACE-inhibitors: 45.3% vs. 56.3%<br>· Thiazide diuretics: 32% vs. 52.3%<br>· Loop diuretics: 17.8% vs. 8.6%<br>· Ca-channel-blockers: 65.3% vs. 65.8%<br>· Antiarrhythmics: 1.1% vs. 0.9%<br>· Clopidogrel: 3.1% vs. 2.6%<br>· Antidiabetic medication: 28.4% vs. 26.8%<br>· Antipsychotics: 3.1% vs. 2.7%<br>· Nitrates: 6.6% vs. 6.6%<br>· Clonidine: 1.3% vs. 0.5%	High dimensional propensity score matching (limit ≥10%) between cohorts (α1-blocker users vs. other BP-lowering medication) with regard to (e.g.):<br>· Diabetes mell.: 41.1% vs. 40.0%<br>· Ischemic stroke: 3.6% vs. 3.4%<br>· Myocardial infarction: 1.4% vs. 1.4%<br>· Cong. Heart failure: 8.9% vs. 6.8%<br>· CAD: 16.5% vs. 16.7%<br>· PVD: 3.2% vs. 2.5%<br>· COPD: 4.5% vs. 3.7%<br>· Arrhythmia: 6.4% vs. 6.1%<br>· Major cancer: 9.9% vs. 9.5%<br>· Fall: 8.8% vs. 8.4%<br>· Facture: 7.6% vs. 7.4%
Hundemer et al. (2021) [32][3]	Cohorts were derived from medical and administrative databases of Ontario, Canada (2007 – 2015)	Matched cohorts:<br>Alpha-1 antagonist users (terazosin, prazosin, doxazosin):<br>· n=16,088<br>· Mean age: 75 y<br><br>Other BP-lowering medication users:	N.a.	α1-blocker users:<br>· 63%<br><br>Other:<br>· 62%	High dimensional propensity score matching (limit ≥10%) between cohorts (α1-blocker users vs. other BP-lowering medication) with regard to:<br>No. of BP-lowering medications:<br>· 1: 3% vs. 3%<br>· 2: 11% vs. 11%<br>· 3: 20% vs. 20%<br>· 4: 31% vs. 31%	High dimensional propensity score matching (limit ≥10%) between cohorts (α1-blocker users vs. other BP-lowering medication) with regard to (e.g.):<br>· Diabetes mell.: 55% vs. 55%<br>· Ischemic stroke: 4% vs. 4%<br>· Myocardial infarction: 5% vs. 7%<br>· Cong. Heart failure: 16% vs. 19%<br>· CAD: 32% vs. 34%<br>· PVD: 3% vs. 3%

		· n=16,088<br>· Mean age: 75 y			· 5: 36% vs. 36%<br><br>Other medication:<br>· ARB: 32% vs. 28%<br>· Ca-channel-blockers: 56% vs. 56%<br>· Beta-blockers: 40% vs. 40%<br>· Thiazide diuretics: 23% vs. 23%<br>· Loop diuretics: 20% vs. 19%<br>· Nitrate: 8% va. 11%<br>· Clonidine: 1% vs. 0%<br>· Antiarrhythmic: 2% vs. 2%<br>· Clopidogrel: 65% vs. 66%<br>· Statin: 65% vs. 66%<br>· Antidiabetic medication: 40% vs. 40%<br>· Antipsychotics: 4% vs. 3%	· COPD: 7% vs. 6%<br>· Arrhythmia: 13% vs. 15%<br>· Major cancer: 13% vs. 14%
Siemens et al. (2021) [55][3]	Cohorts were derived from medical and administrative databases of Ontario, Canada (2005 – 2015)	No medication:<br>· n=69,988<br>· Mean age: 74 y<br><br>Alpha-1 antagonist:<br>· n=55,383<br>· Mean age: 74 y<br><br>5-ARI + alpha-1 antagonist:	N.a.	100%	N.a.	Baseline characteristics of cohorts (no medication / α1-blocker only / α1-blocker + 5-ARI combination):<br>· Diabetes mell.: 9%/9%/9%<br>· CVD: 3%/3%/3%<br>· Myocardial infarction: 3%/4%/3%<br>· Cong. Heart failure: 0%/0%/0%<br>· PVD: 2%/2%/2%/<br>· COPD: 3%/3%/3%/<br>· Primary cancer: 5%/4%/3%

		· n=41,491<br>· Mean age: 74 y				
Tae et al. (2019) [56][3]	Cohorts were derived from the national health claims database of the Republic of Korea (January 1st, 2011 – December 31st, 2011)	Cohorts prior to matching:<br>No medication:<br>· n=3,336<br>· Mean age: 77 y<br><br>Tamsulosin:<br>· n=33,568<br>· Mean age: 76 y<br><br>Doxazosin:<br>· n=7,012<br>· Mean age: 77 y<br><br>Terazosin:<br>· n=9,443<br>· Mean age: 77 y	N.a.	100%	Prospensity score matching between cohorts with regard to the following therapies:<br>· Antiplatelet<br>· Anticoagulant<br>· Statin<br>· 5-ARI	Prospensity score matching between cohorts with regard to the following diagnoses:<br>· Hypertension<br>· Diabetes mellitus<br>· Prior cancer history<br>· Myocardial infaction<br>· CHF<br>· PVD<br>· Renal disease

		Alfuzosin:<br>· n=5,904<br>· Mean age: 76 y				
Welk et al. (2015) [51][3]	Cohorts were derived from administrative data provided by the province of Ontario, Canada	α1-blocker initiation:<br>· n=147,084<br><br>No initiation:<br>· n=147,084	n.a.	100%	Matched cohorts: unexposed (no α1-blocker use) vs. exposed (α1-blocker use):<br>· Cancer: 22% vs. 20.5%<br>· Cataract: 19.3% vs. 19%<br>· CKD: 9.8% vs. 9.1%<br>· Chronic lung disease: 28.1% vs. 28.1%<br>· CHF: 14.1% vs. 13.3%<br>· Coronary artery disease or angina: 42.9% vs. 42.4%<br>· Dementia: 9.9% vs. 10.5%<br>· Diabetes: 20.6% vs. 20.9%<br>· Glaucoma: 6.8% vs. 6.5%<br>· Hypertension: 69.8% vs. 69.4%<br>· Osteoporosis: 6.1% vs. 6.0%<br>· Prostate cancer: 13.0% vs. 11.7%	Matched cohorts: unexposed (no α1-blocker use) vs. exposed (α1-blocker use):<br>· 5α-reductase inhibitors: 7.4% vs. 7.4%<br>· ACE-inhibitors: 49.5% vs. 49.3%<br>· Anti-inflammatory drugs: 15.8% vs. 16.8%<br>· Antibiotics: 37.3% vs. 38.3%<br>· Anticonvulsants: 5.0% vs. 5.1%<br>· Antidepressants: 7.2% vs. 7.4%<br>· Antineoplastic: 5.5% vs. 5.0%<br>· Antiplatelets: 7.3% vs. 7.2%<br>· Benzodiazepines: 14.2% vs. 14.4%<br>· Beta-blockers: 32.3% vs. 30.9%<br>· Bisphosphonates: 6.8% vs. 6.5%<br>· Ca-channel blockers: 26.9% vs. 27.3%<br>· Glucocorticoids: 9.6% vs. 9.5%<br>· Inhaled acetylcholine: 7.5% vs. 7.6%

						· Inhaled beta-agonist: 13.3% vs. 12.8%<br>· Inhaled corticosteroids: 6.0% vs. 5.5%<br>· Narcotics: 19.2% vs. 19.4%<br>· Non-potassium sparing diuretics: 26.4% vs. 23.8%<br>· Potassium sparing diuretics: 4.2% vs. 3.6%<br>· PPI: 25.9% vs. 25.4%<br>· SSRI: 7.0% vs. 7.0%<br>· Statins: 48.8% vs. 48.6%
**Case-Control Studies:**						
Hall and McMahon (2007) [52][4]	Cases and Controls were derived from data from the UK primary care records (THIN database)	Cases (fracture):<br>· n=6,540<br>· Taking MR Doxazosin: 66<br>· Taking MR Doxazosin and ≥75y: 32<br><br>Controls (no fracture):<br>· n=26,495<br>· Taking MR Doxazosin: 311	N.a.	100%	Cases (fractures) vs. controls (no fractures):<br>· Thiazide diuretics: 14.5% vs. 17.1%<br>· Other anti-hypertensives: 31.4% vs. 33.5%<br>· Other cardiac drugs: 22.1% vs. 19.9%<br>· Benzodiazepines: 9.8% vs. 7.3%<br>· Antipsychotics: 6.2% vs. 3.6%<br>· NSAIDs: 28.8% vs. 25.3%<br>· Antidepressants: 16.1% vs. 10.3%<br>· Oestrogen: 3.8% vs. 5.9%<br>· Other osteoporosis treatment: 12.3% vs. 7.7%	Cases (fractures) vs. controls (no fractures):<br>· Arthritis (excl. rheumatoid arthritis): 27.6% vs. 26.8%<br>· Heart failure: 6.9% vs. 5.5%<br>· COPD: 20.2% vs. 27.6%<br>· Cerebrovascular accident: 10.9% vs. 8.6%<br>· Osteoporosis: 8.0% vs. 4.8%<br>· Type II diabetes: 8.9% vs. 7.9%<br>· Dementia: 5.4% vs. 2.2%

		· Taking MR Doxazosin and ≥75y: 173			· Glucocorticoids: 8.4% vs. 6.0%	
Testa et al. (2018) [53][5]	Cases and Controls were enrolled in different settings in Italy including outpatient departments, nursing homes and acute care units	Syncopal fall:<br>· n=354<br>· Mean age: 83.3 y<br><br>Non-syncopal fall:<br>· n=168<br>· Mean age: 83.9 y	N.a.	37.9%	Cases (syncopal fall) vs. controls (non-syncopal fall):<br>· No. of antihypertensives (mean): 2.9 vs. 2.5<br>· < 2 antihypertensives: 50% vs. 56.4%	Cases (syncopal fall) vs. controls (non-syncopal fall):<br>· Alzheimer's: 31.9% vs. 35.1%<br>· Vascular dementia: 42.9% vs. 38.7%<br>· Mixed dementia: 15.5% vs. 15.5%<br>· Parkinson's: 5.6% vs. 6.5%<br>· Hypertension: 74.3% vs. 75%<br>· CAD: 19.5% vs. 18.5%<br>· CHF: 8.5% vs. 10.1%<br>· Atrial fibrillation: 25.1% vs. 23.8%<br>· Stroke: 11.6% vs. 19.6%<br>· TIA: 7.6% vs. 7.1%<br>· Carotid atherosclerosis: 27.4% vs. 20.2%<br>· Psychiatric disease: 33.6% vs. 29.2%<br>· Diabetes: 20.9% vs. 24.4%<br>· Dysthyroidism: 10.9% vs. 10.9%

[1] All patients are aged ≥65 y.

[2] Duan (2018) includes 6 cohort-pairs, all of which were prospensity-score-matched. The figures presented in this table only concern the biggest cohort with 161,729 people comparing tamsulosin-users vs. no BPH medication. All patients are aged ≥66 y.

[3] All patients are aged ≥66 y.

[4] Patient characteristics refer to total study population including patients of all age groups ≥50 y.

[5] All patients are aged ≥65 y.

Abbreviations: **5-ARI** = 5-alpha reductase inhibitor, **ARB** = angiotensin II receptor blockers, **CAD** = coronary artery disease, **COPD** = chronic obstructive pulmonary disease, **CVD** = cerebrovascular disease, **N.a.** = not available, **PVD** = peripheral vascular disease

Table S3 Summary of patient characteristics of included meta-analyses

Authors (Year)	Data used	Patients	Race/ethnicity in %	Men in %	BPH related scores (mean values)	Co-medication	Co-morbidities
Buzelin et al. (1997) [57][1]	Meta-analysis using raw data from two placebo-controlled studies conducted in 61 urological centers throughout Europe [56], 2nd study not published separately	SR alfzusosin:<br>· n=292<br>· ≥65 y: 149<br><br>Placebo:<br>· n=296<br>· ≥65 y: 153	N.a.	100%	Alfuzosin:<br>· Boyarsky score: 9.4<br>· Q_{max}: 9.3 ml/s<br><br>Placebo:<br>· Boyarsky score: 9.6<br>· Q_{max}: 9.2 ml/s	Alfuzosin:<br>· Antihypertensives: 30%<br><br>Placebo:<br>· Antihypertensives: 30%	Alfuzosin:<br>· CVD: 43%<br>· Hypertension: 29%<br><br>Placebo:<br>· CVD: 43%<br>· Hypertension: 29%
Lowe (1994) [58][2]	Meta-analysis using raw data from six placebo-controlled trials, three conducted in the US (two of which two are unpublished) and three conducted in Europe [57-60]	Terazosin:<br>· n=636<br>· ≥65 y: 285<br><br>Placebo:<br>· n=360<br>· ≥65 y: 162	White: 94%	100%	N.a.	N.a.	N.a.

Chapple et al. (1997) [59]	Retrospective analysis of data from a meta-analysis [61] using raw data from two European multinational, multicentre, double-blind, placebo-controlled, randomized trials [62], 2nd study not published separately	Tamsulosin:<br>· <65 y: 190<br>· ≥65 y: 191<br><br>Placebo:<br>· <65 y: 93<br>· ≥65 y: 100	N.a.	100%	N.a.	Tamsulosin:<br>· Antihypertensives/CV medication: 55/191 (29%)<br><br>Placebo:<br>· Antihypertensives/CV medication: 25/100 (25%)	Tamsulosin:<br>· CVD: 69/191 (39%)<br>· Hypertension: 46/186 (25%)<br><br>Placebo:<br>· CVD: 23/100 (23%)<br>Hypertension: 22/97 (23%)

[1] Patient characteristics refer to total study population including patients of all age groups.

[2] Patient characteristics refer to total study population including patients of all age groups.

Abbreviations: **CV(D)** = cardiovascular (disease), **N.a.** = not available, **Q_{max} =** maximum urinary flow rate, **y** = years
