# Supplementary material for: Efficacy and safety of adrenergic alpha-1 receptor antagonists in older adults: a systematic review and meta-analysis supporting the development of recommendations to reduce potentially inappropriate prescribing
Source: BMC Geriatr. 2022 Sep 28;22:771. doi: 10.1186/s12877-022-03415-7 (PMC9516834; doi:10.1186/s12877-022-03415-7)
Supplement: Supplementary file 4 — Additional file 4. Summary of patient characteristics of included studies. [file 12877_2022_3415_MOESM4_ESM.pdf]

Additional file 4 Additional information on patient characteristics for each study used in the included meta-analyses

Meta-analysis	Included studies	Race/ethnicity in %	Men in %	BPH related scores (mean values)	Co-medication	Co-morbidities
Buzelin et al. (1997) [57]	Buzelin et al. (1997) [60]<br><br>Double-blind randomized placebo-controlled trial	N.a.	100%	SR Alfuzosin:<br>· IPSS: 15.0<br>· Boyarsky score: 9.9<br>· Q_{max}: 10.4 ml/s<br>· PVR: 58 ml<br><br>Placebo:<br>· IPSS: 15.9<br>· Boyarsky score: 10.3<br>· Q_{max}: 10.1 ml/s<br>· PVR: 63 ml	Patients were excluded if they used a drug that could interfere with the voiding pattern.	SR Alfuzosin:<br>· CVD: 45%<br>· Hypertension: 29%<br><br>Placebo:<br>· CVD: 49%<br>· Hypertension: 29%
	Second included study not published and data therefore not available.					
Lowe (1994) [58]	Lepor et al. (1992) [61]<br><br>Double-blind randomized placebo-controlled trial	· N.a.	100%	Placebo/2mg/5mg/10mg:<br><br>· Boyarsky score: 9.7/10.0/10.7/10.1<br>· Q_{max} in ml/s: 10.1/8.8/9.3/8.8	Patients were excluded if they used a drug that could interfere with study (e.g. α-agonists, (anti)cholinergic drug, diuretics, antihypertensive medication)	Patients were excluded if they had (a history of) one or more of the following diseases:<br>· Postural hypotension<br>· TIA<br>· Myocardial infarction<br>· CeVD

						· Carcinoma of prostate<br>· Surgery for BPH<br>· Urinary tract disease<br>· Insulin dependent diabetes<br>· Renal insufficiency<br>· Hepatic impairment
	Lloyd et al. (1992) [62]<br><br>Double-blind randomized placebo-controlled trial	N.a.	100%	Placebo/2mg/5mg/10mg:<br><br>· Boyarsky obstructive Symptom Score: 6.8/6.5/6.3/6.6<br>· Boyarsky irritative Symptom Score: 4.4/4.0/4.1/5.2<br>· Q_{max} in ml/s: 7.8/7.9/8.0/8.4	Patients were excluded if they used a drug that could interfere with study (e.g. antihypertensive medication incl. diuretics and vasodilators)	Patients were excluded if they had (a history of) one or more of the following diseases:<br>· Postural hypotension<br>· Syncope<br>· TIA<br>· Myocardial infarction<br>· CeVD<br>· Carcinoma of prostate<br>· Surgery for BPH<br>· Urinary tract disease<br>· Hydronephrosis<br>· Insulin dependent diabetes<br>· Renal insufficiency<br>· Hepatic impairment

	Di Silverio (1992) [63]<br><br>Double-blind randomized placebo-controlled trial	N.a.	100%	Placebo/2mg/5mg/10mg:<br><br>· Boyarsky obstructive Symptom Score: 6.0/5.1/6.0/6.0<br>· PVR in ml: 48.8/45.6/47.2/60.4<br>· Q_{max} in ml/s: 8.8/8.8/8.3/8.4	Patients were excluded if they were treated for hypertension	N.a.
	Brawer et al. (1993) [64]<br><br>Double-blind randomized placebo-controlled trial	N.a.	100%	Placebo vs. terazosin:<br><br>· Boyarsky symptom score: 10.4 vs. 10.9<br>· Q_{max} in ml/s: 8.8 vs. 8.6	N.a.	Patients were excluded if they had (a history of) one or more of the following diseases:<br>· Carcinoma of prostate<br>· Detrusor instability<br>· Significant cardiopulmonary disease
	2 included studies not published and data therefore not available.					
