# Supplementary material for: Efficacy and safety of adrenergic alpha-1 receptor antagonists in older adults: a systematic review and meta-analysis supporting the development of recommendations to reduce potentially inappropriate prescribing
Source: BMC Geriatr. 2022 Sep 28;22:771. doi: 10.1186/s12877-022-03415-7 (PMC9516834; doi:10.1186/s12877-022-03415-7)
Supplement: Supplementary file 5 — Additional file 5. Additional information on patient characteristics for each study used in the included meta-analyses. [file 12877_2022_3415_MOESM5_ESM.pdf]

Additional file 5 Detailed summary of study findings of included studies

Table S1 Detailed summary of study findings of included interventional studies

**Authors (Year)** **Follow-up period**	**Drug vs. comparator**	**Outcome**	**Relative Risk (RR)/Odds Ratio (OR)/Hazard Ratio (HR)**	**Mean difference (95% CI)**	**Events (%) or mean score (SD)**	**p-value**
ALLHAT (2003) [47] Follow-up: mean: 3.2 years	Doxazosin vs. chlorthalidone	Combined CVD <65 y	RR: 1.15 (1.04-1.27)			<0.05
	Doxazosin vs. chlorthalidone	Combined CVD ≥65 y	RR: 1.23 (1.14-1.32)			<0.05
	Doxazosin vs. chlorthalidone	Heart failure <65 y	RR: 1.76 (1.40-2.22)			<0.05
	Doxazosin vs. chlorthalidone	Heart failure ≥65 y	RR: 1.89 (1.65-2.17)			<0.05
Gotoh et al. (2005) [35] Follow-up: 12 weeks	Tamsulosin 0.2 mg intragroup	Change in IPSS pre vs. post administration		-8.4 (-10, -6.8)		<0.001
	Naftopidil 50 mg intragroup	Change in IPSS pre vs. post administration		-5.9 (-7.3, -4.5)		<0.001
	Tamsulosin 0.2 mg vs. naftopidil 50 mg	Change in IPSS pre vs. post administration		-8.4 (-10, -6.8) vs. -5.9 (-7.3, -4.5)		0.060

	Tamsulosin 0.2 mg intragroup	Change in QoL-score pre vs. post administration		-1.4 (-1.7, -1.1)		<0.001
	Naftopidil 50 mg intragroup	Change in QoL-score pre vs. post administration		-1.3 (-1.7, -1.0)		<0.001
	Tamsulosin 0.2 mg vs. naftopidil 50 mg	Change in QoL-score pre vs. post administration		-1.4 (-1.7, -1.1) vs. -1.3 (-1.7, -1.0)		0.801
	Tamsulosin 0.2 mg vs. naftopidil 50 mg	ADEs			9/95 (9.5%) vs. 9/90 (10%)	0.94
Nishino et al. (2006) [36]<br><br>Follow-up:<br>9 weeks (2 x 4-week trials / substance; 1-week washout in between)	Tamsulosin 0.2 mg intragroup	IPSS pre- vs. post-administration			20.4 (3.5) vs. 9.3 (3.0)	<0.001
	Naftopidil 50 mg intragroup	IPSS pre- vs. post-administration			20.4 (3.5) vs. 8.9 (3.2)	<0.001
	Tamsulosin 0.2 mg vs. naftopidil 50 mg	Change in IPSS pre- to post-administration between substances			20.4 (3.5) to 9.3 (3.0) vs. 20.4 (3.5) to 8.9 (3.2)	0.265
	Tamsulosin 0.2 mg intragroup	QoL-score pre- vs. post-administration			4.9 (0.7) vs. 2.7 (1.1)	<0.001
	Naftopidil 50 mg intragroup	QoL-score pre- vs. post-administration			4.9 (0.7) vs. 2.6 (1.1)	<0.001

	Tamsulosin 0.2 mg vs. naftopidil 50 mg	Change in QoL-score pre- to post-administration between substances			4.9 (0.7) to 2.7 (1.1) vs. 4.9 (0.7) to 2.6 (1.1)	0.201
	Naftopidil 50 mg vs. tamsulosin 0.2 mg	ADEs			0/34 (0%) vs. 0/34 (0%)	>0.05
	Tamsulosin 0.2 mg vs. naftopidil 50 mg	Withdrawals			0/34 (0%) vs. 0/34 (0%)	>0.05
Oelke et al. (2014) [48]<br><br>Follow-up: 12 weeks	Tamsulosin 0.4 mg vs. placebo	TSS-BPH ≤65 y			28.8 (16.9) vs. 31.2 (17.3)	0.212
	Tamsulosin 0.4 mg vs. placebo	TSS-BPH >65 y			32.4 (15.8) vs. 32.2 (17.9)	0.759
	Tadalafil 5 mg vs. placebo	TSS-BPH ≤65 y			25.2 (17.8) vs. 31.2 (17.3)	0.013
	Tadalafil 5 mg vs. placebo	TSS-BPH >65 y			29.0 (17.6) vs. 32.2 (17.9)	0.184
Roehrborn (2006) [49]<br><br>Follow-up: 24 months	Alfuzosin 10 mg vs. placebo	Worsening of IPSS by ≥4 within 2 years treatment ≥65 y	RR: 0.84 (0.62-1.15)*		62/443 (14%) vs. 72/433 (16.6%)	>0.05
	Alfuzosin 10 mg vs. placebo	Occurrence of AUR within 2 years treatment ≥65 y	RR: 0.98 (0.39-2.44)*		9/443 (2%) vs. 9/433 (2,1%)	>0.05

	Alfuzosin 10 mg vs. placebo	Need for BPH-related surgery within 2 years treatment ≥65 y	RR: 0.64 (0.36-1.12)*		19/443 (4.3%) vs. 29/433 (6.7%)	>0.05
Yokoyama et al. (2011) [34]<br><br>Follow-up: 12 weeks	Silodosin 8 mg intragroup	IPSS pre- vs. post-administration			18.7 (0.7) vs. 13.8 (1.2)	<0.001
	Tamsulosin 0.2 mg intragroup	IPSS pre- vs. post-administration			18.0 (1.1) vs. 10.7 (1.4)	<0.001
	Naftopidil 50 mg intragroup	IPSS pre- vs. post-administration			17.4 (0.8) vs. 11.3 (1.1)	<0.001
	Silodosin 8 mg vs. tamsulosin 0.2 mg vs. naftopidil 50 mg	Change in IPSS pre- vs. post-administration between substances			18.7 (0.7) to 13.8 (1.2) vs. 18.0 (1.1) to 10.7 (1.4) vs. 17.4 (0.8) to 11.3 (1.1)	>0.05
	Silodosin 8 mg intragroup	QoL-score pre- vs. post-administration			4.5 (0.1) vs. 3.4 (0.2)	<0.001
	Tamsulosin 0.2 mg intragroup	QoL-score pre- vs. post-administration			4.5 (0.1) vs. 2.7 (0.3)	<0.001
	Naftopidil 50 mg intragroup	QoL-score pre- vs. post-administration			4.5 (0.1) vs. 3.1 (0.2)	<0.001
	Silodosin 8 mg vs. tamsulosin 0.2 mg	Change in QoL-score pre- to post-			4.5 (0.1) to 3.4 (0.2) vs.	>0.05

	vs. naftopidil 50 mg	administration between substances			4.5 (0.1) to 2.7 (0.3) vs. 4.5 (0.1) to 3.1 (0.2)	
	Silodosin 8 mg vs. tamsulosin 0.2 mg vs. naftopidil	Withdrawals due to ADEs			4/41 (9,8%) vs. 1/39 (2,6%) vs. 1/42 (2,4%)	
	Silodosin 8 mg vs. tamsulosin0.2 mg vs. naftopidil	Abnormal ejaculation after 4-week treatment			10/11 (90,9%) vs. 1/12 (8,3%) vs. 1/15 (6,7%)	
	Silodosin 8 mg vs. tamsulosin 0.2 mg vs. naftopidil	Abnormal ejaculation after 12-week treatment			8/10 (80%) vs. 1/5 (20%) vs. 1/14 (7,1%)	

* Results calculated based on the figures provided in the original paper

Table S2 Detailed summary of study findings of included observational studies

**Authors (Year)**<br>**Follow-up period**	**Drug vs. comparator**	**Outcome**	**Relative Risk (RR)/Odds Ratio (OR)/Hazard Ratio (HR)**	**Mean difference (95% CI)**	**Events (%) or mean score (SD)**	**p-value**
**Retrospective Cohort Studies:**						
Chrischilles et al. (2001) [50]<br><br>Follow-up:<br>4 months	α1-blocker treatment vs. no α1-blocker treatment	Compare no. of ADEs/10,000 person-days<br>4 months pre- to 4 months post initiation			2.82 to 4.64 vs. 3.62 to 3.60	0.001
	α1-blocker treatment vs. no α1-blocker treatment	Compare no. of ADEs/10,000 person-days<br>3 months pre- to 3 months post initiation			2.99 to 5.03 vs. 3.88 to 3.56	<0.001
	α1-blocker treatment vs. no α1-blocker treatment	Compare no. of ADEs/10,000 person-days<br>2 months pre- to 2 months post initiation			3.53 to 5.89 vs. 3.89 to 3.72	<0.001
	α1-blocker treatment vs. no α1-blocker treatment	Compare no. of ADEs/10,000 person-days			4.72 to 7.07 vs. 4.04 to 3.69	0.001

		1 month pre- to 1 month post initiation				
	Concomitant antihypertensives: α1-blocker treatment vs. no α1-blocker treatment	Compare no. of ADEs/10,000 person-days 4 months pre- to 4 months post initiation			4.21 to 5.15 vs. 3.10 to 3.79	<0.006
	Concomitant antihypertensives: α1-blocker treatment vs. no α1-blocker treatment	Compare no. of ADEs/10,000 person-days 3 months pre- to 3 months post initiation			4.77 to 5.63 vs. 3.10 to 3.67	0.003
	Concomitant antihypertensives: α1-blocker treatment vs. no α1-blocker treatment	Compare no. of ADEs/10,000 person-days 2 months pre- to 2 months post initiation			5.87 to 6.60 vs. 3.62 to 4.07	0.511
	Concomitant antihypertensives: α1-blocker treatment vs. no α1-blocker treatment	Compare no. of ADEs/10,000 person-days 1 month pre- to 1 month post initiation			5.50 to 6.60 vs. 3.33 to 4.00	0.442
Duan et al. (2018) [33]	Tamsulosin vs. no BPH medication	Incidence of dementia/1,000 person-years	HR: 1.17 (1.14-1.21)		31.3 vs. 25.9	<0.001

Follow-up: Median: 19.8 months	Tamsulosin vs. doxazosin	Incidence of dementia/1,000 person-years	HR: 1.20 (1.12-1.28)		32.7 vs. 27.5	<0.001
	Tamsulosin vs. terazosin	Incidence of dementia/1,000 person-years	HR: 1.11 (1.04-1.19)		37.1 vs. 32.7	0.002
	Tamsulosin vs. alfuzosin	Incidence of dementia/1,000 person-years	HR: 1.12 (1.03-1.22)		30.4 vs. 28.4	0.010
	Tamsulosin vs. dutasteride	Incidence of dementia/1,000 person-years	HR: 1.26 (1.19-1.34)		32.7 vs. 26.5	<0.001
	Tamsulosin vs. finasteride	Incidence of dementia/1,000 person-years	HR: 1.13 (1.07-1.19)		36.9 vs. 32.8	<0.001
Hiremath et al. (2019) [54]<br><br>Follow-up: 12 mo	α1-blocker vs. other BP-lowering drugs	Incidence of hypotension related events	HR 1.10 (1.01-1.20)		1,214 vs. 1,025	
	α1-blocker vs. other BP-lowering drugs	Incidence of Hypotension	HR 1.71 (1.33 – 2.20)		184 vs. 97	
	α1-blocker vs. other BP-lowering drugs	Incidence of Syncope	HR 1.44 (1.18 – 1.75)		263 vs. 170	
	α1-blocker vs. other BP-lowering drugs	Incidence of Falls	HR 1.02 (0.92 – 1.13)		760 vs. 687	
	α1-blocker vs. other BP-lowering drugs	Incidence of Fractures	HR 0.94 (0.82 – 1.08)		421 vs. 417	
	α1-blocker vs. other BP-lowering drugs	Adverse cardiac event	HR: 1.06 (0.99-1.13)		2,251 vs. 1,914	
	α1-blocker vs. other BP-lowering drugs	All-cause mortality	HR: 1.06 (0.95-1.20)		681 vs. 545	

Hundemer et al. (2021) [32]<br><br>Follow-up: Max. 3 y	α1-blocker vs. other BP-lowering drugs	≥30% eGFR decline/1,000 person-years	HR: 1.14 (1.08-1.21)		3,036 (12.1%) vs. 2,548 (10.7%)	<0.001
	α1-blocker vs. other BP-lowering drugs	Dialysis or kidneyTx/1,000 person-years	HR: 1.26 (1.13-1.44)		642 (1.52%) vs. 475 (1.14%)	<0.001
	α1-blocker vs. other BP-lowering drugs	Cardiac events/1,000 person-years	HR: 0.92 (0.89-0.95)		6,595 (20.7%) vs. 6,774 (22.4%)	<0.001
	α1-blocker vs. other BP-lowering drugs	Deaths/1,000 person-years	HR: 0.89 (0.84-0.94)		2,610 (6.07%) vs. 2,854 (6,76%)	<0.001
	α1-blocker vs. other BP-lowering drugs	Hypotension/1,000 person-years	HR: 1.08 (0.96-1.21)		647 (1.53%) vs. 593 (1.43%)	>0.05
	α1-blocker vs. other BP-lowering drugs	Syncope/1,000 person-years	HR: 1.23 (1.11-1.37)		816 (1.95%) vs. 656 (1.59%)	<0.001
	α1-blocker vs. other BP-lowering drugs	Falls/1,000 person-years	HR: 1.00 (0.94-1.06)		2,388 (6.00%) vs. 2,376 (6.07%)	>0.05
	α1-blocker vs. other BP-lowering drugs	Fractures/1,000 person-years	HR: 1.03 (0.95-1.12)		1,156 (2.79%) vs. 1,111 (2.72%)	>0.05
Siemens et al. (2021) [55]<br><br>Follow-up: Max. 13 y	α1-blocker use vs. no medication	Incidence of new cardiac failure	HR: 1.22 (1.18-1.26)			<0.001
	α1-blocker + 5-ARI combination vs. no medication	Incidence of new cardiac failure	HR: 1.16 (1.12-1.21)			<0.001
	Selective α1-blocker vs. non-selective α1-blocker	Incidence of new cardiac failure	HR: 1.08 (1.00-1.12)			0.04
Tae et al. (2019) [56]	Tamsulosin vs. no medication	Incidence of dementia	HR: 0.705 (0.635-0.782)		681 (20.4%) vs. 754 (22.6%)	<0.001

Follow-up: Mean (SD) days of follow up: 1,580 (674)	Doxazosin vs. no medication	Incidence of dementia	HR: 0.710 (0.637-0.792)		624 (21.1%) vs. 689 (23.3%)	<0.001
	Terazosin vs. no medication	Incidence of dementia	HR: 0.831 (0.749-0.921)		708 (22.5%) vs. 742 (23.6%)	0.001
	Alfuzosin vs. no medication	Incidence of dementia	HR: 0.682 (0.607-0.766)		529 (19.2%) vs. 623 (22.6%)	<0.001
	Medium dose level doxazosin vs. tamsulosin	Incidence of dementia	HR: 1.010 (0.906-1.126)			0.859
	Medium dose level terazosin vs. tamsulosin	Incidence of dementia	HR: 1.085 (0.949-1.240)			0.233
	Medium dose level alfuzosin vs. tamsulosin	Incidence of dementia	HR: 1.122 (0.950-1.324)			0.176
Welk et al. (2015) [51]<br><br>Follow-up:<br>90 days	α1-blocker use vs. no use	Falls	OR: 1.14 (1.07-1.21)		2,129 (1.45%) vs. 1,881 (1.28%)	<0.05
	α1-blocker use vs. no use	Fracture	OR: 1.16 (1.04-1.29)		699 (0.48%) vs. 605 (0.41%)	<0.05
	α1-blocker use vs. no use	Major osteoporotic fracture	OR: 1.05 (0.89-1.23)		312 (0.21%) vs. 298 (0.20%)	>0.05
	α1-blocker use vs. no use	Hip fracture	OR: 0.98 (0.78-1.21)		159 (0.11%) vs. 163 (0.11%)	>0.05
	α1-blocker use vs. no use	Hypotension	OR: 1.80 (1.59-2.03)		706 (0.48%) vs. 394 (0.27%)	<0.05
	α1-blocker use vs. no use	Head trauma	OR: 1.15 (1.04-1.27)		888 (0.60%) vs. 773 (0.53%)	<0.05

	α1-blocker use vs. no use	Falls in age group <75 y	OR: 1.17 (1.04-1.31)		603 (0.84%) vs. 515 (0.71%)	<0.05
	α1-blocker use vs. no use	Falls in age group ≥75 y	OR: 1.12 (1.04-1.21)		1,526 (2.04%) vs. 1,366 (1.82%)	<0.05
	Comparison α1-blocker use vs. no use between age groups	Falls < 75 y<br>vs.<br>Falls ≥75 y			603 (0.84%) vs. 515 (0.71%) vs. 1,526 (2.04%) vs. 1,366 (1.82%)	0.52
	Tamsulosin use vs. no use	Falls	OR: 1.12 (1.04-1.19)		1,810 (1.47%) vs. 1,625 (1.32%)	<0.05
	Alfuzosin use vs. no use	Falls	OR: 1.24 (1.04-1.48)		279 (1.34%) vs. 226 (1.09%)	<0.05
	Silodosin use vs. no use	Falls	OR: 1.35 (0.83-2.18)		40 (1.47%) vs. 30 (1.10%)	>0.05
	Comparison of fall-rates (substance vs. no substance) between substances	Falls tamsulosin use vs. no use<br>vs.<br>falls alfuzosin use vs. no use<br>vs.<br>falls silodosin use vs. no use	OR:<br>1.12 (1.04-1.19) vs.<br>1.24 (1.04-1.48) vs.<br>1.35 (0.83-2.18)			0.44
	Tamsulosin 0.4 mg vs. no use	Falls	OR: 1.09 (1.02-1.17)		1,622 (1.42%) vs. 1,488 (1.31%)	<0.05
	Tamsulosin 0.8 mg vs. no use	Falls	OR: 1.21 (0.91-1.62)		105 (1.55%) vs. 87 (1.28%)	>0.05

	Comparison of fall-rates (substance vs. no substance) according to drug administration	Falls tamsulosin 0.4 mg vs. no use<br>vs.<br>Falls tamsulosin 0.8 mg vs. no use	OR: 1.09 (1.02-1.17)<br>vs.<br>1.21 (0.91-1.62)			0.49
**Case-control studies:**						
Hall and McMahon (2007) [52]<br><br>Follow-up:<br>Mean (SD) days of observation:<br>· Cases: 569 (344)<br>· Controls: 569 (344)	Fractures vs. no fractures total	Current doxazosin use	OR: 0.82 (0.63-1.08)<br><br>Adj. OR: 0.90 (0.68-1.19)		66 (1.01%) vs. 311 (1.17%)	>0.05
	Fractures vs. no fractures	Doxazosin treatment started within 28 days	OR: 0.57 (0.17-1.92)		3 (0.05%) vs. 20 (0.08%)	>0.05
	Fractures vs. no fractures	Doxazosin treatment started within 84 days	OR: 0.48 (0.23-1.01)		8 (0.12%) vs. 65 (0.25%)	>0.05
	Fractures vs. no fractures male ≤75a	Current doxazosin use	OR: 0.84 (0.39-1.78)		9 (1.17%) vs. 39 (1.19%)	>0.05
	Fractures vs. no fractures female ≤75a	Current doxazosin use	OR: 1.08 (0.68-1.72)		25 (0.97%) vs. 99 (0.95%)	>0.05
	Fractures vs. no fractures male >75a	Current doxazosin use	OR: 0.61 (0.20-1.83)		4 (0.92%)<br>20 (1.08%) vs.	>0.05
	Fractures vs. no fractures female >75a	Current doxazosin use	OR: 0.73 (0.48-1.10)		28 (1.01%) vs. 153 (1.39%)	>0.05
	Fractures vs. no fractures total	Any previous doxazosin use	OR: 0.84 (0.67-1.04)		99 (1.51%) vs. 468 (1.77%)	>0.05

			Adj. OR: 0.92 (0.69-1.23)			
	Fractures vs. no fractures male ≤75a	Any previous doxazosin use	OR: 0.94 (0.50-1.75)		13 (1.68%) vs. 56 (1.71%)	>0.05
	Fractures vs. no fractures female ≤75a	Any previous doxazosin use	OR: 0.95 (0.65-1.38)<br><br>Adj. OR: 1.00 (0.67-1.48)		37 (1.44%) vs. 163 (1.57%)	>0.05
	Fractures vs. no fractures male >75a	Any previous doxazosin use	OR: 0.95 (0.42-2.14)		8 (1.83%) vs. 28 (1.51%)	>0.05
	Fractures vs. no fractures female >75a	Any previous doxazosin use	OR: 0.75 (0.53-1.05)<br><br>Adj. OR: 0.83 (0.58-1.17)		41 (1.48%) vs. 221 (2.01%)	>0.05
	Fractures vs. no fractures (no current exposure to doxazosin)	Current exposure to alpha-1 antagonist other than doxazosin	OR: 0.89 (0.71-1.12)<br><br>Adj. OR: 0.93 (0.73-1.18)		94 (1.44%) vs. 446 (1.68%)	>0.05
	Fractures vs. no fractures (no current exposure to doxazosin)	Treatment with other alpha1-antagonist started within 28 days	OR: 1.42 (0.65-3.07)		9 (0.14 %) vs. 28 (0.11%)	>0.05
	Fractures vs. no fractures (no current exposure to doxazosin)	Treatment with other alpha1-antagonist started within 84 days	OR: 1.46 (0.88-2.42)		21 (0.32%) vs. 66 (0.25%)	>0.05

Testa et al. (2018) [53]<br><br>Follow-up: 3 months	Syncope due to orthostatic hypotension (OH) vs. non-OH syncope	α1-blocker use	RR: 1.67 (1.00-2.85)<br><br>Adj. for age and sex: RR: 1.48 (0.84-2.60)		28/170 (16.5%) vs. 18/184 (9.8%)	0.043
	Syncope due to orthostatic hypotension (OH) vs. non-OH syncope	α1-blocker + diuretic	RR: 1.70 (1.04-2.78)<br><br>Adj. for age and sex:<br>RR: 1.83 (0.85-3.96)		14/170 (8.2%) vs. 6/184 (3.3%)	0.036

Table S3 Detailed summary of study findings of included meta-analyses

Authors (Year)<br>Follow-up period	Drug vs. comparator	Outcome	Relative Risk (RR)/Odds Ratio (OR)/Hazard Ratio (HR)	Mean difference (95% CI)	Events (%) or mean score (SD)	p-value
Buzelin et al. (1997) [57]<br><br>Follow-up: 1 month	Alfuzosin vs. placebo	ADEs ≥65 y			12/149 (8.1%) vs. 12/153 (7.8%)	>0.05
	Alfuzosin vs. placebo	ADEs related to vasodilation ≥65 y			2/149 (1.3%) vs. 2/153 (1.3%)	>0.05
Lowe (1994) [58]<br><br>Follow-up: 2-6 months	Terazosin vs. placebo in the age group 65 y – 74 y	Dizziness			25/235 (9.8%) vs. 7/143 (4.9%)	>0.05
		Asthenia			16/235 (6.8%) vs. 1/143 (0.7%)	<0.05
		Headache			17/235 (6.8%) vs. 5/143 (3.5%)	>0.05
		Postural symptoms			15/235 (6.0%) vs. 2/143 (1.4%)	<0.05
		Somnolence			9/235 (3.8%) vs. 4/143 (2.8%)	>0.05
		Nasal congestion			5/235 (2.1%) vs. 0/143 (0.0%)	>0.05
		Nausea			4/235 (1.7%) vs. 1/143 (0.7%)	>0.05
		Impotence			4/235 (1.7%) vs. 1/143 (0.7%)	>0.05

		Blurred vision			6/235 (2.6%) vs. 0/143 (0.0%)	<0.05
		Syncope			3/235 (1.3%) vs. 0/143 (0.0%)	>0.05
	Terazosin vs. placebo in the age group >74 y	Dizziness			6/50 (12%) vs. 0/19 (0%)	<0.05
		Asthenia			2/50 (4.0%) vs. 1/19 (5.3%)	>0.05
		Headache			0/50 (0.0%) vs. 1/19 (5.3%)	>0.05
		Postural symptoms			2/50 (4.0%) vs. 0/19 (0.0%)	>0.05
		Somnolence			2/50 (4.0%) vs. 1/19 (5.3%)	>0.05
		Nasal congestion			1/50 (2.0%) vs. 0/19 (0.0%)	>0.05
		Nausea			0/50 (0.0%) vs. 1/19 (5.3%)	>0.05
		Impotence			0/50 (0.0%) vs. 0/19 (0.0%)	>0.05
		Blurred vision			0/50 (0.0%) vs. 1/19 (5.3%)	>0.05
		Syncope			0/50 (0.0%) vs. 0/19 (0.0%)	>0.05
Chapple et al. (1997) [59]	Tamsulosin vs. placebo in the age group ≥65 y	Any adverse event			70/191 (37%) vs. 31/100 (31%)	0.330
		Drug related adverse event[1]			23/191 (12%) vs. 9/100 (9%)	0.459

Follow-up: 12 weeks		Discontinued due to adverse events			7/191 (3.7%) vs. 4/100 (4%)	1.000
		Serious adverse events			5/191 (2.6%) vs. 4/100 (4%)	0.499
		Adverse events possibly associated with vasodilation[2]			8/191 (4.2%) vs. 6/100 (6%)	0.523
		Adverse events possibly associated with vasodilation[2] and drug related[1]			7/191 (3.7%) vs. 3/100 (3%)	0.767
		Abnormal ejaculation			5/191 (2.6%) vs. 1/100 (1.0%)	0.668
		Dizziness			5/191 (2.6%) vs. 3/100 (3.0%)	1.000
		Flu syndrome			5/191 (2.6%) vs. 3/100 (3.0%)	1.000
		Infection			4/191 (2.1%) vs. 1/100 (1.0%)	0.663

[1] Decision taken by the investigator: possibly or probably drug related.

[2] Includes dizziness, headache, tachycardia, palpitation, postural hypotension and syncope.
